# Shared genetic architecture of hernias: A genome-wide association study with multivariable meta-analysis of multiple hernia phenotypes

**DOI:** 10.1371/journal.pone.0272261

**Published:** 2022-12-30

**Authors:** Waheed Ul-Rahman Ahmed, Manal I. A. Patel, Michael Ng, James McVeigh, Krina Zondervan, Akira Wiberg, Dominic Furniss

**Affiliations:** 1 Botnar Research Centre, Nuffield Department of Orthopaedics, Rheumatology and Musculoskeletal Sciences, University of Oxford, Oxford, United Kingdom; 2 Nuffield Department of Women’s & Reproductive Health, John Radcliffe Hospital, University of Oxford, Oxford, United Kingdom; 3 Wellcome Centre for Human Genetics, University of Oxford, Oxford, United Kingdom; 4 Department of Plastic and Reconstructive Surgery, Oxford University Hospitals NHS Foundation Trust, Oxford, United Kingdom; Stellenbosch University Faculty of Medicine and Health Sciences, SOUTH AFRICA

## Abstract

Abdominal hernias are common and characterised by the abnormal protrusion of a viscus through the wall of the abdominal cavity. The global incidence is 18.5 million annually and there are limited non-surgical treatments. To improve understanding of common hernia aetiopathology, we performed a six-stage genome-wide association study (GWAS) of 62,637 UK Biobank participants with either single or multiple hernia phenotypes including inguinal, femoral, umbilical and hiatus hernia. Additionally, we performed multivariable meta-analysis with metaUSAT, to allow integration of summary data across traits to generate combined effect estimates. On individual hernia analysis, we identified 3404 variants across 38 genome-wide significant (p < 5×10^−8^) loci of which 11 are previously unreported. Robust evidence for five shared susceptibility loci was discovered: *ZC3H11B*, *EFEMP1*, *MHC* region, *WT1* and *CALD1*. Combined hernia phenotype analyses with additional multivariable meta-analysis of summary statistics in metaUSAT revealed 28 independent (seven previously unreported) shared susceptibility loci. These clustered in functional categories related to connective tissue and elastic fibre homeostasis. Weighted genetic risk scores also correlated with disease severity suggesting a phenotypic-genotypic severity correlation, an important finding to inform future personalised therapeutic approaches to hernia.

## Introduction

A hernia is the abnormal protrusion of a viscus through the wall of the anatomic cavity in which it is normally enclosed. Abdominal wall hernia (AWH) represent the majority of hernia phenotypes, with the lifetime risk for abdominal hernia being 27% in men and 3% in women [1]. Globally, at least 20 million AWHs are repaired every year, and there is an associated annual mortality of 59,800 deaths [2, 3].

Surgery is the definitive treatment for symptomatic AWH. However, management is often challenging with significant risk of complications including chronic pain, seroma, haematoma, infection and failure of surgical repair [4, 5]. In the case of femoral hernia, diagnostic difficulties lead to up to 40% of cases presenting as bowel strangulation or obstruction requiring emergency repair, which is associated with high mortality [6, 7]. Therefore, there is a need to improve understanding of hernia aetiopathology, to guide new therapeutic avenues and improve patient outcomes. Indeed, patients with a family history carry an eight-fold risk of groin hernia and are more likely to suffer from contralateral or recurrent inguinal hernia as well as other hernia pathology including femoral, umbilical, incisional and epigastric hernia [8].

There is evidence for a genetic predisposition to AWH. Groin hernias have previously been shown to cluster in families [9], while the characteristic presence of hernia in several connective tissue disorders including Marfan’s, Ehlers Danlos and Cutis laxa suggests an underlying genetic basis relating to impaired homeostasis of the extracellular matrix (ECM) [10–12]. Previously, Jorgenson *et al*. have identified four susceptibility loci for inguinal hernia alone (*WT1*, *EFEMP1*, *EBF2* and *ADAMTS6*), each of which may result in aberrant elastic tissue homeostasis mediated via disordered expression of matrix metalloproteinases (MMPs) [13]. A further trans-ethnic GWAS meta-analysis of inguinal hernia identified five further loci including *TGFB2*, *HMCN2* and *CDKN3* [14]. Wei *et al*. attempted to characterise the polygenetic architecture of hernia using individual GWAS analysis of patients with either inguinal, femoral, umbilical or ventral hernia, identifying 57 loci, highlighting *AIG1* and *CALD1* as candidate genes for shared hernia susceptibility [15]. Interestingly, to our knowledge, GWAS of hiatus hernia has previously not been reported.

Here, we perform a comprehensive analysis to further characterise the shared genetic architecture between four hernia phenotypes including inguinal, femoral, umbilical and hiatus hernia, utilising both individual and combined genome-wide association studies. We further utilised multivariable meta-analysis in metaUSAT [16] a data-adaptive method, robust to the association structure of correlated traits, to perform a unified association test for each SNP across several trait summary statistics.

## Results

**Fig 1:**
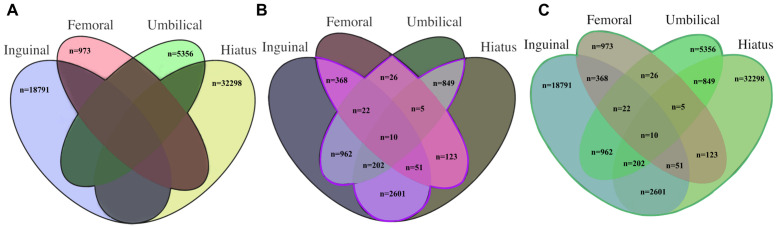
Individual, overlap and umbrella case-control cohorts in the UK Biobank. A: Individual hernia cohort: 57,418 individual hernia cases were matched to 287,090 controls. Participants with more than one hernia phenotype coding were excluded (dark shading). B: Overlap hernia cohort: 5,219 participants possessed coding for at least 2 hernia phenotypes and were included in the overlap hernia cohort and matched with 26,095 non-hernia controls. All cases with single phenotype coding were excluded (dark shading). C: Umbrella hernia cohort, 62,637 participants had diagnostic or operative coding for any hernia type including those with single or multiple hernia and were matched to 313,185 non-hernia controls.

Following quality control, a total of 62,637 individuals in the UK Biobank possessed a diagnostic and/or operative code for at least one of the hernia subtypes studied. Participants were divided into three hernia cohorts (individual hernia cohorts, overlap hernia cohort, and umbrella hernia cohort) as shown in Fig 1. Each was matched 1:5 to non-hernia controls in UK Biobank based on age (+/- 5 years), sex and genotyping platform, while ensuring that control cohorts for the four individual hernia analyses contained completely distinct individuals. Sex distributions for all hernia cohorts are shown in S1 Table.

### Individual hernia cohorts

These were four cohorts of participants who had diagnostic and/or operative coding for only one of the four hernia phenotypes. In these analyses, 23,007 individuals had diagnostic or operative codes for inguinal hernia, 1,578 for femoral hernia, 7,432 for umbilical hernia and 36,138 for hiatus hernia. The final individual hernia cohorts were then defined by removing participants with multiple hernia phenotypes (4,216 from the inguinal hernia cohort, 605 from the femoral hernia cohort, 2,076 from the umbilical hernia cohort and 3,841 from the hiatus hernia cohort), to create a phenotypically ‘clean’ cohort for each hernia subtype.

The final Individual hernia cohorts therefore consisted of the following individuals.

- Inguinal hernia: 18,791 cases and 93,955 controls (N_effective_ = 62,637)
- Femoral hernia: 973 cases and 4,865 controls (N_effective_ = 3,243)
- Umbilical hernia: 5,356 cases and 26,780 controls (N_effective_ = 17,853)
- Hiatus hernia: 32,298 cases and 161,490 controls (N_effective_ = 107,660)

### Overlap hernia cohort

The overlap hernia cohort consisted of participants with diagnostic or operative codes for two or more hernia subtypes. There were 5,219 cases which were matched to 26,095 non-hernia controls (total cohort 31,314 individuals).

### Umbrella hernia cohort

The umbrella hernia cohort involved all participants who had diagnostic and/or operative codes for *any* hernia subtype, including those with single or multiple hernia subtypes. The umbrella hernia cohort consisted of 62,637 cases who were matched 313,185 non-hernia controls (total cohort 375,822 individuals).

**Fig 2:**
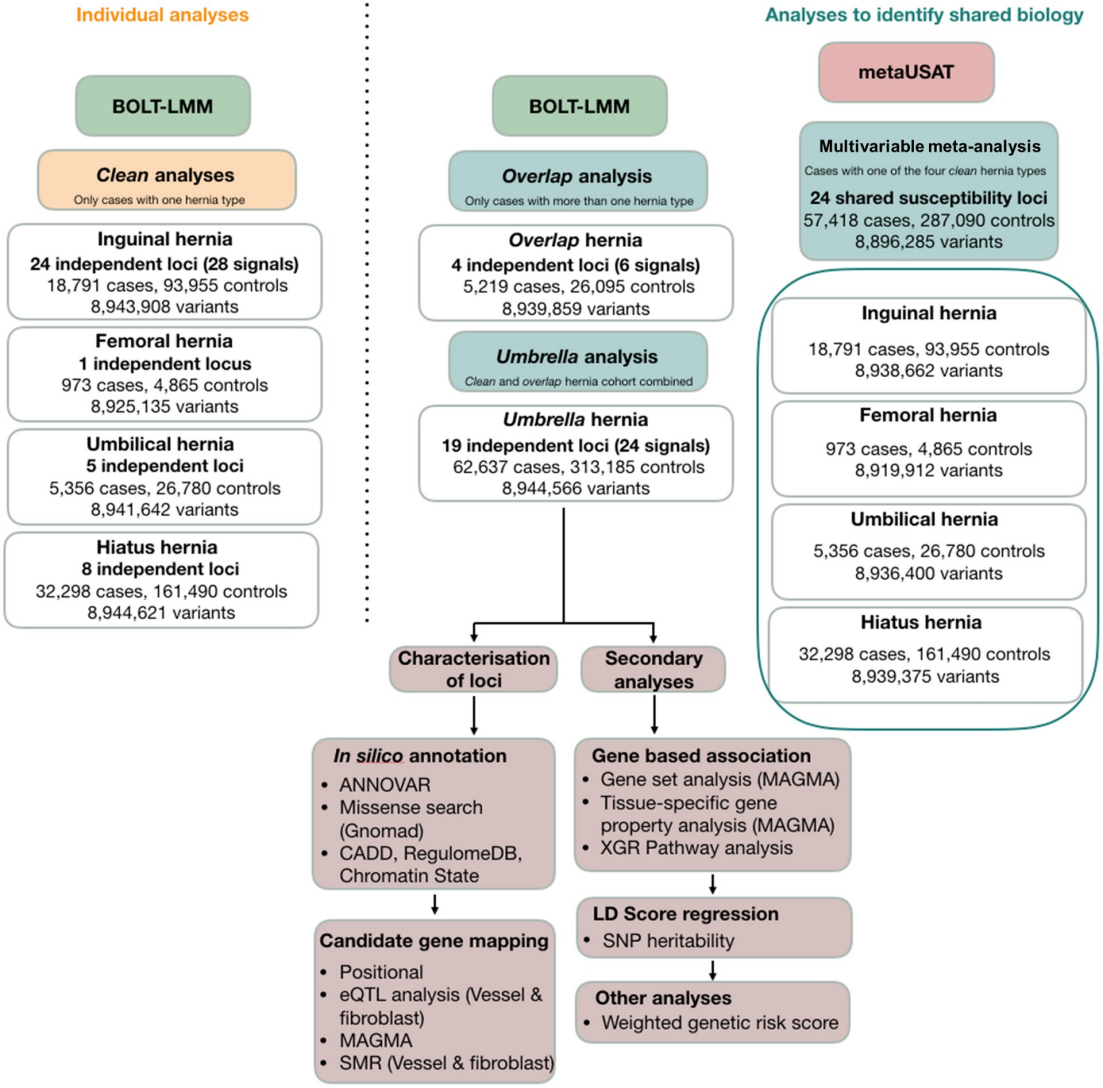
Study design and shared genetics analysis workflow. The three analysis approaches to characterise the shared genetic underpinnings of the ‘hernia phenotype’ are depicted.

The analytic workflow representing the three analyses implemented to characterise shared genetic underpinnings of AWH are depicted in Fig 2.

### Individual hernia cohort analyses

Initially, four separate GWAS analyses were undertaken in order to identify genetic risk loci for inguinal, femoral, umbilical or hiatus hernia in participants affected *only* by a single hernia subtype (termed ‘Individual hernia cohorts’). We cumulatively discovered genome-wide significant associations at 3,404 variants across 38 loci (52 independent signals), with 24 susceptibility loci for inguinal hernia, one locus for femoral hernia, five loci for umbilical hernia, and eight loci for hiatus hernia (S2–S5 Tables and S1 Fig). Results relating to in silico annotation and candidate gene mapping of individual hernia loci are given in S6–S13 Tables).

**Fig 3:**
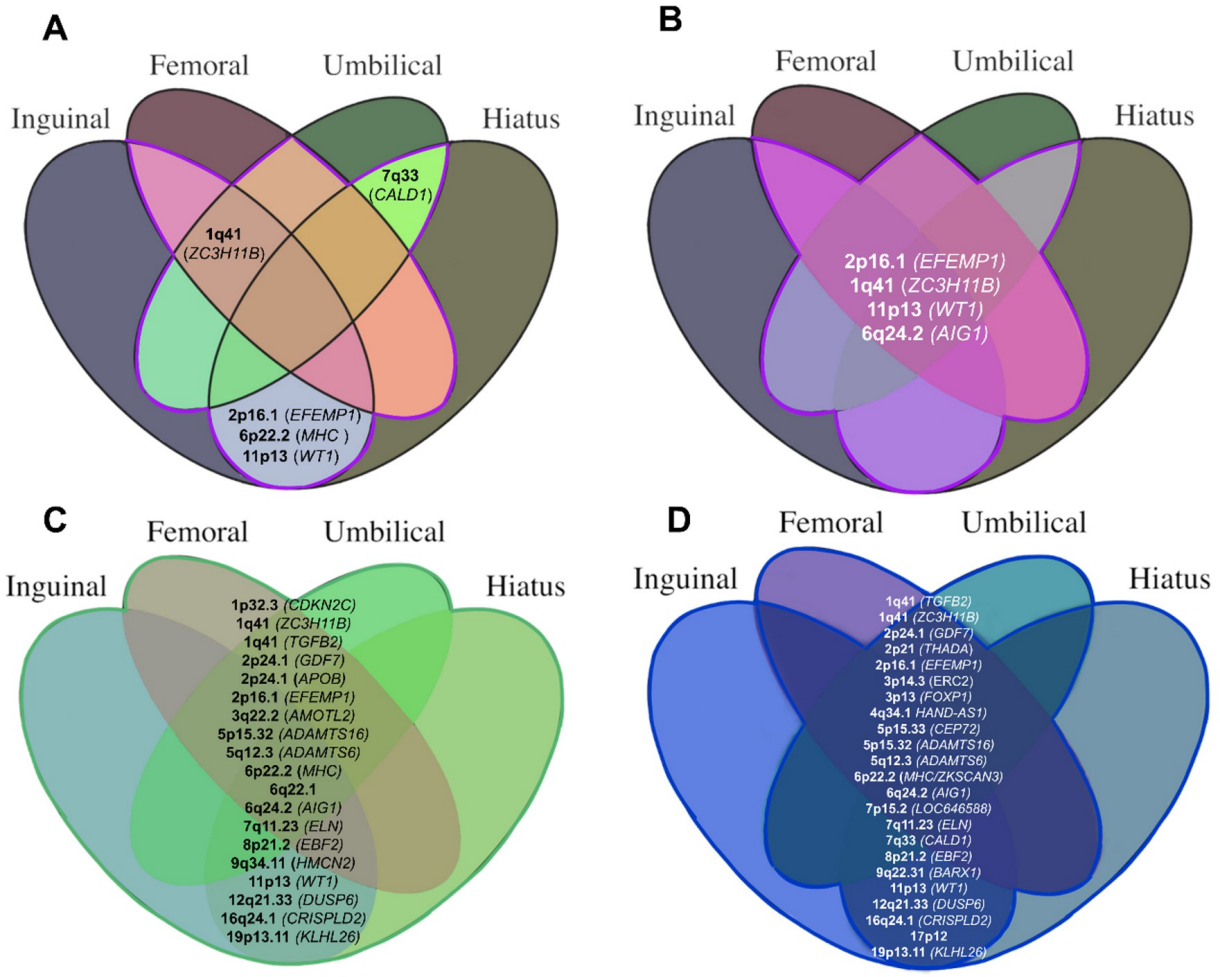
Shared susceptibility loci from individual, overlap and umbrella GWAS analyses as well as multivariable meta-analysis in metaUSAT. A: Individual hernia cohort: Evidence of shared susceptibility was demonstrated at five loci. B: Overlap hernia cohort: Evidence of shared susceptibility demonstrated at four loci. C: Umbrella hernia cohort: 19 loci became significant under umbrella hernia analysis. D: Multivariable meta-analysis in metaUSAT 24 loci were significant under meta-analysis (two loci at 5p15.32).

We discovered evidence for five shared susceptibility loci (1q41 (*ZC3H11B*); 2p16.1 (*EFEMP1*); 6p22.2 (MHC region); 11p13 (*WT1*); 7q33 (*CALD1*)) amongst the individual hernia cohorts, of which four demonstrated concordance in allelic effect directions, as depicted in Table 1 and Fig 3. Q-Q plots for individual hernia GWAS analyses are shown in S2 Fig. Regional Locus Zoom plots for all individual hernia associations are provided in S3 Fig).

**Table 1:** Five shared susceptibility loci observed between individual hernia cohorts.

Chr	Mapped genes^a^	Inguinal				Femoral				Umbilical				Hiatus			
		rsID	p-value	OR (95% CI)	EAF^b^	rsID	p-value	OR (95% CI)	EAF^b^	rsID	p-value	OR (95% CI)	EAF^b^	rsID	p-value	OR (95% CI)	EAF^b^
1q41	*ZC3H11B*	rs2820441	6.6×10^−13^	1.09 (1.06–1.11)	0.32(C)	rs7538503	1.3×10^−10^	1.42 (1.27–1.58)	0.29(G)	rs4846567	1.7×10^−18^	1.22 (1.17–1.28)	0.31 (T)	-	^-^	-	-
2p16.1	*EFEMP1*	rs11899888	2.2×10^−12^	1.16 (1.13–1.20)	0.16(G)	-	-	-	-	-	-	-	-	rs10207635	1.3×10^−8^	1.07 (1.05–1.10)	0.13(T)
6p22.2	*MHC region*	rs13212652	3.1×10^−11^	1.12 (1.08–1.15)	0.87(T)	-	-	-	-	-	-	-	-	rs9393735	2.7×10^−8^	1.07 (1.05–1.10)	0.86(A)
7q33	*CALD1*	-	-	-	-	-	-	-	-	rs12707188	5.0×10^−15^	1.19 (1.14–1.24)	0.37 (T)	rs4728341	3.9×10^−10^	1.06 (1.04–1.07)	0.55(T)
11p13	*WT1*	rs4140413	2.4×10^−20^	1.11 (1.09–1.14)	0.63(G)	-	-	-	-	-	-	-	-	rs11031796	3.6×10^−16^	1.07 (1.06–1.09)	0.62(G)

^a^The genes prioritised at these loci based on positional mapping, eQTL mapping, MAGMA gene mapping and summary-based mendelian randomisation (see Methods).

^b^ The effect allele frequency. The effect allele is in brackets

### Overlap hernia cohort analysis

**Fig 4:**
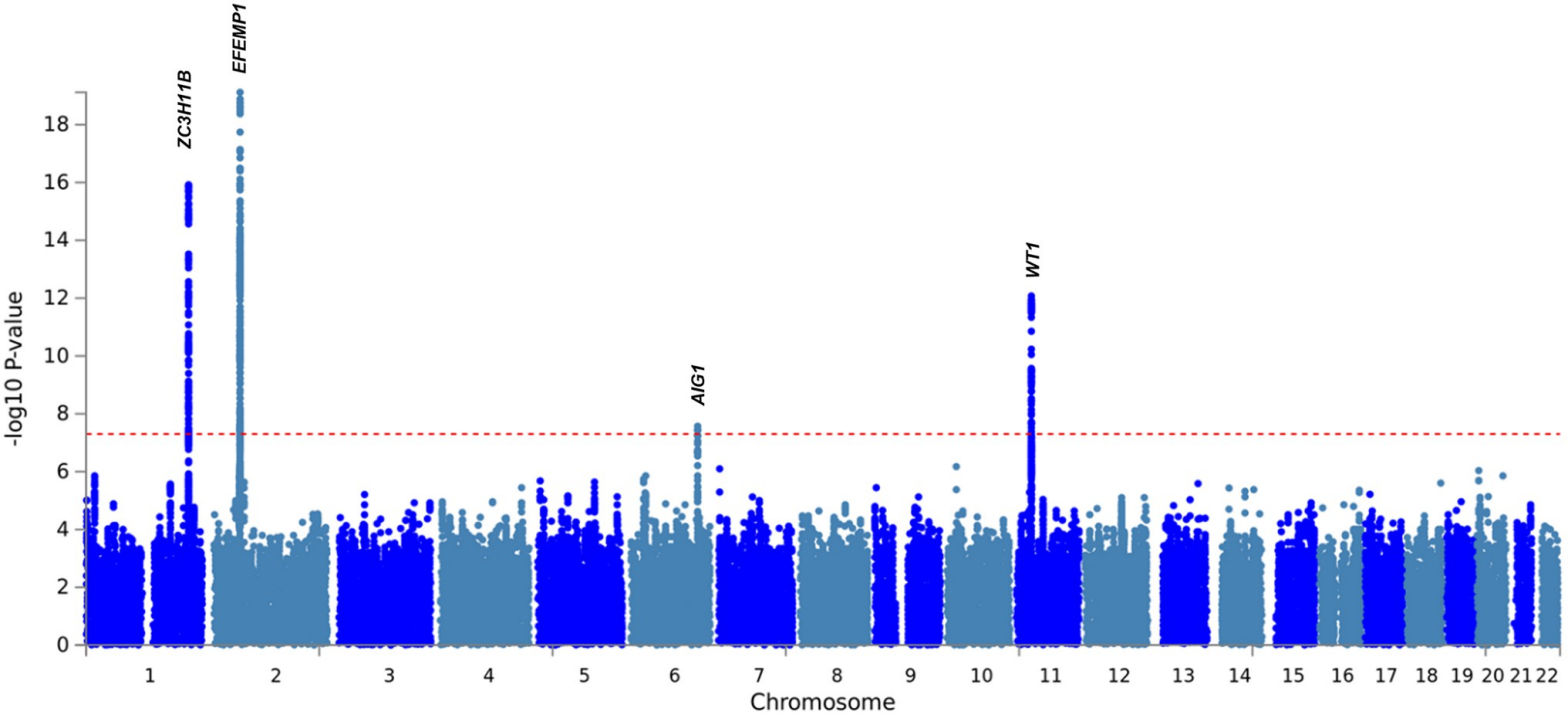
Manhattan plot of the overlap hernia cohort GWAS analyses. Manhattan plot is annotated with the gene names of loci that demonstrated shared susceptibility across two or more individual analyses.

We performed a further GWAS across participants with diagnostic or operative codes for at least two hernia subtypes (overlap hernia cohort; Fig 1). Significant associations at four loci (six independent signals) were revealed to confer shared susceptibility to multiple individual hernia phenotypes (Table 2 and Figs 3 and 4). The strongest association was observed at 2p16.1 (*EFEMP1*), closely followed by 1q41 (*ZC3H11B*) and 11p13 (*WT1*) which were all identified as shared susceptibility loci on analysis of the individual hernia cohorts. The fourth locus identified was 6q24.2 (*AIG1*) (rs4896643, p = 3.6×10^−8^, OR = 1.12) also identified in the inguinal individual hernia cohort (p = 7.8×10^−13^, OR = 1.08).

**Table 2:** Four loci significantly associated with overlap hernia in 5,219 cases and 26,095 controls in UK Biobank.

Chromosome	Position^a^	rsID	EA^b^	NEA^c^	EAF^d^	Info^e^	OR (95% CI)	P-value	Mapped genes^f^
1q41	219742537	rs1415287	T	C	0.31	0.998	1.21 (1.16–1.26)	1.2×10^−16^	-
2p16.1^#^	56040099	rs10199082	C	T	0.14	G	1.29 (1.22–1.37)	1.9×10^−10^	*EFEMP1*
2p16.1	56108333	rs1346786	C	T	0.71	0.994	1.24 (1.18–1.29)	7.6×10^−20^	*EFEMP1*
2p16.1^#^	56194773	rs981037	T	C	0.58	0.993	1.21 (1.16–1.27)	1.0×10^−9^	-
6q24.2	143670001	rs4896643	C	G	0.45	0.993	1.12 (1.08–1.17)	3.6×10^−8^	*AIG1*
11p13	32484594	rs3858458	C	T	0.63	0.981	1.17 (1.12–1.22)	8.4×10^−13^	*WT1*

^a^Based on NCBI Genome Build 37 (hg19).

^b^The effect allele.

^c^The non-effect allele.

^d^The effect allele frequency.

^e^The SNP INFO score for imputed SNPs; G = genotyped SNP.

^f^The four genes prioritised at these loci based on positional mapping, eQTL mapping and MAGMA gene mapping (see Methods).

^#^Denotes the two residual significant signals following conditional regression analysis at the lead SNP at the locus.

### Umbrella hernia cohort analysis

**Fig 5:**
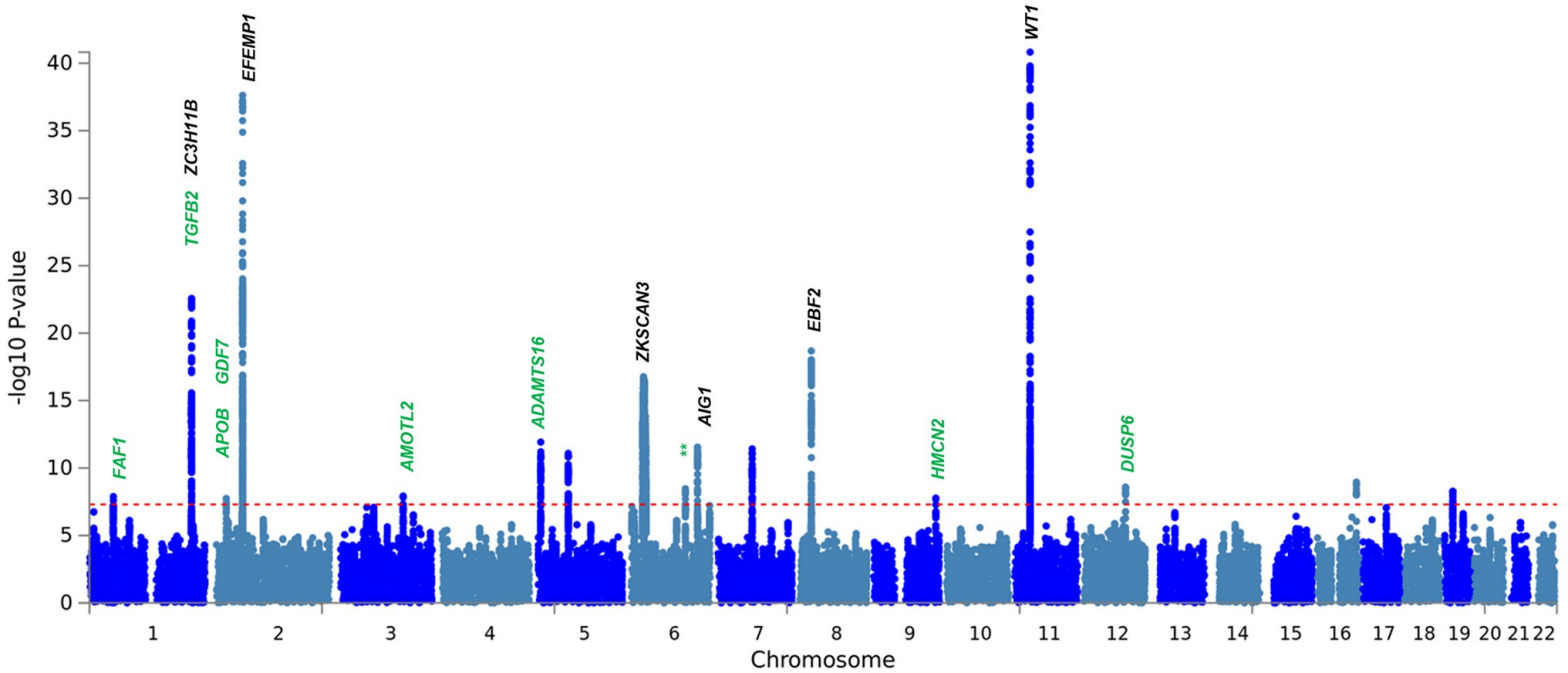
Manhattan plot of the umbrella hernia cohort GWAS analyses. Annotations show the gene names of loci that demonstrated shared susceptibility across two or more individual analyses. The nine loci that were not discovered in the individual or overlap analyses are highlighted with green font or with ** where no gene was prioritised at this locus.

A sixth GWAS of all UK Biobank participants affected by hernia, single or multiple (‘umbrella hernia cohort’) demonstrated 19 genome-wide significant loci representing 25 independent signals. Eleven loci were previously identified in the individual or overlap cohorts (Table 3, Figs 3 and 5) of which five were strongly associated in the umbrella analysis: 11p13 (*WT1*; rs66798575, p_umbrella_ = 1.6×10^−40^), 2p16.1 (E*FEMP1*; rs75439645, p_umbrella_ = 2.4×10^−38^), 1q41 (*ZC3H11B*; rs2820441 p_umbrella_ = 2.7×10^−23^), 6p22.2 (MHC region) (rs28360634, p_umbrella_ = 1.7×10^−17^), and 6q24.2 (*AIG1*; rs6917403, p_umbrella_ = 2.9×10^−12^). Eight loci, previously undiscovered on analysis of individual and overlap cohorts, were also discovered, with the strongest association amongst these observed at 1q41 (*TGFB2*; rs2799098, p_umbrella_ = 9.3×10^−15^, OR = 1.06). Q-Q plots for overlap and umbrella hernia cohorts are shown in S4 Fig. Regional Locus Zoom Plots for all for all associations are provided in S5 and S6 Figs).

**Table 3:** Nineteen loci (twenty-five signals) significantly associated in the umbrella hernia analysis.

Chr	Position^a^	rsID	EA^b^	NEA^c^	EAF^d^	Info^e^	OR (95% CI)	P-value	Mapped genes^f^	Significant in individual or overlap GWAS^g^
**1p32.2**	**51477643**	**rs13376700**	**A**	**T**	**0.43**	**0.992**	**1.04 (1.02–1.05)**	**1.3×10** ^**−8**^	***CDKN2C*, *EPS15*, *FAF1*, *NRD1***	-
1q41	218521609	rs2799098	A	G	0.82	G	1.06 (1.05–1.08)	9.3×10^−15^	*RRP15*, *TGFB2*	-
1q41	219734960	rs2820441	C	A	0.32	G	1.07 (1.05–1.08)	2.7×10^−23^	-	*IH*,*FH*,*UH*,*OH*
**2p24.1**	**20878406**	**rs3072**	**C**	**T**	**0.36**	**0.994**	**1.04 (1.02–1.05)**	**1.8×10** ^**−8**^	***C2orf43*, *GDF7***	-
**2p24.1**	**21239884**	**rs76622701**	**A**	**T**	**0.56**	**0.979**	**1.04 (1.02–1.05)**	**3.5×10** ^**−8**^	***APOB***	-
2p16.1	56048944	rs75439645	A	G	0.13	0.999	1.12 (1.10–1.14)	2.4×10^−38^	*CCDC104*, *EFEMP1*, *PNPT1*, *SMEK2*	*IH*,*HH*,*OH*
2p16.1^#^	56106928	rs59985551	C	T	0.78	0.998	1.09 (1.08–1.11)	5.1×10^−9^	*CCDC104*, *CLHC1*, *EFEMP1*, *PNPT1*, *RTN4*, *SMEK2*	*IH*,*HH*,*OH*
2p16.1^#^	56193665	rs13431149	C	A	0.60	0.991	1.07 (1.05–1.08)	2.5×10^−23^	*CCDC104*, *PNPT1*, *SMEK2*	*IH*,*HH*,*OH*
**3q22.2**	**134372486**	**rs9883955**	**G**	**T**	**0.63**	**G**	**1.04 (1.02–1.05)**	**1.2×10** ^**−8**^	***AMOTL2*, *ANAPC13*, *CEP63*, *EPHB1*, *KY***	-
**5p15.32** ^#^	**4881885**	**rs570260**	**G**	**A**	**0.34**	**G**	**1.03 (1.02–1.05)**	**6.2×10** ^**−10**^	-	***HH***
**5p15.32** ^#^	**4977446**	**rs42202**	**A**	**G**	**0.08**	**0.986**	**1.08 (1.06–1.11)**	**1.7×10** ^**−11**^	-	***HH***
**5p15.32** ^#^	**5145100**	**rs1834922**	**G**	**A**	**0.35**	**0.999**	**1.04 (1.02–1.05)**	**9.0×10** ^**−10**^	***ADAMTS16***	-
**5p15.32**	**5350637**	**rs7715383**	**C**	**G**	**0.10**	**0.970**	**1.08 (1.06–1.10)**	**1.2×10** ^**−12**^	-	-
5q12.3	64355060	rs370763	A	T	0.67	0.998	1.05 (1.03–1.06)	8.3×10^−12^	*ADAMTS6*	*IH*
6p22.1	27332891	rs28360634	T	C	0.89	1.000	1.09 (1.07–1.11)	1.7×10^−17^	*ABT1*, *APOM*, *APOM*, *BTN1A1*, *BTN2A1*, *BTN2A2*, *BTN3A1*, *BTN3A2*, *BTN3A3*, *C4A*, *C4B*, *C6orf15*, *C6orf48*, *CCHCR1*, *CLIC1*, *DDR1*, *DPCR1*, *HCG27*, *HFE*, *HIST1H1A*, *HIST1H1B*, *HIST1H2AG*, *HIST1H2AI*, *HIST1H2AJ*, *HIST1H2AK*, *HIST1H2AL*, *HIST1H2AM*, *HIST1H2BC*, *HIST1H2BF*, *HIST1H2BJ*, *HIST1H2BL*, *HIST1H2BM*, *HIST1H2BN*, *HIST1H2BO*, *HIST1H3C*, *HIST1H3H*, *HIST1H3I*, *HIST1H3J*, *HIST1H4A*, *HIST1H4J*, *HIST1H4K*, *HIST1H4L*, *HLA-A*, *HLA-B*, *HLA-C*, *HLA-DMA*, *HLA-DMB*, *HLA-DRA*, *HMGN4*, *LRRC16A*, *LSM2*, *MSH5*, *MSH5-SAPCD1*, *NKAPL*, *OR12D3*, *OR2B2*, *OR2B6*, *PBX2*, *PGBD1*, *POM121L2*, *POU5F1*, *PRRC2A*, *PRSS16*, *PSORS1C1*, *RNF5*, *SCAND3*, *SFTA2*, *SLC17A1*, *SLC17A2*, *SLC17A3*, *SLC17A4*, *TRIM26*, *TRIM27*, *TRIM31*, *TRIM38*, *TRIM39*, *TRIM39-RPP21*, *TUBB*, *VARS*, *VWA7*, *ZFP57*, *ZKSCAN3*, *ZKSCAN4*, *ZKSCAN8*, *ZNF165*, *ZNF184*, *ZNF192P1*, *ZNF322*, *ZNF391*, *ZSCAN12*, *ZSCAN16*, *ZSCAN23*, *ZSCAN31*, *ZSCAN9*	*IH*,*HH*
6q22.1	117507982	rs200889152	C	A	0.38	0.991	1.04 (1.03–1.05)	3.4×10^−9^	-	-
6q24.2	143653287	rs6917403	A	G	0.42	0.987	1.04 (1.03–1.06)	2.9×10^−12^	*AIG1*	*IH*,*OH*
7q11.23^#^	73445942	rs2356532	G	A	0.06	0.996	1.09 (1.06–1.11)	1.1×10^−8^	*ELN*	*IH*
7q11.23	73474825	rs17855988	G	C	0.90	0.963	1.08 (1.05–1.10)	3.8×10^−12^	*ELN*, *LIMK1*	*IH*
8p21.2	25693744	rs4368985	T	A	0.40	0.997	1.06 (1.05–1.07)	2.1×10^−19^	*EBF2*	*IH*
9q34.11	133038387	rs9299329	G	A	0.50	0.979	1.04 (1.02–1.05)	1.7×10^−8^	*HMCN2*	-
11p13	32451920	rs66798575	T	G	0.64	0.973	1.09 (1.08–1.10)	1.6×10^−40^	*CCDC73*, *EIF3M*, *WT1*	*IH*, *HH*,*OH*
**12q21.33**	**89767237**	**rs797267**	**G**	**A**	**0.19**	**0.996**	**1.05 (1.03–1.06)**	**2.6×10** ^**−9**^	***DUSP6***	-
16q24.1	84855477	rs1874013	G	T	0.38	0.994	1.04 (1.03–1.05)	1.1×10^−9^	*CRISPLD2*	*IH*
**19p13.11**	**18824038**	**rs34482977**	**C**	**G**	**0.81**	**0.992**	**1.05 (1.03–1.06)**	**5.3×10** ^**−9**^	***CRLF1*, *CRTC1*, *KLHL26*, *SSBP4***	***HH***

^a^Based on NCBI Genome Build 37 (hg19).

^b^The effect allele.

^c^The non-effect allele.

^d^The effect allele frequency.

^e^The SNP INFO score for imputed SNPs; G = genotyped SNP.

^f^The 138 genes prioritised at these loci based on positional mapping, eQTL mapping, MAGMA gene mapping and summary-based mendelian randomisation.

^g^where IH = Inguinal Hernia Individual, FH = Femoral Hernia Individual, UH = Umbilical Hernia Individual, HH = Hiatus Hernia Individual, OH = overlapping hernia analysis.

^#^Denotes the six residual significant signals following conditional regression analysis at the lead SNP at the locus

Bold font depicts loci which are previously unreported.

### *In silico* annotation of overlap and umbrella cohort analyses

We used FUMA *SNP2GENE* [17] to annotate the overlap and umbrella hernia cohorts. In the overlap hernia cohort, 187 genome-wide significant candidate SNPs were identified by FUMA to be in LD (r^2^ > 0.6) with the lead variant at each of the four loci. No exonic variants were identified, however, six intronic / intergenic variants had predicted deleterious effects and were in high LD with the index variant at each overlap hernia loci, including three at locus 1q41 (*ZC3H11B*) and two at locus 2p16.1 (*EFEMP1*) (S14 Table).

Analysis of the umbrella hernia cohort yielded 877 genome-wide significant candidate SNPs in LD with the lead SNP at the 19 loci. Thirty-eight intergenic or intronic variants were predicted to be functional (S15 Table), and 18 high LD exonic variants were discovered (S16 Table), 15 of which were at the MHC locus (6p22.2). Of these, four variants resulted in substitutions predicted to have damaging (PolyPhen) and deleterious (SIFT) consequences on *BTN2A1*. Of the non-MHC exonic variants, rs17855988 results in a pGly581Arg substitution in *ELN* that is predicted by SIFT [18] with low confidence to have a deleterious consequence on elastin function (S16 Table).

### Multivariable meta-analysis of individual hernia phenotypes

We additionally performed multivariable meta-analysis of the four individual hernia traits in metaUSAT [16] across a total of 57, 418 individual hernia cases and 287,090 matched controls in UK Biobank. metaUSAT enables joint analysis of summary statistics from existing GWAS such that statistical power is augmented more so than for multiple univariable analyses alone. Designed to be robust to the association structure of correlated traits, metaUSAT may provide further insight into a shared genetic architecture for multiple hernia phenotypes.

**Fig 6:**
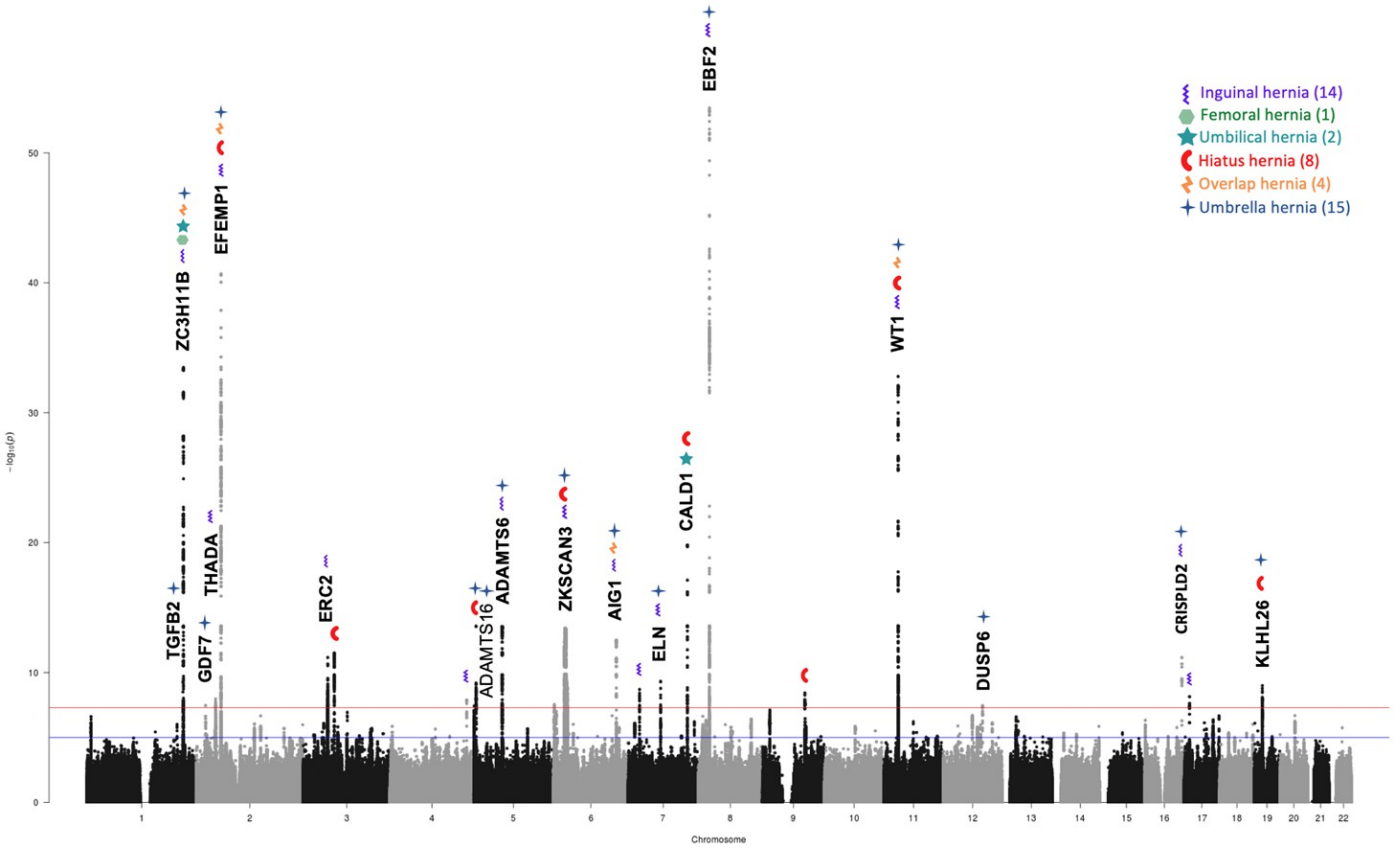
24 loci discovered to confer shared hernia susceptibility after multivariate meta-analysis in 57,418 cases and 287,090 controls in metaUSAT. Each metaUSAT locus is annotated according to whether it was genome-wide significant in the individual, overlap or umbrella analyses.

Twenty-four genome-wide significant susceptibility loci were discovered (3,645 variants) (Table 4) with one-third of loci becoming more significant in metaUSAT than in any of the previous GWAS analyses. Concordance was demonstrated with the umbrella hernia analyses, as of the 19 loci which were significant under umbrella cohort analysis, 15 loci were also discovered under metaUSAT (Fig 6). Evidence for shared susceptibility was further provided at nine loci including 2p21 (*THADA)*, 3p14.3 *(ERC2)*, 3p13 *FOXP1*, 4q34.1 *(HAND-AS1)*, 5p15.33 *(CEP72)*, 7p15.2 *(LOC646588)*, 7q33 *(CALD1)* and 9q22.31 (*BARX1)* of which eight were previously discovered on individual hernia GWAS analyses. Interestingly, 5p15.33 (rs72703080, p_metaUSAT_ = 3.68×10^−8^, (~20kb upstream from CEP72) was an entirely new putative locus which was sub-threshold across all six individual or combined GWAS analyses.

**Table 4:** 24 genome-wide significant loci discovered in the metaUSAT multivariable meta-analysis of inguinal, femoral, umbilical, hiatus hernia in 57,418 cases and 287,090 controls in UK Biobank. Statistically significant signals from the metaUSAT analysis are shown in the left-hand column. The central column shows the association p-values for those SNPs in the six original GWAS analyses, with the direction of effect indicated by a + or–sign. Candidate genes are those selected from the prioritised genes (using the four mapping strategies described previously for all GWAS-discovered loci) or genes in proximity as identified within the UCSC genome browser.

metaUSAT analysis							Candidate Gene	GWAS analyses						
								Four individual hernia (P-value)				Combined hernia (P-value)		Direction^f^
Chr^a^	BP^a^	SNP	A1^b^	A0^c^	T-statistic^d^	P-value^e^		IH	FH	UH	HH	Overlap	Umbrella	
1q41	218521609	rs2799098	G	A	3.53×10^−10^	**5.18×10** ^**−10**^	*TGFB2*	1.10×10^−7^	9.90×10^−1^	9.60×10^−2^	8.70×10^−6^	1.40×10^−4^	**9.30×10** ^**−15**^	-+----
1q41	219754012	rs559230165	C	CT	8.23×10^−33^	**3.28**×**10**^**−34**^	*ZC3H11B*	**1.50×10** ^**−11**^	**2.20×10** ^**−9**^	**1.30×10** ^**−18**^	7.90×10^−1^	**1.70×10** ^**−15**^	**1.90×10** ^**−21**^	------
2p24.1	20878406	rs3072	T	C	2.23×10^−8^	**3.48×10** ^**−8**^	*GDF7*	1.70×10^−2^	9.40×10^−1^	9.00×10^−1^	6.30×10^−8^	3.00×10^−2^	**1.80×10** ^**−8**^	--+---
2p21	43665943	rs76684055	G	A	6.96×10^−9^	**1.07×10** ^**−8**^	*THADA*	**2.80×10** ^**−10**^	9.40×10^−1^	2.30×10^−1^	1.20×10^−1^	8.30×10^−1^	3.80×10^−5^	+--+++
2p16.1	56106928	rs59985551	C	T	1.10×10^−39^	**2.04×10** ^**−41**^	*EFEMP1*	**4.70×10** ^**−40**^	1.00×10^−2^	2.20×10^−2^	**7.20×10** ^**−2**^ ^†^	**8.30×10** ^**−18**^	**2.70×10** ^**−33**^	++++++
3p14.3	56141843	rs7647972	C	G	1.71×10^−11^	**6.97×10** ^**−12**^	*ERC2*	**8.90×10** ^**−12**^	1.20×10^−1^	4.90×10^−1^	1.10×10^−2^	2.70×10^−1^	5.70×10^−2^	+---++
3p13	70951945	rs5007038	A	T	7.78×10^−12^	**3.07×10** ^**−12**^	*FOXP1*	1.80×10^−1^	9.90×10^−3^	9.30×10^−2^	**9.60×10** ^**−12**^	7.30×10^−1^	1.80×10^−7^	+-----
4q34.1	174606591	rs12649191	T	C	1.17×10^−8^	**1.81×10** ^**−8**^	*HAND-AS1*	**6.20×10** ^**−10**^	2.60×10^−1^	1.60×10^−1^	2.90×10^−1^	2.00×10^−3^	2.90×10^−4^	-+-+--
5p15.33	595238	rs72703080	A	G	2.35×10^−8^	**3.68×10** ^**−8**^	*CEP72*	4.40×10^−1^	4.30×10^−1^	5.00×10^−4^	1.70×10^−7^	7.40×10^−1^	7.30×10^−3^	+-+---
5p15.32	4977446	rs42202	A	G	1.44×10^−15^	**2.71×10** ^**−14**^	*ADAMTS16*	8.40×10^−1^	9.40×10^−2^	5.70×10^−1^	**8.00×10−** ^**16**^	8.40×10^−4^	**1.90×10** ^**−11**^	-+-+++
5p15.32	5350637	rs7715383	G	C	1.97×10^−9^	**2.97×10** ^**−9**^	*ADAMTS16*	5.00×10^−5^	7.00×10^−1^	4.80×10^−2^	2.30×10^−7^	1.40×10^−1^	**1.20×10** ^**−12**^	------
5q12.3	64355060	rs370763	T	A	5.98×10^−15^	**2.84×10** ^**−14**^	*ADAMTS6*	**3.30×10** ^**−17**^	4.60×10^−1^	6.00×10^−1^	2.80×10^−1^	7.70×10^−6^	**8.30×10** ^**−12**^	------
6p22.1	27352750	rs71559024	G	A	3.17×10^−14^	**3.64×10** ^**−14**^	*ZKSCAN3*	**2.20×10** ^**−10**^	8.80×10^−1^	3.30×10^−1^	**8.10×10** ^**−8**^ ^†^	2.80×10^−3^	**2.10×10** ^**−17**^	++++++
6q24.2	143676186	rs6570555	A	T	8.89×10^−13^	**3.42×10** ^**−13**^	*AIG1*	**7.80×10** ^**−13**^	4.70×10^−2^	1.80×10^−2^	1.80×10^−1^	**2.20×10** ^**−7**^ ^†^	**1.00×10** ^**−11**^	+-++++
7p15.2	25681464	rs10951081	C	A	1.32×10^−9^	**1.98×10** ^**−9**^	*LOC646588*	**5.30×10** ^**−9**^	9.70×10^−2^	1.30×10^−3^	9.50×10^−1^	6.00×10^−1^	1.40×10^−2^	++--++
7q11.23	73422593	rs76027228	C	T	3.32×10^−10^	**4.83×10** ^**−10**^	*ELN*	**2.40×10** ^**−8**^	5.50×10^−2^	8.40×10^−4^	2.00×10^−2^	7.20×10^−3^	**8.60×10** ^**−12**^	++++++
7q33	134593511	rs4472440	C	G	9.20×10^−20^	**1.54×10** ^**−20**^	*CALD1*	3.10×10^−1^	9.90×10^−1^	**7.10×10** ^**−15**^	**1.30×10** ^**−8**^	1.10×10^−1^	6.10×10^−2^	---+++
8p21.2	25717620	rs6983815	T	A	7.26×10^−52^	**3.47×10** ^**−54**^	*EBF2*	**1.10×10** ^**−54**^	4.10×10^−1^	4.30×10^−1^	4.40×10^−1^	3.10×10^−4^	**1.00×10** ^**−18**^	-+++--
9q22.31	96624645	rs4075733	C	T	2.45×10^−9^	**3.71×10** ^**−9**^	*BARX1*	5.90×10^−1^	1.40×10^−1^	4.60×10^−1^	**1.50×10** ^**−9**^	2.50×10^−1^	3.20×10^−4^	--++-+
11p13	32458278	rs5030123	G	GT GT	3.73×10^−32^	**1.60×10** ^**−33**^	*WT1*	**2.00×10** ^**−19**^	6.30×10^−3^	1.40×10^−1^	**7.70×10** ^**−16**^	**1.20×10** ^**−12**^	**1.50×10** ^**−41**^	++++++
12q21.33	89767237	rs797267	A	G	2.25×10^−8^	**3.51×10** ^**−8**^	*DUSP6*	1.00×10^−2^	5.60×10^−4^	4.20×10^−1^	1.70×10^−6^	3.00×10^−1^	**2.60×10** ^**−9**^	------
16q24.1	84856552	rs4238714	T	C	1.73×10^−11^	**7.05×10** ^**−12**^	*CRISPLD2*	**2.80×10** ^**−13**^	4.50×10^−1^	1.00×10^−1^	6.40×10^−1^	4.70×10–4	**1.60×10** ^**−9**^	------
17p12	12191339	rs12453693	C	T	4.73×10^−9^	**7.22×10** ^**−9**^	-	**3.00×10** ^**−11**^	6.00×10^−1^	8.50×10^−1^	8.10×10^−1^	6.10×10–2	1.20×10^−4^	-+++--
19p13.11	18787981	rs2891698	G	A	6.85×10^−10^	**1.02×10** ^**−9**^	*KLHL26*	6.20×10^−1^	5.50×10^−1^	4.80×10^−1^	**4.00×10** ^**−10**^	1.00×10^−1^	**1.60×10** ^**−8**^	++++++

^a^Based on NCBI Genome Build 37 (hg19).

^b^The reference allele.

^c^The alternate allele.

^d^The metaUSAT test statistic (scalar).

^e^The p-value of association based on the metaUSAT statistic.

^f^The effect size direction in the six BOLT-LMM association analyses (IH, UH, FH, HH, OH, Umbrella) with respect to the reference allele

^g^Genes were selected based on those gene mapped in the six GWAS analyses, and subsequently prioritised based on the existing literature.

Bold p-values are those variants identified by metaUSAT that are genome-wide significant (p <5×10^−8^) in a particular analysis.

^†^Denotes three loci where the reference metaUSAT SNP is not significant in the individual BOLT-LMM hernia analysis, however the locus contains genome-wide significant SNP associations. The following shorthand notations are used: Inguinal Hernia, IH; Femoral Hernia, FH; Umbilical Hernia, UH; Hiatus Hernia, HH; Overlap Hernia, OH; Umbrella Hernia, Umbrella.

### Gene set, pathway and tissue enrichment analysis of combined hernia susceptibility loci

Gene set analysis of overlap hernia susceptibility loci, performed in MAGMA [19], revealed enrichment for gene ontologies for cellular components associated with ‘*Blastoderm segmentation’* (p = 9.93×10^−8^, n = 19 genes) whilst the top curated gene set was ‘Reactome elastic fibre formation’ (p = 1.74×10^−7^, n = 46 genes) (S17 Table). Upon XGR (eXploring Genomic Relations) [20] analysis, two canonical pathways were found to be enriched–‘*Regulation of Telomerase’* (p = 3.80×10^−4^, Z = 4.88, FDR = 1.0×10^−3^, *WT1*) and ‘*Genes encoding structural ECM glycoproteins*’ (p = 3.2×10^−3^, Z = 2.7, FDR = 5.4×10^−3^, *EFEMP1* (S18 Table).

Enrichment for gene ontologies related to the ECM was further substantiated on gene-set analysis of umbrella cohort susceptibility loci, whereby MAGMA gene-set analysis enriched 29 gene sets from MSigDB [21] (S19 Table). Two enriched gene sets were ‘*Negative regulation of cell proliferation in kidney development*’ (p = 5.76×10^−7^) and ‘*Diaphragm development’* (p = 3.00×10^−6^, n = 9 genes).

Strong enrichment for gene ontologies for ‘*Connective tissue development’* was discovered (p = 3.29×10^−8^, n = 46 genes) whilst the top curated gene set was ‘*Elastic fibre formation’* (p = 3.29×10^−8^, n = 46 genes) and the top molecular functions gene ontology was ‘BMP receptor binding’ (3.36×10^−8^, n = 8 genes). Furthermore, other enriched biological process gene sets included *‘Skeletal system development*’ (p = 3.28×10^−7^, n = 498 genes) and *‘Thorax and anterior abdomen determination’* (p = 1.42×10^−6^, n = 5 genes).

Of note, tissue expression analysis in MAGMA [19] revealed Adipose Visceral Omentum to be most enriched whilst Adipose Subcutaneous tissue to be fourth most enriched (p = 1.11×10^−3^). GTEx v8.0 30 general tissue types analysis confirmed this strong enrichment for Adipose tissue (p = 6.31×10^−4^, most enriched) (S7 Fig).

#### Genetic risk score

We also implemented genetic risk score methodology in order to explore a hypothetical correlation between phenotypic severity and genotypic burden. A weighted genetic risk score for surgically managed cases versus non-surgically managed cases for both individual and combined hernia analyses was constructed from the lead independent variants from association analyses. All hernia patients who had undergone surgery had a higher wGRS compared to non-surgically managed hernia patients (Table 5).

**Table 5:** Weighted genetic risk scores in surgically-managed hernia patients versus non-surgically managed hernia patients.

Group	Surgically-managed hernia cases	Non-surgically managed hernia cases	p-value^§^
**Inguinal hernia, N**	18,082	709	
Mean wGRS (S.D.)	3.072 (0.332)	3.013 (0.337)	5.46×10^−6^
**Femoral hernia, N**	774	199	
Mean GRS (S.D.)	0.739 (0.688)	0.597 (0.642)	0.015
**Umbilical hernia, N**	4,749	607	
Mean wGRS (S.D.)	0.653 (0.228)	0.630 (0.229)	2.13×10^−2^
**Hiatus hernia, N**	1,311	30,987	
Mean wGRS (S.D.)	0.464 (0.115)	0.454 (0.115)	4.77×10–3
**Overlap hernia, N**	4,941	278	
Mean wGRS* (S.D.)	1.096 (0.336)	1.036 (0.338)	3.98×10^−3^
**Umbrella hernia, N**	29,857	32,780	
Mean wGRS* (S.D.)	1.366 (0.180)	1.343 (0.180)	4.87×10^−55^

*wGRS: weighted genetic risk score.

^§^Unpaired t-tests were performed for the surgical vs non-surgical wGRS comparisons for all hernia analyses with the exception of femoral hernia. The femoral hernia GRS was not normally distributed as it was based only on one SNP, so a Mann-Whitney U-test was performed.

### Genetic correlations of individual hernia phenotypes

Estimated genetic correlation between individual hernia subtypes was evaluated with LDSC [22] using GWAS summary statistics from individual hernia GWAS analyses. Genetic correlation was greatest between inguinal and femoral hernia subtypes with *r*_*g*_ 0.60, p = 0.011, followed by umbilical and hiatus hernia which yielded *r*_*g*_ of 0.21 with p = 0.0041 (Table 6). Umbilical-inguinal hernia also showed positive correlation with *r*_*g*_ 0.19, p = 0.029. Evidence of genetic correlations was not observed between inguinal-hiatus or femoral-umbilical hernia phenotypes.

**Table 6:** Genetic correlations of individual hernia phenotypes with LDSC.

Umbilical	Femoral	Inguinal	Hiatus	
				**Umbilical**
**-**	-0.01	0.19	0.21	**Genetic Correlation** ^a^
**-**	0.2696	0.0871	0.0722	**SE** ^b^
-	-0.04	2.1901	2.8706	**Z** ^c^
-	0.97	0.029	0.0041	**P-value** ^d^
				**Femoral**
	-	0.60	0.051	**Genetic Correlation** ^a^
	-	0.236	0.1425	**SE** ^b^
	-	2.5438	0.3609	**Z** ^c^
	-	0.011	0.72	**P-value** ^d^
				**Inguinal**
		-	0.048	**Genetic Correlation** ^a^
		-	0.0511	**SE** ^b^
		-	0.9294	**Z** ^c^
		-	0.35	**P-value** ^d^

^a^The genetic correlation estimate (r_g_).

^b^The standard error given by LDSC [22].

^c^Obtained z-score.

^d^The p value given by LDSC [22].

## Discussion

We performed a six-stage genome-wide association study (GWAS) of multiple hernia phenotypes with additional multivariable meta-analysis using metaUSAT in order to characterise the shared genetic underpinnings of common hernia phenotypes. We identified 38 susceptibility loci (11 previously unreported) associated with inguinal, femoral, umbilical or hiatus hernia among an umbrella cohort of 62,637 individuals derived from UK Biobank. Five biologically relevant loci were discovered on individual hernia analyses to confer shared susceptibility to multiple hernia phenotypes including 1q41 (*ZC3H11B*), 2p16.1 (*EFEMP1*), 6p22.1 (MHC region), 7q33 (*CALD1*) and 11p13 (*WT1*). These loci were also prioritised on combined hernia cohort GWAS analyses with the umbrella hernia cohort analysis resulting in discovery of 14 further shared susceptibility loci including 1q41 *(TGFB2)*, 2p24.1 *(GDF7)*, 3q22.2 *(AMOTL2)*, 5p15.32 (*ADAMTS16*), 7q11.23 *(ELN*), 8p21.2 (*EBF2*) and 12q21.33 (*DUSP6*). Multivariable meta-analysis in metaUSAT enabled joint analysis of traits, enabling augmentation of study power and demonstrated strong concordance with that of all previous GWAS analyses with 24 genome-wide significant loci prioritised. metaUSAT highlighted nine additional shared susceptibility loci, of which eight were discovered on individual hernia GWAS analyses, representing potentially important genetic elements of shared hernia biology. These included 2p21 (*THADA)*, 3p14.3 (*ERC2)*, 3p13 (*FOXP1)*, 4q34.1 (*HAND-AS1)*, 9q22.31 (*BARX1)* and 17p12.

To our knowledge, this study also represents the first GWAS of hiatus hernia in which we identified eight susceptibility loci of which four loci (2p16.1 *(EFEMP1)*, 6p22.2 *(BTN2A1)*, 7q33 *(CALD1)*, 11p13 *(WT1)* are known to correlate with abdominal hernia. This provides further proof of principle with regards to the shared genetic underpinnings of a common hernia phenotype. The four remaining susceptibility loci for hiatus hernia have previously not been reported.

It has been postulated that dysregulation of elastic tissue biology mediated via matrix metalloproteinases (MMPs) is central to the pathophysiology of hernia development. Jorgenson and colleagues previously identified 4 inguinal hernia susceptibility loci purported to result in reduced MMP activity: *WT1*, *EFEMP1*, *EBF2* and *ADAMTS6* [13]. Recently, Wei et al. replicated these associations and further implicated *AIG1* and *CALD1*, which were identified as biologically relevant genes in their individual GWAS’s of hernia phenotypes [15]. We have extended these results to focus on the shared biology of abdominal wall hernias. Our study provides further evidence that these loci each impart susceptibility to multiple hernia phenotypes, supported by the observation that these demonstrated some of the strongest associations in the combined cohorts, while also being prioritised in the multivariable meta-analysis.

### Genes of interest

The 1q41 locus (*ZC3H11B*) was strongly associated across three individual hernia phenotypes, the combined hernia cohorts, and importantly was also prioritised in metaUSAT meta-analysis. *ZC3H11B*, a zinc finger CCH domain-containing protein, was previously identified by Wei *et al*. [15] in association with inguinal, femoral, umbilical and ventral hernia. *ZC3H11B* has also been associated with myopia endophenotypes, including axial length, refractive error, and corneal astigmatism [23]. It is thought that accelerated connective tissue remodelling of the posterior sclera leads to axial elongation, a key feature of myopia [24], thereby implicating ZC3H11B in a number of suspected connective tissue diseases. Intriguingly, at least two Marfan-like syndromes have been described with co-existing myopia and hernia [25, 26].

Locus 2p16.1 (*EFEMP1*) imparted susceptibility to inguinal and hiatus hernia phenotypes, was significantly associated in both overlap and umbrella cohort analyses, and was also identified in metaUSAT meta-analysis. *EFEMP1* encodes fibulin-3, a secreted extracellular matrix glycoprotein, which has been shown to downregulate matrix metalloproteinases (MMPs) 2 and 3, whilst simultaneously upregulating tissue inhibitor of metalloproteinase-3 [27]. As well as collagen, fibulin-3 binds tropoelastin [28], the monomeric unit of elastin fibres. *EFEMP1* knockout mice show depleted elastic fibres within fascia and invariably develop inguinal hernia, adding further strength to the evidence for its importance in AWH pathophysiology [29].

True pleiotropy is further substantiated by the finding that *EFEMP1* has previously been identified by our group as a candidate gene conferring susceptibility to carpal tunnel syndrome [30] and varicose veins [31], disorders also thought to be underpinned by opposing impairments in extracellular matrix homeostasis. Additionally, *EFEMP1* has recently been implicated in conferring susceptibility to pelvic organ prolapse [32] and intriguingly is also associated with anthropometric measures of height [33] and abdominal circumference [27], which have also been associated with *ZC3H11B* [34].

Additionally, two signals in proximity to *ADAMTS16* were discovered as genome-wide significant in metaUSAT analysis and in the umbrella cohort GWAS analysis. The ADAMTS family are a group of metalloendopeptidases, related to MMPs, serving to synthesise collagen from procollagen [27]. Variants in *ADAMTS16* have been associated with urinary incontinence [35], a manifestation of pelvic floor dysfunction, which have been shown independently to lead to a higher prevalence of hiatus and inguinal hernia [36]. Several mutations have been described in the other 18 ADAMTS superfamily genes, which result in distinct human genetic disorders [37]. For example, mutations in *ADAMTS2* are responsible for dermatosparactic type Ehlers-Danlos Syndrome (type VIIC) [38], typified by extreme skin fragility, joint laxity, and umbilical hernia. *ADAMTS4* shows significant aggrecanase activity and is implicated in articular cartilage degradation and arthritis [39], and *ADAMTS4* mRNA and protein have been found to be highly expressed in herniated lumbar intervertebral discs [40].

metaUSAT met-analysis yielded a further candidate gene at 1q41 *(TGFB2*) which was also the most statistically significant locus in the umbrella hernia analysis. *TGFB2* encodes the protein Transforming Growth Factor β2 (TGF β2) which is observed to be upregulated in Marfan syndrome, Loeys-Dietz syndrome, and cutis laxa, which are associated with aneurysmal changes with histological features including smooth muscle cell apoptosis [41]. Furthermore, *TGFB2* haploinsufficiency pathologically activates the TGF-β signalling pathway [41], leading to Loeys-Dietz syndrome type 4 [42], which is characterised by arterial vasculopathy (arterial aneurysms, dissection and tortuosity), and other widespread connective tissue pathology, including hernia [43]. Like *ZC3H11B*, *TGFB2* has previously been implicated in ophthalmic pathology including glaucoma endophenotypes, with roles in intraocular pressure [44], central corneal thickness [45], as well as FEV1/FVC ratio [46] and severe chronic obstructive pulmonary diseaseO [47], of which the latter has also been suggested as an independent risk factor for hernia pathology and severity [48, 49].

Among the further candidates discovered on multivariable meta-analysis, *CEP72* at 5p15.33 encodes a centriolar satellite protein necessary for regulating microtubule-organising activity and centrosome integrity [50]. Using comparative genomic hybridisation, Choi *et al*. discovered copy number increases at 5p15.33 in patients with ruptured intracranial aneurysms [51]. The *CEP72* region has also been implicated in a genome-wide meta-analysis of Barrett’s oesophagus and oesophageal adenocarcinoma [52], for which hiatus hernia is a major risk factor [53]. Indeed, the size of a hiatus hernia is significantly associated with progression of Barrett’s oesophagus to high-grade dysplasia or malignancy [54]. To this end, a tangible and biologically plausible contributor to shared hernia risk has been identified through multivariate meta-analysis.

*GDF7* was discovered to associate with hernia in the umbrella and metaUSAT analyses, with the lead variant rs3072 demonstrating strong functionality as a robust eQTL for *GDF7* in GTEx aorta tissue (P_eQTL_ = 5.4×10^−9^). *GDF7* encodes BMP12, part of the bone morphogenetic protein pathway, and is heavily implicated in Barrett’s oesophagus [55] with several studies identifying polymorphisms in the *TBX-GDF7* genomic region [56, 57]. *GDF7* has been identified through GWAS to associate with eight traits, three of which are characterised by connective and elastic tissue dysfunction: pelvic organ prolapse [32], abdominal aortic aneurysm [58], and diverticular disease [59]. The T allele of rs7255 (which is in high LD with lead SNP rs3072) was also found to confer risk of Barrett’s oesophagus in the GWAS by Gharahkhani *et al*. [52].

The umbrella analysis further revealed association at 3q22.2 locus which was not identified in any other analyses presented. Interestingly, *de novo* deletions at 3q22.1 result in a syndromic presentation of bilateral inguinal hernia [60] and an interstitial deletion of 3q23 has been described to result in BPES syndrome, characterised by diaphragmatic hernia [61]. This region on the long arm of chromosome 3 may therefore be of considerable interest in multiple hernia pathobiology.

### Genetic risk scoring

Our simple weighted genetic risk score correlated with disease severity, with patients undergoing surgery having a higher genetic burden than those managed non-surgically across all individual hernia subtypes and overlap analyses. These data provide an important proof-of-principle of genetic risk scoring in personalising risk in this highly prevalent disease. Further work to validate the risk score in an independent cohort is required.

### Genetic correlations of individual hernia phenotypes

We found strong positive genetic correlations between femoral and inguinal hernia, with *r*_*g*_ 0.60, further supporting the notion of shared genetic architecture. It is possible however, that due to the small femoral hernia sample size, these findings may have been spurious. However, robust correlations were also observed between umbilical-hiatus and umbilical-inguinal hernia, of which the former was most statistically significant (*r*_*g*_ 0.21, P = 0.0041). Given the large sample sizes of the inguinal and hiatus hernia cohorts, it is interesting that a correlation was not observed between these phenotypes. This may reflect the fact that hiatus hernia occurs through the diaphragm, which is derived embryologically from the septum transversum and not somitic and lateral plate mesoderm [62].

### Limitations

Our primary aim was to identify shared susceptibility across multiple hernia phenotypes which is made possible in a large Biobank-scaled cohort. The lack of a replication cohort for these results is a clear limitation, however, this was somewhat mitigated by the use of stringent quality control and case definitions as well as the implementation of four distinct analytic strategies. Secondly, the use of unselected biobank data inevitably results in imbalance between the phenotypes. This means that hiatus and inguinal hernia, which are substantially more common in the UK population, were overrepresented in our dataset as they accounted for approximately 90% of the total cohort and were therefore more powered in the joint analyses. A larger cohort with greater balance across all four hernia phenotypes may prove useful in further defining shared genetic susceptibility loci as well as uncovering new associations. Finally, we acknowledge the limitations of restricting the GWAS analyses to a cohort of white British ancestry, and that the genetic loci for hernia susceptibility identified in this study may not be applicable to individuals of other ancestries.

## Conclusions

In conclusion, the distinct analytic approaches to examine the shared genetic architecture of the four hernia subtypes allowed us to discover new insights into the biology of abdominal wall hernias. We discovered new genetic associations that were not found on traditional single-trait association analyses. By segregating the four hernia cohorts in UK Biobank to avoid overlap, we can have confidence in the validity of several loci that were discovered across multiple hernia phenotypes. This is the case for the twelve loci that demonstrated the greatest degree of overlap across the different analyses, and the resulting clustering of many of these loci across functionally related ontologies. Furthermore, the enrichment of biological pathways previously implicated in hernia pathobiology provides further compelling evidence to support the veracity of these loci, and for a shared genetic susceptibility to hernia.

## Methods

### Overview

We performed four individual genome-wide association studies (GWAS) of hernia subtypes (inguinal, femoral, umbilical and hiatus hernia) of 488,377 UK participants, aged 40–69 years at the time of recruitment, who provided written consent to be prospectively enrolled into the UK Biobank multicentre cohort from 2006–2010. The full characteristics of the UK Biobank cohort are described in full elsewhere [63, 64].

### Ethics statement

UK Biobank obtained ethical approval from the North West Multi-Centre Research Ethics Committee (MREC) (11/NW/0382). This study was conducted under UK Biobank project ID 22572.

We looked for evidence of shared genetic underpinnings between the four distinct hernia phenotypes with a further GWAS analyses of participants with multiple hernia phenotypes, as well as a sixth combined cohort of participants with individual or multiple hernia phenotypes. Additionally, we undertook multivariable meta-analysis to characterise shared susceptibility loci, by aggregating information across the four correlated traits.

### Cohort definitions

The UK Biobank population was divided into three hernia cohorts as described below. Each was matched 1:5 to non-hernia controls.

i **Individual hernia cohort**. This comprised of participants who had diagnostic and/or operative codes for just one of four hernia phenotypes studied. That is, either inguinal, femoral, umbilical or hiatus hernia. Participants having more than one hernia type were excluded.
ii **Overlap hernia cohort**. This cohort consisted of participants with at least two of the four hernia phenotypes studied. Participants affected by a single hernia phenotype were excluded.
iii **Umbrella hernia cohort**. This encompassed all participants with any of the hernia phenotypes studied. As such, this cohort was comprised of cohorts (i) plus (ii).

The full list of diagnostic and operative codes used are shown in S20 Table.

### Genotyping

UK Biobank participants were genotyped sequentially, initially with the Affymetrix BiLeve Axiom array (805,426 directly genotyped variants) and Affymetrix UK Biobank Axiom arrays (825,927 genotyped variants), which share 95% marker content. The present study is based on the third release of the UK Biobank cohort (July 2017), which contained the complete set of genotypes for the 488,377 participants.

### Quality control

Quality control (QC) was performed using PLINK v1.919 and R v3.3.1. The full details of quality control (QC) has been previously described [30]. Briefly, SNPs with low call rates (<98%) were initially excluded. Samples with heterozygosity >3.5 SD from the mean, discordant sex information, or who were not of white British ancestry (ethnic outliers) were excluded. A linear mixed model implemented in BOLT-LMM enabled the inclusion of related participants. SNP-level QC was further performed based on deviations from Hardy-Weinberg equilibrium (p < 10^−4^), minor allele frequency (MAF) <0.01, as well as on visual inspection of autosomal heterozygosity against call rate.

### Imputation

Phasing and imputation of UK Biobank was performed centrally using a 1000 Genomes Consortium Phase 3 reference panel in SHAPEIT3, and has been detailed elsewhere [65].

### Association analyses in BOLT-LMM

In the UK Biobank, GWAS was performed across 8,944,547 imputed SNPs (547,011 directly genotyped (MAF ≥ 0.01) and 8,397,536 imputed SNPs (MAF ≥ 0.01, INFO score ≥ 0.90) using a linear mixed non-infinitesimal model implemented in BOLT-LMM v2.323 [66]. The reference human genome assembly used was GRCh37 (hg19) and linkage disequilibrium scores were obtained from participants of European-ancestry extracted from the BOLT-LMM package. Covariates included in the model were genetic sex and genotyping platform. Association testing was implemented by linear regression assuming an additive allelic effect using imputed allelic dosages. Conditional regression analysis was performed in BOLT-LMM for the top signal at each significant locus (except the MHC region), and repeated until no further residual significant signals remained.

### Functional annotation of SNPs

Annotation of associated SNPs was performed in FUMA *SNP2GENE* v1.3.6 [17], using summary statistics from the UK Biobank discovery cohort and default settings. As such, genomic location and effect/non-effect allele were used to collate functional annotation data from established genetic annotation databases, including ANNOVAR [67], RegulomeDB [68], CADD [69], and 15-core chromatin state categories [70]. Exonic SNPs were investigated further using gnomAD and Ensembl genome browsers to uncover putative functionality [71].

### Candidate gene mapping

Four gene mapping approaches were implemented: FUMA positional mapping [17], eQTL mapping [17], summary-based mendelian randomisation [72] (SMR), and MAGMA genome-wide gene association analysis [19] (GWGAS), with strict Bonferroni correction to account for multiple testing (p < 2.67×10^−6^).

### Multivariable meta-analysis in metaUSAT

The metaUSAT [16] multivariable method was used as an auxiliary meta-analysis method to further characterise potential regions of shared hernia susceptibility between the four individual hernia traits. metaUSAT performs a unified association test for each SNP, using the estimated correlation matrix to test association, across several trait summary statistics. metaUSAT is data-adaptive and was established to be robust to the association structure of correlated traits (less affected by the true (unknown) association structure) and is not dependent on individual‐level data [16] Unlike other multi-trait meta-analysis approaches, metaUSAT does not assume homogeneity of effects across traits. metaUSAT outputs an approximate asymptomatic P-value for the meta-analysis association and has been shown to maintain a low type I error in simulation experiments [16]. The metaUSAT meta-analysis was performed across the four individual hernia cohorts (total 57,418 cases and 287,090 controls) and 8,896,286 SNPs. The genome-wide significant threshold for the metaUSAT association was set a p < 5×10^−8^.

### Gene set, tissue-specific, and pathway enrichment analysis

Gene-set analysis were performed in MAGMA v1.07 [19] across 15,496 gene sets obtained from MSigDB v8.0 [21] with p < 3.23×10^−6^ deemed significant. Enrichment of the overlap between GWAS variants and those reported in previous GWAS within the NIH GWAS Catalog were also examined [73], with enrichment P-values for the proportion of overlap in the genes determined. Tissue-specific analysis was also performed in MAGMA v1.07 to assess gene expression from 30 tissue types in GTEx v8 [17].

The gene set and tissue expression analyses described above were then repeated within FUMA *GENE2FUNC* v1.3.5d [17], to specifically examine the functionality of genes prioritised directly from the four candidate gene mapping approaches. Gene set enrichment analyses of the gene sets within MSigDB v8.0 [21] were tested, and gene property and tissue enrichment analyses within GTEx [17] consortium tissue was also performed distinctly for the prioritised hernia associated genes.

Using eXploring Genomic Relations [20] (XGR) software, pathway enrichment analysis of the prioritised genes was performed to highlight canonical pathways that were enriched. A hypergeometric distribution test was performed and adjusted FDR < 0.05 used to highlight prioritised gene sets.

### Genetic risk score

Weighted genetic risk scores (wGRS), based on the lead independent variants at each genome-wide significant locus, were constructed for each of the six GWAS. For each of the Individual cohorts, overlap and umbrella cohort summary statistics, wGRS were compared between all cases managed surgically and those that were not surgically managed. Surgical cases were defined as those with OPCS (*Office of Population Censuses and Surveys Classification of Interventions and Procedures*) or self-reported operative codes.

The following formula was implemented:

$$wGRS=\sum_{i=1}^{n}W_{i}X_{i}$$

where *i* is the lead SNP at each genomic risk locus, n is the total number of lead SNPs in the GWAS, *Wi* is the weighting for each of the SNPs (the natural logarithm of the odds ratio for each effect allele), and *Xi* is the number of effect alleles each individual possesses for each SNP. Each individual’s risk allele was used to compute a SNP dosage (QCTOOL v2). wGRS calculations and statistical tests between the different subgroups was performed in R v3.3.

### Genetic correlations of individual hernia phenotypes

To investigate potential genetic correlation between the four individual hernia subtypes, linkage disequilibrium score regression (LDSC) was performed between the four individual hernia GWAS analyses. This method evaluates genetic correlation between traits based on a fitted linear model of Z-scores calculated using GWAS summary statistics [22]. For polygenic traits with shared genetic architecture, SNPs with high LD would, on average, be expected to have higher Z-scores than those with low LD. As this was an exploratory analysis, P-values were not corrected for multiple testing.

## Supporting information

S1 Table Sex distribution across all four individual hernia cohorts, overlap and umbrella hernia cohorts. Numbers of total and sex-specific cases and controls are detailed for each of the six cohorts analysed.
(PDF)

S2 Table Twenty-eight signals at 24 loci associated with inguinal hernia in 18,791 cases and 93,955 controls in UK Biobank. ^a^Based on NCBI Genome Build 37 (hg19).^b^The effect allele. ^c^The non-effect allele. ^d^The effect allele frequency. ^e^The SNP INFO score for imputed SNPs; G = genotyped SNP. ^f^The genes prioritised at these loci based on positional mapping, eQTL mapping, MAGMA gene mapping and summary-based mendelian randomisation (see Methods). ^#^Denotes the four residual significant signals following conditional regression analysis at the lead SNP at the locus. Bold loci are those that have not been previously reported.
(PDF)

S3 Table One locus significantly associated with femoral hernia in 973 cases and 4,865 controls in UK Biobank. ^a^Based on NCBI Genome Build 37 (hg19). ^b^The effect allele. ^c^The non-effect allele. ^d^The effect allele frequency. ^e^The SNP INFO score for imputed SNPs; G = genotyped SNP. ^f^ No genes were prioritised at this locus based on positional mapping, eQTL mapping, MAGMA gene mapping and summary-based mendelian randomisation (see Methods).
(PDF)

S4 Table Five loci significantly associated with umbilical hernia in 5,356 cases and 26,780 controls in UK Biobank. ^a^Based on NCBI Genome Build 37 (hg19). ^b^The effect allele. ^c^The non-effect allele. ^d^The effect allele frequency. ^e^The SNP INFO score for imputed SNPs; G = genotyped SNP. ^f^One gene was prioritised at these loci based on positional mapping and MAGMA gene mapping (see Methods). Bold loci are those that have not been previously reported.
(PDF)

S5 Table Eight loci significantly associated with hiatus hernia in 32,298 cases and 161,490 controls in UK Biobank. ^a^Based on NCBI Genome Build 37 (hg19). ^b^The effect allele. ^c^The non-effect allele. ^d^The effect allele frequency. ^e^The SNP INFO score for imputed SNPs; G = genotyped SNP. ^f^The 15 genes prioritised at these loci based on positional mapping, eQTL mapping and MAGMA gene mapping (see Methods). Bold loci are those that have not been previously reported.
(PDF)

S6 Table Individual hernia associated exonic variants. 14 genome-wide significant exonic SNPs associated with inguinal, umbilical and hiatus hernia that were identified by FUMA SNP2GENE. All exonic SNPs are in high linkage with the index SNP at each locus (r2 > 0.6). Non-synonymous missense SNPs predicted to be damaging and deleterious by PolyPhen and SIFT are highlighted in blue.
(PDF)

S7 Table Predicted functional intronic and intergenic variants associated with the four individual hernia phenotypes. 138 genome-wide significant intronic and intergenic variants predicted to be deleterious according to a CADD ≥ 12.37, and associated with inguinal, femoral, umbilical and hiatus hernia as identified by FUMA SNP2GENE. Functional variants with a RegulomeDB score of 2b or less are highlighted in blue.
(PDF)

S8 Table Genome-wide gene-based association analysis for inguinal hernia in MAGMA. 67 protein-coding genes met the threshold for genome-wide significance (P < 2.64x10-6, 0.05/18,917) in this analysis. 59 of the 67 genes lay within the FUMA-defined loci borders and are highlighted in red; genes are arranged in descending order according to the P-value of the MAGMA association.
(PDF)

S9 Table Summary-based Mendelian Randomisation (SMR) for inguinal hernia using eQTL data from GTEx v7. The three probes (genes) that met the Bonferroni-corrected significance threshold P_SMR_ < 1.12×10^−4^ (0.05/4,455) and passed the HEIDI test (P_HEIDI_ ≥ 8.33x10^-3^) (0.05/6)) are shown. eQTL tissues tested were for both GTEx v7 Skeletal muscle and Cells Transformed Fibroblast, however only three probes from skeletal muscle tissue met the HEIDI enrichment threshold. All three were mapped to within the realms of the FUMA-defined susceptibility loci. ^a^Probe ID. ^b^Probe chromosome. ^c^Gene name. ^d^SNP name. ^e^Allele 1. ^f^Allele 2. ^g^Frequency of Allele 1 in the study population. ^h^Effect size of the allele ^i^Standard error of the effect size ^j^GWAS P-value. ^k^eQTL P-value. ^l^SMR P-value. ^m^HEIDI P-value. Probes mapped to within the realms of the FUMA-defined susceptibility loci are highlighted in red.
(PDF)

S10 Table Genes mapped to the inguinal hernia-associated loci using the four mapping strategies. 101 unique genes (169 total) were mapped to 21 of 24 inguinal hernia susceptibility loci by one or more gene mapping strategies. 53 genes were mapped via positional mapping, 42 genes were mapped via eQTL mapping, 64 genes were mapped using MAGMA and 3 genes were mapped using summary-based mendelian randomisation. Overlap between the four different mapping strategies is shown (and highlighted in pink).
(PDF)

S11 Table Genome-wide gene-based association analysis for umbilical hernia in MAGMA. Three protein-coding genes met the threshold for genome-wide significance (p<2.64x10-6, 0.05/18,916) in this analysis. The one gene that lays within the realms of the genome-wide significant susceptibility loci and are highlighted in red.
(PDF)

S12 Table Genome-wide gene-based association analysis for hiatus hernia in MAGMA. 26 protein-coding genes met the threshold for genome-wide significance (p<2.64x10-6, 0.05/18,918) in this analysis. 11 of the 26 genes lay within the realms of the FUMA-defined genome-wide significant susceptibility loci and are highlighted in red.
(PDF)

S13 Table Genes mapped to the hiatus hernia-associated loci using the four mapping strategies. 15 unique genes (20 total) were mapped to 5 of 8 hiatus hernia susceptibility loci by one or more gene mapping strategies. 5 genes were mapped via positional mapping, 4 genes were mapped via eQTL mapping, 11 genes were mapped using MAGMA and no genes were mapped using summary-based mendelian randomisation. Overlap between the four different mapping strategies is shown (and highlighted in pink).
(PDF)

S14 Table Predicted functional intronic and intergenic variants associated with overlap hernia. Six genome-wide significant intronic and intergenic variants predicted to be deleterious according to a CADD ≥ 12.37 and associated with overlap hernia as identified by FUMA SNP2GENE.
(PDF)

S15 Table Predicted functional intronic and intergenic variants associated with umbrella hernia. 38 genome-wide significant intronic and intergenic variants predicted to be deleterious according to a CADD ≥ 12.37, and associated with umbrella hernia as identified by FUMA SNP2GENE. Functional variants with a RegulomeDB score of 2b or less are highlighted in blue.
(PDF)

S16 Table Umbrella hernia associated exonic variants. 18 genome-wide significant exonic SNPs associated with umbrella hernia that were identified by FUMA SNP2GENE. All exonic SNPs are in high linkage with the index SNP at each locus (r2 > 0.6). Non-synonymous missense SNPs predicted to be damaging and deleterious by PolyPhen and SIFT are highlighted in blue.
(PDF)

S17 Table Enriched gene sets from the genome-wide gene-based enrichment analysis of overlap hernia in MAGMA v1.07. The convergence of 15,496 gene sets (15,381from MSigDB v7.0) were tested. A Bonferroni-corrected threshold of P < 3.23×10–6 (0.05/15,496) was set, resulting in 21 significant Gene Ontology (GO) gene sets and three curated gene sets. This analysis was performed using the SNP2GENE tool in FUMA.
(PDF)

S18 Table Gene-based enrichment analysis for overlap hernia associated genes in eXploring Genomic Relations (XGR). (PDF)

S19 Table Enriched gene sets from the genome-wide gene-based enrichment analysis of umbrella hernia in MAGMA v1.07. The convergence of 15,496 gene sets (15,381from MSigDB v7.0) were tested. A Bonferroni-corrected threshold of P < 3.23×10–6 (0.05/15,496) was set, resulting in two significant Gene Ontology (GO) gene sets. This analysis was performed using the SNP2GENE tool in FUMA.
(PDF)

S20 Table Phenotype codes used for four individual hernia case definitions. Hernia cases were defined if they had a diagnostic (ICD-10 or self-report) and/or operative (OPCS4 or self-report) code for either inguinal, femoral, umbilical or hiatus hernia. Overlapping hernia cases were removed to construct four cohorts of individual hernia cases.
(PDF)

S1 Fig Manhattan plots for the four individual hernia analyses in UK Biobank. Manhattan plots are annotated with the gene names of loci that demonstrate shared susceptibility across two or more individual analyses. The 6q24.2 (*AIG1*) locus is plotted for inguinal hernia because it shows shared susceptibility with the overlap hernia analysis. *ZC3H11B* is shown as a putative gene at 1q41 for femoral hernia as it was mapped in the joint analysis in metaUSAT.
(PDF)

S2 Fig Quantile-quantile (Q-Q) plots for all four individual hernia analyses. A: Inguinal hernia analysis. B: Femoral hernia analysis. C: Umbilical hernia analysis. D: Hiatus hernia analysis. The λ_GC_ demonstrated nominal inflation levels across the four association analyses, ranging from 1.00–1.20 (λ_GC_-femoral: 1.00; λ_GC_-umbilical: 1.05; λ_GC_-inguinal: 1.15; λ_GC_-hiatus: 1.20), however the LDSC intercept range of 1.00–1.03 (Femoral: 1.00; Umbilical: 1.01; Inguinal: 1.02; Hiatus: 1.03) and an attenuation ratio of 0.08–0.19 (Umbilical: 0.08; Inguinal: 0.11; Hiatus: 0.13; Femoral: 0.19) is fully in keeping with the effects of polygenicity and large sample size.
(PDF)

S3 Fig Regional Locus Zoom plots for all four Individual hernia associated signals. LocusZoom plots of the 28 inguinal, 1 femoral, 5 umbilical and 8 hiatus hernia independent genome-wide significant associated signals. Plots are ordered by chromosome number and genomic position. SNP position is shown on the x-axis, and strength of association on the y-axis (-log10 P-value). The linkage disequilibrium (LD) relationship between the lead SNP and the surrounding SNPs is indicated by the r2 legend. In the lower panel of each figure, genes within 500kb of the index SNP are shown. The position on each chromosome is shown in relation to Human Genome build hg19.
(PDF)

S4 Fig Quantile-quantile (Q-Q) plots of A) all overlap hernia associated signals and B) all umbrella hernia associated signals. Across both overlap and umbrella hernia analyses, the λ_GC_ was 1.05 and 1.20, respectively, with an LDSC intercept of 1.01 and 1.03 and an attenuation ratio of 0.15 and 0.10.
(PDF)

S5 Fig Regional Locus Zoom plots of all overlap hernia associated signals. LocusZoom plots of the six independent genome-wide significant SNPs at the four overlap hernia associated susceptibility loci. Plots are ordered by chromosome number and genomic position. SNP position is shown on the x-axis, and strength of association on the y-axis (-log10 P-value). The linkage disequilibrium (LD) relationship between the lead SNP and the surrounding SNPs is indicated by the r2 legend. In the lower panel of each figure, genes within 500kb of the index SNP are shown. The position on each chromosome is shown in relation to Human Genome build hg19.
(PDF)

S6 Fig Regional Locus Zoom plots of all umbrella hernia associated signals. LocusZoom plots of the 25 independent genome-wide significant SNPs at the 19 hernia-associated susceptibility loci. Plots are ordered by chromosome number and genomic position. SNP position is shown on the x-axis, and strength of association on the y-axis (-log10 P-value). The linkage disequilibrium (LD) relationship between the lead SNP and the surrounding SNPs is indicated by the r2 legend. In the lower panel of each figure, genes within 500kb of the index SNP are shown. The position on each chromosome is shown in relation to Human Genome build hg19.
(PDF)

S7 Fig MAGMA tissue expression analysis of umbrella hernia. MAGMA Tissue Expression Analysis of the umbrella hernia GWAS-summary data, implemented in FUMA in A) 30 general and B) 54 specific tissue types. This analysis tests the relationship between highly-expressed genes in a specific tissue and the genetic associations from the GWAS. Gene-property analysis is performed using average expression of genes per tissue type as a gene covariate. Gene expression values are log2-transformed average RPKM (Read Per Kilobase Per Million) per tissue type after winsorization at 50, and are based on GTEx v8 RNA-Seq data across 54 specific tissue types and 30 general tissue types. The dotted line indicates the Bonferroni-corrected α level, and the tissues that meet this significance threshold are highlighted in red.
(PDF)

## References

[1] Primatesta P, Goldacre MJ. Inguinal hernia repair: incidence of elective and emergency surgery, readmission and mortality. Int J Epidemiol. 1996 Aug;25(4):835–9. doi: 10.1093/ije/25.4.835 8921464

[2] Kingsnorth A, LeBlanc K. Hernias: inguinal and incisional. Lancet. 2003 Nov 8;362(9395):1561–71. doi: 10.1016/S0140-6736(03)14746-0 14615114

[3] GBD 2015 Mortality and Causes of Death Collaborators. Global, regional, and national life expectancy, all-cause mortality, and cause-specific mortality for 249 causes of death, 1980–2015: a systematic analysis for the Global Burden of Disease Study 2015. Lancet. 2016 Oct 8;388(10053):1459–1544. doi: 10.1016/S0140-6736(16)31012-1 27733281 PMC5388903

[4] Simons MP, Aufenacker T, Bay-Nielsen M, Bouillot JL, Campanelli G, Conze J, et al. European Hernia Society guidelines on the treatment of inguinal hernia in adult patients. Hernia. 2009 Aug;13(4):343–403. doi: 10.1007/s10029-009-0529-7 19636493 PMC2719730

[5] Poelman MM, van den Heuvel B, Deelder JD, Abis GS, Beudeker N, Bittner RR, et al. EAES Consensus Development Conference on endoscopic repair of groin hernias. Surg Endosc. 2013 Oct;27(10):3505–19. doi: 10.1007/s00464-013-3001-9 23708718

[6] Dahlstrand U, Wollert S, Nordin P, Sandblom G, Gunnarsson U. Emergency femoral hernia repair: a study based on a national register. Ann Surg. 2009 Apr;249(4):672–6. doi: 10.1097/SLA.0b013e31819ed943 19300219

[7] Nilsson H, Stylianidis G, Haapamäki M, Nilsson E, Nordin P. Mortality after groin hernia surgery. Ann Surg. 2007 Apr;245(4):656–60. doi: 10.1097/01.sla.0000251364.32698.4b 17414617 PMC1877035

[8] Burcharth J, Pommergaard HC, Rosenberg J. The inheritance of groin hernia: a systematic review. Hernia. 2013 Apr;17(2):183–9. doi: 10.1007/s10029-013-1060-4 23423330

[9] Zöller B, Ji J, Sundquist J, Sundquist K. Shared and nonshared familial susceptibility to surgically treated inguinal hernia, femoral hernia, incisional hernia, epigastric hernia, and umbilical hernia. J Am Coll Surg. 2013 Aug;217(2):289–99.e1. doi: 10.1016/j.jamcollsurg.2013.04.020 23870221

[10] Kielty CM. Elastic fibres in health and disease. Expert Rev Mol Med. 2006 Aug 8;8(19):1–23. doi: 10.1017/S146239940600007X 16893474

[11] Henriksen NA, Yadete DH, Sorensen LT, Agren MS, Jorgensen LN. Connective tissue alteration in abdominal wall hernia. Br J Surg. 2011 Feb;98(2):210–9. doi: 10.1002/bjs.7339 21104706

[12] Liem MS, van der Graaf Y, Beemer FA, van Vroonhoven TJ. Increased risk for inguinal hernia in patients with Ehlers-Danlos syndrome. Surgery. 1997 Jul;122(1):114–5. doi: 10.1016/s0039-6060(97)90273-7 9225924

[13] Jorgenson E, Makki N, Shen L, Chen DC, Tian C, Eckalbar WL, et al. A genome-wide association study identifies four novel susceptibility loci underlying inguinal hernia. Nat Commun. 2015 Dec 21;6:10130. doi: 10.1038/ncomms10130 26686553 PMC4703831

[14] Hikino K, Koido M, Tomizuka K, Liu X, Momozawa Y, Morisaki T, et al. Susceptibility loci and polygenic architecture highlight population specific and common genetic features in inguinal hernias: genetics in inguinal hernias. EBioMedicine. 2021 Aug;70:103532. doi: 10.1016/j.ebiom.2021.103532 34392144 PMC8374389

[15] Wei J, Attaar M, Shi Z, Na R, Resurreccion WK, Haggerty SP, et al. Identification of fifty-seven novel loci for abdominal wall hernia development and their biological and clinical implications: results from the UK Biobank. Hernia. 2021 Aug 11. doi: 10.1007/s10029-021-02450-4 34382107

[16] Ray D, Boehnke M. Methods for meta-analysis of multiple traits using GWAS summary statistics. Genet Epidemiol. 2018 Mar;42(2):134–145. doi: 10.1002/gepi.22105 29226385 PMC5811402

[17] Watanabe K, Taskesen E, Van Bochoven A, Posthuma D. Functional mapping and annotation of genetic associations with FUMA. Nat Commun 2017; 8: 1–10. 29184056 10.1038/s41467-017-01261-5 PMC5705698

[18] Ng PC, Henikoff S. SIFT: Predicting amino acid changes that affect protein function. Nucleic Acids Res. 2003 Jul 1;31(13):3812–4. doi: 10.1093/nar/gkg509 12824425 PMC168916

[19] de Leeuw CA, Mooij JM, Heskes T, Posthuma D. MAGMA: Generalized Gene-Set Analysis of GWAS Data. PLoS Comput Biol 2015; 11: 1–20. doi: 10.1371/journal.pcbi.1004219 25885710 PMC4401657

[20] Fang H, Knezevic B, Burnham KL, Knight JC. XGR software for enhanced interpretation of genomic summary data, illustrated by application to immunological traits. Genome Med. 2016 Dec 13;8(1):129. doi: 10.1186/s13073-016-0384-y 27964755 PMC5154134

[21] Liberzon A, Birger C, Thorvaldsdóttir H, Ghandi M, Mesirov JP, Tamayo P. The Molecular Signatures Database Hallmark Gene Set Collection. Cell Syst 2015; 1: 417–25. 26771021 10.1016/j.cels.2015.12.004 PMC4707969

[22] Bulik-Sullivan BK, Loh PR, Finucane HK, et al. LD Score regression distinguishes confounding from polygenicity in genome-wide association studies. Nat Genet. 2015 Mar;47(3):291–5. doi: 10.1038/ng.3211 25642630 PMC4495769

[23] Shah RL, Guggenheim JA, UK Biobank Eye and Vision Consortium. Genome-wide association studies for corneal and refractive astigmatism in UK Biobank demonstrate a shared role for myopia susceptibility loci. Hum Genet. 2018 Dec; 137(11–12):881–896. doi: 10.1007/s00439-018-1942-8 30306274 PMC6267700

[24] Grytz R, Yang H, Hua Y, Samuels BC, Sigal IA. Connective Tissue Remodeling in Myopia and its Potential Role in Increasing Risk of Glaucoma. Curr Opin Biomed Eng. 2020 Sep;15:40–50. doi: 10.1016/j.cobme.2020.01.001 32211567 PMC7093055

[25] Mégarbané A, Hanna N, Chouery E, Jalkh N, Mehawej C, Boileau C. Marfanoid habitus, inguinal hernia, advanced bone age, and distinctive facial features: a new collagenopathy? Am J Med Genet A. 2012 May;158A(5):1185–9. doi: 10.1002/ajmg.a.35279 22489068

[26] Halal F, Gervais MH, Baillargeon J, Lesage R. Gastro-cutaneous syndrome: peptic ulcer/hiatal hernia, multiple lentigines/café-au-lait spots, hypertelorism, and myopia. Am J Med Genet. 1982 Feb;11(2):161–76. 7065007 10.1002/ajmg.1320110206

[27] Livingstone I, Uversky VN, Furniss D, Wiberg A. The Pathophysiological Significance of Fibulin-3. Biomolecules. 2020; 10(9):1294. doi: 10.3390/biom10091294 32911658 PMC7563619

[28] Kobayashi N, Kostka G, Garbe JH, Keene DR, Bächinger HP, Hanisch FG, et al. A comparative analysis of the fibulin protein family. Biochemical characterization, binding interactions, and tissue localization. J Biol Chem. 2007 Apr 20;282(16):11805–16. doi: 10.1074/jbc.M611029200 17324935

[29] McLaughlin P. J. et al. Lack of fibulin-3 causes early aging and herniation, but not macular degeneration in mice. Hum. Mol. Genet. 16, 3059–3070 (2007). doi: 10.1093/hmg/ddm264 17872905

[30] Wiberg A, Ng M, Schmid AB, Smillie RW, Baskozos G, Holmes MV, et al. A genome-wide association analysis identifies 16 novel susceptibility loci for carpal tunnel syndrome. Nat Commun. 2019 Mar 4;10(1):1030. doi: 10.1038/s41467-019-08993-6 30833571 PMC6399342

[31] Ahmed WU, Wiberg A, Ng M, Wang W, Auton A, 23andMe Research Team, et al. Genome-wide association analysis and replication in 810,625 individuals identifies novel therapeutic targets for varicose veins. bioRxiv 2020.05.14.095653. 10.1038/s41467-022-30765-y PMC9163161 35654884

[32] Olafsdottir T, Thorleifsson G, Sulem P, Stefansson OA, Medek H, Olafsson K, et al. Genome-wide association identifies seven loci for pelvic organ prolapse in Iceland and the UK Biobank. Commun Biol. 2020 Mar 17;3(1):129. doi: 10.1038/s42003-020-0857-9 32184442 PMC7078216

[33] Weedon MN, Lango H, Lindgren CM, Wallace C, Evans DM, Mangino M, et al. Genome-wide association analysis identifies 20 loci that influence adult height. Nat Genet. 2008 May;40(5):575–83. doi: 10.1038/ng.121 18391952 PMC2681221

[34] Gong J, Nishimura KK, Fernandez-Rhodes L, Haessler J, Bien S, Graff M, et al. Trans-ethnic analysis of metabochip data identifies two new loci associated with BMI. Int J Obes (Lond). 2018 Mar;42(3):384–390. doi: 10.1038/ijo.2017.304 29381148 PMC5876082

[35] Richter HE, Whitehead N, Arya L, Ridgeway B, Allen-Brady K, Norton P, et al. Genetic contributions to urgency urinary incontinence in women. J Urol. 2015 Jun;193(6):2020–7. doi: 10.1016/j.juro.2014.12.023 25524241 PMC4439377

[36] Segev Y, Auslender R, Feiner B, Lissak A, Lavie O, Abramov Y. Are women with pelvic organ prolapse at a higher risk of developing hernias? Int Urogynecol J Pelvic Floor Dysfunct. 2009 Dec;20(12):1451–3. doi: 10.1007/s00192-009-0968-9 19652899

[37] Mead TJ, Apte SS. ADAMTS proteins in human disorders. Matrix Biol. 2018 Oct;71–72:225–239. doi: 10.1016/j.matbio.2018.06.002 29885460 PMC6146047

[38] Colige A, Nuytinck L, Hausser I, van Essen AJ, Thiry M, Herens C, et al. Novel types of mutation responsible for the dermatosparactic type of Ehlers-Danlos syndrome (Type VIIC) and common polymorphisms in the ADAMTS2 gene. J Invest Dermatol. 2004 Oct;123(4):656–63. doi: 10.1111/j.0022-202X.2004.23406.x 15373769

[39] Tortorella MD, Burn TC, Pratta MA, Abbaszade I, Hollis JM, Liu R, et al. Purification and cloning of aggrecanase-1: a member of the ADAMTS family of proteins. Science. 1999 Jun 4;284(5420):1664–6. doi: 10.1126/science.284.5420.1664 10356395

[40] Hatano E, Fujita T, Ueda Y, Okuda T, Katsuda S, Okada Y, et al. Expression of ADAMTS-4 (aggrecanase-1) and possible involvement in regression of lumbar disc herniation. Spine (Phila Pa 1976). 2006 Jun 1;31(13):1426–32. doi: 10.1097/01.brs.0000219954.67368.be 16741450

[41] Moustakas A, Heldin CH. The regulation of TGFbeta signal transduction. Development. 2009 Nov;136(22):3699–714. doi: 10.1242/dev.030338 . 19855013

[42] Lindsay ME, Schepers D, Bolar NA, Doyle JJ, Gallo E, Fert-Bober J, et al. Loss-of-function mutations in TGFB2 cause a syndromic presentation of thoracic aortic aneurysm. Nat Genet. 2012 Jul 8;44(8):922–7. doi: 10.1038/ng.2349 22772368 PMC3616632

[43] Ritelli M, Chiarelli N, Dordoni C, Quinzani S, Venturini M, Maroldi R, et al. Further delineation of Loeys-Dietz syndrome type 4 in a family with mild vascular involvement and a TGFB2 splicing mutation. BMC Med Genet. 2014 Aug 28;15:91. doi: 10.1186/s12881-014-0091-8 25163805 PMC4236574

[44] Gao XR, Huang H, Nannini DR, Fan F, Kim H. Genome-wide association analyses identify new loci influencing intraocular pressure. Hum Mol Genet. 2018 Jun 15;27(12):2205–2213. doi: 10.1093/hmg/ddy111 29617998 PMC5985721

[45] Gao X, Nannini DR, Corrao K, Torres M, Chen YI, Fan BJ, et al. Genome-wide association study identifies WNT7B as a novel locus for central corneal thickness in Latinos. Hum Mol Genet. 2016 Nov 15;25(22):5035–5045. doi: 10.1093/hmg/ddw319 28171582 PMC6078592

[46] Soler Artigas M, Loth DW, Wain LV, Gharib SA, Obeidat M, Tang W, et al. Genome-wide association and large-scale follow up identifies 16 new loci influencing lung function. Nat Genet. 2011 Sep 25;43(11):1082–90. doi: 10.1038/ng.941 21946350 PMC3267376

[47] Cho MH, McDonald ML, Zhou X, Mattheisen M, Castaldi PJ, Hersh CP, et al. Risk loci for chronic obstructive pulmonary disease: a genome-wide association study and meta-analysis. Lancet Respir Med. 2014 Mar;2(3):214–25. doi: 10.1016/S2213-2600(14)70002-5 24621683 PMC4176924

[48] Lau H, Fang C, Yuen WK, Patil NG. Risk factors for inguinal hernia in adult males: a case-control study. Surgery. 2007 Feb;141(2):262–6. doi: 10.1016/j.surg.2006.04.014 17263984

[49] Blatnik JA, Krpata DM, Novitsky YW, Rosen MJ. Does a history of wound infection predict postoperative surgical site infection after ventral hernia repair? Am J Surg. 2012 Mar;203(3):370–4; discussion 374. doi: 10.1016/j.amjsurg.2011.12.001 22364903

[50] Oshimori N, Li X, Ohsugi M, Yamamoto T. Cep72 regulates the localization of key centrosomal proteins and proper bipolar spindle formation. EMBO J. 2009 Jul 22;28(14):2066–76. doi: 10.1038/emboj.2009.161 19536135 PMC2718283

[51] Choi JS, Kim SR, Jeon YW, Lee KH, Rha HK. Identification of DNA copy number aberrations by array comparative genomic hybridization in patients with ruptured intracranial aneurysms. J Clin Neurosci. 2009 Feb;16(2):295–301. doi: 10.1016/j.jocn.2007.11.015 19056275

[52] Gharahkhani P, Fitzgerald RC, Vaughan TL, Palles C, Gockel I, Tomlinson I, et al. Genome-wide association studies in oesophageal adenocarcinoma and Barrett’s oesophagus: a large-scale meta-analysis. Lancet Oncol. 2016 Oct;17(10):1363–1373. doi: 10.1016/S1470-2045(16)30240-6 27527254 PMC5052458

[53] Gordon C, Kang JY, Neild PJ, Maxwell JD. The role of the hiatus hernia in gastro-oesophageal reflux disease. Aliment Pharmacol Ther. 2004 Oct 1;20(7):719–32. doi: 10.1111/j.1365-2036.2004.02149.x 15379832

[54] Weston AP, Badr AS, Hassanein RS. Prospective multivariate analysis of clinical, endoscopic, and histological factors predictive of the development of Barrett’s multifocal high-grade dysplasia or adenocarcinoma. Am J Gastroenterol. 1999 Dec;94(12):3413–9. doi: 10.1111/j.1572-0241.1999.01602.x 10606296

[55] Castillo D, Puig S, Iglesias M, Seoane A, de Bolós C, Munitiz V, et al. Activation of the BMP4 pathway and early expression of CDX2 characterize non-specialized columnar metaplasia in a human model of Barrett’s esophagus. J Gastrointest Surg. 2012 Feb;16(2):227–37; discussion 237. doi: 10.1007/s11605-011-1758-5 22076569

[56] Becker J, May A, Gerges C, Anders M, Schmidt C, Veits L, et al. The Barrett-associated variants at GDF7 and TBX5 also increase esophageal adenocarcinoma risk. Cancer Med. 2016 May;5(5):888–91. doi: 10.1002/cam4.641 26783083 PMC4864818

[57] Wang QW, Chen ZL, Piao YJ. Mesenchymal stem cells differentiate into tenocytes by bone morphogenetic protein (BMP) 12 gene transfer. J Biosci Bioeng. 2005 Oct;100(4):418–22. doi: 10.1263/jbb.100.418 16310731

[58] Jones GT, Tromp G, Kuivaniemi H, Gretarsdottir S, Baas AF, Giusti B, et al. Meta-Analysis of Genome-Wide Association Studies for Abdominal Aortic Aneurysm Identifies Four New Disease-Specific Risk Loci. Circ Res. 2017 Jan 20;120(2):341–353. doi: 10.1161/CIRCRESAHA.116.308765 27899403 PMC5253231

[59] Maguire LH, Handelman SK, Du X, Chen Y, Pers TH, Speliotes EK. Genome-wide association analyses identify 39 new susceptibility loci for diverticular disease. Nat Genet. 2018 Oct;50(10):1359–1365. doi: 10.1038/s41588-018-0203-z 30177863 PMC6168378

[60] Brett MS, Ng IS, Lim EC, Yong MH, Li Z, Lai A, et al. De novo 3q22.1 q24 deletion associated with multiple congenital anomalies, growth retardation and intellectual disability. Gene. 2013 Mar 15;517(1):82–8. doi: 10.1016/j.gene.2012.12.082 23313878

[61] Wolstenholme J, Brown J, Masters KG, Wright C, English CJ. Blepharophimosis sequence and diaphragmatic hernia associated with interstitial deletion of chromosome 3 (46,XY,del(3)(q21q23)). J Med Genet. 1994 Aug;31(8):647–8. doi: 10.1136/jmg.31.8.647 7815425 PMC1050030

[62] Mekonen HK, Hikspoors JP, Mommen G, Köhler SE, Lamers WH. Development of the ventral body wall in the human embryo. J Anat. 2015 Nov;227(5):673–85. doi: 10.1111/joa.12380 26467243 PMC4609202

[63] Bycroft C, Freeman C, Petkova D, et al. The UK Biobank resource with deep phenotyping and genomic data. Nature 2018; 562: 203–9. doi: 10.1038/s41586-018-0579-z 30305743 PMC6786975

[64] Sudlow C, Gallacher J, Allen N, et al. UK Biobank: An Open Access Resource for Identifying the Causes of a Wide Range of Complex Diseases of Middle and Old Age. PLoS Med 2015; 12. doi: 10.1371/journal.pmed.1001779 25826379 PMC4380465

[65] O’Connell J, Sharp K, Shrine N, et al. Haplotype estimation for biobank-scale data sets. Nat Genet 2016; 48: 817–20. doi: 10.1038/ng.3583 27270105 PMC4926957

[66] Loh PR, Tucker G, Bulik-Sullivan BK, et al. Efficient Bayesian mixed-model analysis increases association power in large cohorts. Nat Genet 2015; 47: 284–90. doi: 10.1038/ng.3190 25642633 PMC4342297

[67] Wang K, L M, H H. ANNOVAR: functional annotation of genetic variants from high-throughput sequencing data. Nucleic Acids Res 2010; 38: e164. doi: 10.1093/nar/gkq603 20601685 PMC2938201

[68] Boyle AP, Hong EL, Hariharan M, et al. Annotation of functional variation in personal genomes using RegulomeDB. Genome Res 2012; 22: 1790–7. doi: 10.1101/gr.137323.112 22955989 PMC3431494

[69] Rentzsch P, Witten D, Cooper GM, Shendure J, Kircher M. CADD: predicting the deleteriousness of variants throughout the human genome. Nucleic Acids Res 2018; 47: D886–94. 10.1093/nar/gky1016 PMC6323892 30371827

[70] Ernst J, Kellis M. Chromatin-state discovery and genome annotation with ChromHMM. Nat Protoc 2017; 12: 2478–92. doi: 10.1038/nprot.2017.124 29120462 PMC5945550

[71] Cunningham F, Achuthan P, Akanni W, et al. Ensembl 2019. Nucleic Acids Res 2019; 47: D745–51. doi: 10.1093/nar/gky1113 30407521 PMC6323964

[72] Zhu Z, Zhang F, Hu H, et al. Integration of summary data from GWAS and eQTL studies predicts complex trait gene targets. Nat Genet 2016; 48: 481–7. doi: 10.1038/ng.3538 27019110

[73] Welter D, MacArthur J, Morales J, Burdett T, Hall P, Junkins H, et al. The NHGRI GWAS Catalog, a curated resource of SNP-trait associations. Nucleic Acids Res. 2014 Jan;42(Database issue):D1001–6. doi: 10.1093/nar/gkt1229 24316577 PMC3965119

