# Supplementary material for: Shared genetic architecture of hernias: A genome-wide association study with multivariable meta-analysis of multiple hernia phenotypes
Source: PLoS One. 2022 Dec 30;17(12):e0272261. doi: 10.1371/journal.pone.0272261 (PMC9803250; doi:10.1371/journal.pone.0272261)
Supplement: S1 Table — Numbers of total and sex-specific cases and controls are detailed for each of the six cohorts analysed. (PDF) [file pone.0272261.s001.pdf]

S1 SUPPLEMENTARY RESULTS AND TABLES

S1 Table 1. Sex distribution across all four individual hernia cohorts, overlap and umbrella hernia cohorts. Numbers of total and sex-specific cases and controls are detailed for each of the six cohorts analysed.

Hernia Phenotype	Cases, total (*n*)	Controls, total (*n*)	Cases, male (*n*)	Controls, male (*n*)	Cases, female (*n*)	Controls, female (*n*)	Cases, female (%)	Controls, female (%)	Total, female (%)
**Inguinal**	18791	93955	17199	63964	1592	29991	8.5	31.9	28.0
**Femoral**	973	4865	300	1265	673	3600	69.2	74.0	73.2
**Umbilical**	5356	26780	3321	12662	2035	14118	38.0	52.7	50.3
**Hiatus**	32298	161490	12840	52757	19458	108733	60.2	67.3	66.2
**Overlap**	5219	26095	4221	15942	998	10153	19.1	38.9	35.6
**Umbrella**	62637	313185	37881	146590	24756	166595	39.5	53.2	50.9
