# Supplementary material for: Shared genetic architecture of hernias: A genome-wide association study with multivariable meta-analysis of multiple hernia phenotypes
Source: PLoS One. 2022 Dec 30;17(12):e0272261. doi: 10.1371/journal.pone.0272261 (PMC9803250; doi:10.1371/journal.pone.0272261)
Supplement: S2 Table — aBased on NCBI Genome Build 37 (hg19).bThe effect allele. cThe non-effect allele. dThe effect allele frequency. eThe SNP INFO score for imputed SNPs; G = genotyped SNP. fThe genes prioritised at these loci based on positional mapping, eQTL mapping, MAGMA gene mapping and summary-based mendelian randomisation (see Methods). #Denotes the four residual significant signals following conditional regression analysis at the lead SNP at the locus. Bold loci are those that have not been previously reported. (PDF) [file pone.0272261.s002.pdf]

S1 Table 2. Twenty-eight signals at 24 loci associated with inguinal hernia in 18,791 cases and 93,955 controls in UK Biobank

Chromosome	Position[a]	rsID	EA[b]	NEA[c]	EAF[d]	Info[e]	OR (95% CI)	P-value	Mapped genes[f]
1p36.22	9443340	rs1106370	A	G	0.42	0.990	1.07 (1.04-1.09)	1.0×10^{-8}	*SPSB1*
1q41	219734960	rs2820441	C	A	0.32	G	1.09 (1.06-1.11)	6.6×10^{-13}	-
**2p21**	**43665943**	**rs76684055**	**G**	**A**	**0.90**	**0.998**	**1.12 (1.08-1.16)**	$\mathbf{2.8\times10^{-10}}$	***THADA, ZFP36L2***
2p16.1[#]	56102744	rs11899888	G	A	0.16	0.987	1.16 (1.13-1.20)	2.2×10^{-12}	*EFEMP1, PNPT1*
2p16.1	56106928	rs59985551	C	T	0.78	0.998	1.19 (1.16-1.22)	4.7×10^{-40}	*EFEMP1, PNPT1*
2p16.1[#]	56197200	rs7564964	A	G	0.57	0.995	1.13 (1.10-1.15)	2.2×10^{-10}	-
3p14.3	55602137	rs61613824	A	T	0.37	0.987	1.08 (1.05-1.10)	1.1×10^{-10}	*ERC2*
3p14.3	56141843	rs7647972	C	G	0.70	0.991	1.09 (1.06-1.11)	8.9×10^{-12}	*CCDC66, ERC2*
**3q12.2**	**100297679**	**rs13083051**	**T**	**C**	**0.92**	**0.986**	**1.12 (1.08-1.17)**	$\mathbf{2.9\times10^{-8}}$	***TMEM45A***
4p16.2	4949339	rs4330303	G	A	0.68	0.975	1.07 (1.04-1.09)	2.4×10^{-8}	-
4q34.1	174616174	rs56063997	C	T	0.36	0.988	1.07 (1.05-1.10)	3.6×10^{-10}	-
5q12.3	64355060	rs370763	A	T	0.67	0.998	1.10 (1.08-1.13)	3.3×10^{-17}	*ADAMTS6, CWC27*
6p25.1	6743149	rs1294421	T	G	0.40	G	1.07 (1.05-1.10)	5.6×10^{-10}	-
6p22.2	26099279	rs13212652	T	G	0.87	1.000	1.12 (1.08-1.15)	3.1×10^{-11}	*BTN2A1, BTN3A2, C6orf15, HFE, HIST1H1A, HIST1H1B, HIST1H1C, HIST1H1T, HIST1H2AB, HIST1H2AC, HIST1H2AJ, HIST1H2AL, HIST1H2BB, HIST1H2BC, HIST1H2BL, HIST1H2BN, HIST1H3A, HIST1H3B, HIST1H3C, HIST1H3I, HIST1H3J, HIST1H4A, HIST1H4B, HIST1H4C, HIST1H4L, LRRC16A, OR2B2, PGBD1, SCGN, SLC17A1, SLC17A2, SLC17A3, SLC17A4, TRIM26, TRIM31, TRIM38, ZKSCAN3, ZKSCAN4, ZKSCAN8, ZNF165, ZNF322, ZSCAN12, ZSCAN16, ZSCAN31, ZSCAN9*

6p21.32	32808299	rs45506201	G	A	0.90	0.996	1.11 (1.07-1.15)	5.6×10^{-9}	*APOM, BRD2, C4A, C4B, C6orf10, GPANK1, HLA-DMA, HLA-DMB, HLA-DOB, HLA-DQB1, HLA-DQB2, HLA-DRA, HLA-DRB5, LSM2, LY6G5B, MICB, MSH5, MSH5-SAPCD1, NOTCH4, PSMB8, PSMB9, RNF5, SKIV2L, TAP1, TAP2, VARS, VWA7, XXbac-BPG181M17.5*
**6p21.1**	**45481873**	**rs62400367**	**A**	**G**	**0.85**	**0.983**	**1.09 (1.06-1.12)**	**2.9×10^{-8}**	***RUNX2***
6q24.2	143676186	rs6570555	A	T	0.43	0.995	1.08 (1.06-1.11)	7.8×10^{-13}	*AIG1*
**7p15.2**	**25681464**	**rs10951081**	**C**	**A**	**0.33**	**0.968**	**1.07 (1.05-1.10)**	**5.3×10^{-9}**	**-**
7q11.23	73540726	rs3895707	C	T	0.91	0.987	1.11 (1.07-1.15)	2.3×10^{-8}	*ELN, LIMK1*
8p21.2[#]	25435170	rs10481336	C	T	0.21	0.986	1.10 (1.08-1.13)	1.6×10^{-15}	*CDCA2, DOCK5, GNRH1, KCTD9*
8p21.2	25717620	rs6983815	A	T	0.41	0.996	1.19 (1.17-1.22)	1.1×10^{-54}	*EBF2*
9p22.2	16766118	rs7850168	C	A	0.08	0.980	1.12 (1.08-1.17)	1.7×10^{-8}	*BNC2*
11p13[#]	32350027	rs7924571	C	A	0.78	0.995	1.08 (1.06-1.11)	8.8×10^{-9}	*CCDC73, EIF3M*
11p13	32459228	rs4140413	G	T	0.63	0.988	1.11 (1.09-1.14)	2.4×10^{-20}	*CCDC73, EIF3M, WT1*
**12q14.3**	**66328027**	**rs12810758**	**T**	**C**	**0.23**	**0.991**	**1.08 (1.05-1.11)**	**3.2×10^{-9}**	***AC090673.2, HMGA2***
**13q13.1**	**32398964**	**rs796861335**	**C**	**CT**	**0.41**	**0.960**	**1.06 (1.04-1.09)**	**4.6×10^{-8}**	***FRY, RXFP2***
16q24.1	84856552	rs4238714	C	T	0.42	0.992	1.09 (1.06-1.11)	2.8×10^{-13}	*CRISPLD2*
17p12	12191339	rs12453693	T	C	0.31	0.995	1.08 (1.06-1.11)	3.0×10^{-11}	-

[a]*Based on NCBI Genome Build 37 (hg19).*
[b]*The effect allele.*
[c]*The non-effect allele.*
[d]*The effect allele frequency.*
[e]*The SNP INFO score for imputed SNPs; G = genotyped SNP.*
[f]*The genes prioritised at these loci based on positional mapping, eQTL mapping, MAGMA gene mapping and summary-based mendelian randomisation (see Methods).*
[#]*Denotes the four residual significant signals following conditional regression analysis at the lead SNP at the locus.*
Bold loci are those that have not been previously reported.
