# Supplementary material for: Shared genetic architecture of hernias: A genome-wide association study with multivariable meta-analysis of multiple hernia phenotypes
Source: PLoS One. 2022 Dec 30;17(12):e0272261. doi: 10.1371/journal.pone.0272261 (PMC9803250; doi:10.1371/journal.pone.0272261)
Supplement: S3 Table — aBased on NCBI Genome Build 37 (hg19). bThe effect allele. cThe non-effect allele. dThe effect allele frequency. eThe SNP INFO score for imputed SNPs; G = genotyped SNP. f No genes were prioritised at this locus based on positional mapping, eQTL mapping, MAGMA gene mapping and summary-based mendelian randomisation (see Methods). (PDF) [file pone.0272261.s003.pdf]

S1 Table 3. One locus significantly associated with femoral hernia in 973 cases and 4,865 controls in UK Biobank.

Chromosome	Position[a]	rsID	EA[b]	NEA[c]	EAF[d]	Info[e]	OR (95% CI)	P-value	Mapped genes[f]
1q41	219788530	rs7538503	G	A	0.29	0.995	1.42 (1.27-1.58)	1.3×10^{-10}	-

[a]*Based on NCBI Genome Build 37 (hg19).*
[b]*The effect allele.*
[c]*The non-effect allele.*
[d]*The effect allele frequency.*
[e]*The SNP INFO score for imputed SNPs; G = genotyped SNP.*
[f] *No genes were prioritised at this locus based on positional mapping, eQTL mapping, MAGMA gene mapping and summary-based mendelian randomisation (see Methods).*
