# Supplementary material for: Shared genetic architecture of hernias: A genome-wide association study with multivariable meta-analysis of multiple hernia phenotypes
Source: PLoS One. 2022 Dec 30;17(12):e0272261. doi: 10.1371/journal.pone.0272261 (PMC9803250; doi:10.1371/journal.pone.0272261)
Supplement: S4 Table — aBased on NCBI Genome Build 37 (hg19). bThe effect allele. cThe non-effect allele. dThe effect allele frequency. eThe SNP INFO score for imputed SNPs; G = genotyped SNP. fOne gene was prioritised at these loci based on positional mapping and MAGMA gene mapping (see Methods). Bold loci are those that have not been previously reported. (PDF) [file pone.0272261.s004.pdf]

S1 Table 4. Five loci significantly associated with umbilical hernia in 5,356 cases and 26,780 controls in UK Biobank.

Chromosome	Position[a]	rsID	EA[b]	NEA[c]	EAF[d]	Info[e]	OR (95% CI)	P-value	Mapped genes[f]
1q41	219750717	rs4846567	T	G	0.31	G	1.22 (1.17-1.28)	1.7×10^{-18}	-
2q22.3	146365492	No rsID	C	CAA	0.56	0.990	1.13 (1.08-1.17)	2.5×10^{-8}	-
**2q33.1**	**199676405**	**rs778276885**	**AT**	**A**	**0.51**	**0.996**	**1.12 (1.08-1.17)**	$\mathbf{3.9\times10^{-8}}$	**-**
7q33	134591097	rs12707188	T	C	0.37	0.998	1.19 (1.14-1.24)	5.0×10^{-15}	*CALD1*
12q21.2	78154757	rs2887596	T	C	0.47	0.998	1.12 (1.08-1.17)	2.7×10^{-8}	-

[a]*Based on NCBI Genome Build 37 (hg19).*
[b]*The effect allele.*
[c]*The non-effect allele.*
[d]*The effect allele frequency.*
[e]*The SNP INFO score for imputed SNPs; G = genotyped SNP.*
[f]*One gene was prioritised at these loci based on positional mapping and MAGMA gene mapping (see Methods).*
Bold loci are those that have not been previously reported.
