# Supplementary material for: Shared genetic architecture of hernias: A genome-wide association study with multivariable meta-analysis of multiple hernia phenotypes
Source: PLoS One. 2022 Dec 30;17(12):e0272261. doi: 10.1371/journal.pone.0272261 (PMC9803250; doi:10.1371/journal.pone.0272261)
Supplement: S5 Table — aBased on NCBI Genome Build 37 (hg19). bThe effect allele. cThe non-effect allele. dThe effect allele frequency. eThe SNP INFO score for imputed SNPs; G = genotyped SNP. fThe 15 genes prioritised at these loci based on positional mapping, eQTL mapping and MAGMA gene mapping (see Methods). Bold loci are those that have not been previously reported. (PDF) [file pone.0272261.s005.pdf]

S1 Table 5. Eight loci significantly associated with hiatus hernia in 32,298 cases and 161,490 controls in UK Biobank.

Chromosome	Position[a]	rsID	EA[b]	NEA[c]	EAF[d]	Info[e]	OR (95% CI)	P-value	Mapped genes[f]
2p16.1	56040035	rs10207635	T	A	0.13	1.000	1.07 (1.05-1.10)	1.3×10^{-8}	*EFEMP1*
**3p13**	**70920485**	**rs4499560**	**A**	**T**	**0.31**	**0.984**	**1.07 (1.05-1.08)**	$\mathbf{8.9\times10^{-12}}$	**-**
**5p15.32**	**4977446**	**rs42202**	**A**	**G**	**0.08**	**0.986**	**1.14 (1.10-1.18)**	$\mathbf{8.0\times10^{-16}}$	**-**
6p22.2	26582327	rs9393735	A	G	0.86	G	1.07 (1.05-1.10)	2.7×10^{-8}	*BTN2A1, BTN3A2, HIST1H2BN, HIST1H4L, HMGN4, OR2B2, ZNF311, ZNF391*
7q33	134605106	rs4728341	T	C	0.55	0.965	1.06 (1.04-1.07)	3.9×10^{-10}	*CALD1*
**9q22.31**	**96624645**	**rs4075733**	**C**	**T**	**0.46**	**0.996**	**1.05 (1.04-1.07)**	$\mathbf{1.5\times10^{-9}}$	**-**
11p13	32479807	rs11031796	G	A	0.62	0.998	1.07 (1.06-1.09)	3.6×10^{-16}	*WT1*
**19p13.11**	**18787981**	**rs2891698**	**G**	**A**	**0.47**	**0.998**	**1.06 (1.04-1.07)**	$\mathbf{4.0\times10^{-10}}$	***CRTC1, KLHL26, TMEM59L, UBA52***

[a]Based on NCBI Genome Build 37 (hg19).
[b]The effect allele.
[c]The non-effect allele.
[d]The effect allele frequency.
[e]The SNP INFO score for imputed SNPs; G = genotyped SNP.
[f]The 15 genes prioritised at these loci based on positional mapping, eQTL mapping and MAGMA gene mapping (see Methods).
Bold loci are those that have not been previously reported.
