# Supplementary material for: Shared genetic architecture of hernias: A genome-wide association study with multivariable meta-analysis of multiple hernia phenotypes
Source: PLoS One. 2022 Dec 30;17(12):e0272261. doi: 10.1371/journal.pone.0272261 (PMC9803250; doi:10.1371/journal.pone.0272261)
Supplement: S6 Table — 14 genome-wide significant exonic SNPs associated with inguinal, umbilical and hiatus hernia that were identified by FUMA SNP2GENE. All exonic SNPs are in high linkage with the index SNP at each locus (r2 > 0.6). Non-synonymous missense SNPs predicted to be damaging and deleterious by PolyPhen and SIFT are highlighted in blue. (PDF) [file pone.0272261.s006.pdf]

S1 Table 6. Individual hernia associated exonic variants. 14 genome-wide significant exonic SNPs associated with inguinal, umbilical and hiatus hernia that were identified by FUMA SNP2GENE. All exonic SNPs are in high linkage with the index SNP at each locus ($r^2 > 0.6$). Non-synonymous missense SNPs predicted to be damaging and deleterious by PolyPhen and SIFT are highlighted in blue.

Inguinal hernia

rsID	Chr	Position	EA	NEA	EAF	OR	P	Index SNP	r2	*Nearest Gene*	CADD	RDB	Functionality	HGVSp	GERP	Polyphen	SIFT
rs149290349	2	43451957	G	A	0.92	1.13	3.50×10^{-9}	rs76684055	0.67	*THADA:ZFP36L2*	18.15	2b	Missense	p.Ala329Val	1.47	Unknown	Deleterious (Low Confidence)
rs7578597	2	43732823	T	C	0.89	1.10	4.20×10^{-8}	rs76684055	0.88	*THADA*	23.50	1f	Missense	p.Thr1187Ala	4	Possibly Damaging	Deleterious
rs1892251	6	25769349	C	T	0.87	1.10	4.00×10^{-9}	rs13212652	0.67	*SLC17A4*	5.92	7	Synonymous	-	-	-	-
rs34525648	6	25914853	G	A	0.88	1.11	6.40×10^{-10}	rs13212652	0.77	*SLC17A2*	15.35	6	Missense	p.Ser370Leu	-3.2	Possibly Damaging	Tolerated
rs17528178	6	25983777	C	T	0.90	1.12	1.30×10^{-9}	rs13212652	0.64	*TRIM38*	0.63	NA	Synonymous	-	-	-	-
rs16891235	6	26017542	T	C	0.87	1.11	5.00×10^{-11}	rs13212652	0.99	*HIST1H1A*	11.55	1f	Missense	p.Lys140Arg	-5.17	Unknown	Tolerated
rs3752417	6	26045905	G	C	0.88	1.11	3.20×10^{-10}	rs13212652	0.86	*HIST1H3C*	14.99	4	Synonymous	-	-	-	-
rs1812594	8	25287397	C	T	0.21	1.09	6.80×10^{-10}	rs10481336	0.82	*KCTD9*	14.64	NA	Synonymous	-	-	-	-
rs3829009	8	25364834	T	A	0.20	1.13	5.70×10^{-12}	rs10481336	0.94	*CDCA2*	11.70	1f	Missense	p.Arg884Ser	-2.56	Benign	Tolerated
rs6990278	8	25364960	G	A	0.20	1.10	5.00×10^{-12}	rs10481336	0.94	*CDCA2*	6.57	1f	Synonymous	-	-	-	-
rs10866845	8	25708267	C	T	0.41	1.19	2.10×10^{-54}	rs6983815	0.98	*EBF2*	0.00	7	Synonymous	-	-	-	-

Umbilical hernia

rsID	Chr	Position	EA	NEA	EAF	OR	P	Index SNP	r2	Nearest Gene	CADD	RDB	Functionality	HGVSp	GERP	Polyphen	SIFT

rs6973420	7	134618710	G	A	0.48	1.14	2.70×10^{-10}	rs12707188	0.61	*CALD1*	0.01	4	Missense	p.His397Arg	-3.93	benign	Deleterious (Low Confidence)

Hiatus hernia

rsID	Chr	Position	EA	NEA	EAF	OR	P	Index SNP	r2	Nearest Gene	CADD	RDB	Functionality	HGVSp	GERP	Polyphen	SIFT
rs6973420	7	134618710	A	G	0.52	1.05	2.40×10^{-8}	rs4728341	1.00	*CALD1*	0.01	4	Missense	p.His397Arg	-3.93	benign	Deleterious (Low Confidence)

Candidate gene mapping of Individual hernia cohort susceptibility loci

A total of 117 unique genes were prioritised using the four mapping strategies (positional mapping, eQTL mapping, MAGMA gene mapping and summary-based mendelian randomisation) at 12 of 38 susceptibility loci associated with the four individual hernia phenotypes. For inguinal hernia individual cohort, 101 unique genes were mapped to 18 of the 24 inguinal hernia associated loci by positional mapping (n = 53), eQTL mapping in GTEx25 v8 Cells Transformed Fibroblast and Skeletal Muscle Tissue (n = 42), MAGMA26 (n = 64), and Summary-based Mendelian Randomisation (SMR) using GTEx v7 Cells Transformed Fibroblast and Skeletal Muscle Tissue (n = 3) (See Tables S6-S11). Of these, 29 genes were mapped with 2 or more strategies and 4 genes were mapped utilising three strategies (*THADA*, *EFEMP1*, *HLA-DMA* and *CRISPLD2*) (S2 Supplementary Figure 3). For femoral hernia, no genes were prioritised at the 1q41 locus, although the index signal rs7538503 ((p = 1.7×10-18, OR = 1.22, CADD = 14.9), a regulatory region variant, narrowly resides outside the positional mapping window from *ZC3H11B*. For umbilical hernia, *CALD1* was singly prioritised at the 7q33 locus by both positional and MAGMA mapping. For hiatus hernia, 15 unique genes were prioritised at 5 of 8 genome-wide significant susceptibility loci by position (n = 5), eQTL (n = 4), and MAGMA mapping (n = 11), with 5 putative genes mapped by more than one approach (*BTN3A2, CALD1, WT1, KLHL26, CRTC1*) (S2 Supplementary Figure 4).
