# Supplementary material for: Shared genetic architecture of hernias: A genome-wide association study with multivariable meta-analysis of multiple hernia phenotypes
Source: PLoS One. 2022 Dec 30;17(12):e0272261. doi: 10.1371/journal.pone.0272261 (PMC9803250; doi:10.1371/journal.pone.0272261)
Supplement: S7 Table — 138 genome-wide significant intronic and intergenic variants predicted to be deleterious according to a CADD ≥ 12.37, and associated with inguinal, femoral, umbilical and hiatus hernia as identified by FUMA SNP2GENE. Functional variants with a RegulomeDB score of 2b or less are highlighted in blue. (PDF) [file pone.0272261.s007.pdf]

S1 Table 7. Predicted functional intronic and intergenic variants associated with the four individual hernia phenotypes. 138 genome-wide significant intronic and intergenic variants predicted to be deleterious according to a CADD ≥ 12.37, and associated with inguinal, femoral, umbilical and hiatus hernia as identified by FUMA SNP2GENE. Functional variants with a RegulomeDB score of 2b or less are highlighted in blue.

Inguinal Hernia

rsID	Chr	Position	A1	A2	A1Freq	P	BETA	SE	Index SNP	r2	Nearest Gene	Functionality	CADD	RDB
**rs12758001**	**1**	**9437151**	**C**	**G**	**0.39**	**2.90×10^{-8}**	**-0.0086**	**0.0015**	**rs1106370**	**0.99**	**SPSB1**	**intergenic**	**17.34**	**2b**
rs5781117	1	219642187	T	TG	0.34	3.80×10^{-9}	0.0095	0.0016	rs2820441	0.60	RP11-95P13.1	intergenic	20.3	NA
rs2791552	1	219652033	A	C	0.33	1.50×10^{-9}	0.0098	0.0016	rs2820441	0.61	RP11-95P13.1	intergenic	18.63	NA
rs2785986	1	219706327	A	G	0.33	5.60×10^{-11}	-0.0106	0.0016	rs2820441	0.89	RP11-95P13.2	intergenic	14.7	NA
rs4846567	1	219750717	G	T	0.29	9.30×10^{-12}	-0.0114	0.0017	rs2820441	0.84	RP11-95P13.2	intergenic	14.9	5
rs2820443	1	219753509	T	C	0.29	9.50×10^{-12}	-0.0114	0.0017	rs2820441	0.84	RP11-95P13.2	intergenic	12.99	NA
rs6743071	2	43586287	T	G	0.08	8.00×10^{-10}	0.0156	0.0025	rs76684055	0.99	THADA	intronic	16.12	5
rs78681698	2	43598160	A	G	0.08	5.80×10^{-10}	0.0158	0.0025	rs76684055	0.99	THADA	intronic	16.48	3a
rs79629200	2	43602932	T	G	0.08	8.90×10^{-10}	0.0156	0.0025	rs76684055	1.00	THADA	intronic	19.08	5
rs11899863	2	43618819	C	T	0.08	7.60×10^{-10}	0.0157	0.0025	rs76684055	1.00	THADA	intronic	13.26	5
rs17030835	2	43686245	C	G	0.09	5.10×10^{-9}	0.0144	0.0025	rs76684055	0.90	THADA	intronic	13.91	7
**rs6746058**	**2**	**43687542**	**T**	**C**	**0.09**	**5.50×10^{-9}**	**0.0144**	**0.0025**	**rs76684055**	**0.90**	**THADA**	**intronic**	**16.47**	**1f**
No rsID	2	43690836	TCCAA TGGTG	T	0.10	4.50×10^{-8}	0.0134	0.0024	rs76684055	0.80	THADA	intronic	18.04	NA
rs72881068	2	43690860	C	T	0.10	1.70×10^{-8}	0.0134	0.0024	rs76684055	0.80	THADA	intronic	15.56	4
rs13405776	2	43739121	C	T	0.09	4.70×10^{-8}	0.0134	0.0025	rs76684055	0.88	THADA	intronic	12.48	5
rs13419380	2	43739184	T	G	0.09	4.20×10^{-8}	0.0135	0.0025	rs76684055	0.88	THADA	intronic	14.52	5
**rs17030967**	**2**	**43748983**	**G**	**A**	**0.09**	**1.90×10^{-8}**	**0.0139**	**0.0025**	**rs76684055**	**0.89**	**THADA**	**intronic**	**16.94**	**1d**
rs6726917	2	43753913	A	C	0.09	4.20×10^{-8}	0.0135	0.0025	rs76684055	0.88	THADA	intronic	12.88	6
rs113156595	2	43759709	C	G	0.09	2.40×10^{-8}	0.0138	0.0025	rs76684055	0.90	THADA	intronic	12.76	7
rs6752931	2	56004219	G	T	0.22	1.90×10^{-29}	0.0213	0.0019	rs59985551	0.68	PNPT1	intergenic	18.77	5

rs4146922	2	56067182	T	A	0.20	1.70×10^{-32}	0.0227	0.0019	rs59985551	0.84	EFEMP1	intergenic	14.74	5
rs3791679	2	56096892	A	G	0.23	5.90×10^{-40}	0.0243	0.0018	rs59985551	1.00	EFEMP1	intronic	17.76	NA
rs7422809	2	56176031	T	C	0.37	2.30×10^{-24}	0.0162	0.0016	rs7564964	0.74	RN7SKP208	intergenic	15.22	5
rs2173623	3	55596916	T	C	0.42	6.60×10^{-9}	-0.0090	0.0016	rs61613824	0.73	ERC2	intronic	19.88	5
rs61613824	3	55602137	T	A	0.34	1.10×10^{-10}	-0.0103	0.0016	rs61613824	1.00	ERC2	intronic	15.19	7
rs7615948	3	56078781	G	A	0.30	1.80×10^{-9}	0.0097	0.0016	rs7647972	0.74	ERC2	intronic	14.9	5
rs13058777	3	56163560	A	T	0.26	3.60×10^{-11}	0.0110	0.0017	rs7647972	0.81	ERC2	intronic	17.29	5
rs7627149	3	56275541	C	T	0.28	4.70×10^{-10}	0.0103	0.0017	rs7647972	0.75	ERC2	intronic	15.28	6
rs36044157	3	56328592	T	G	0.26	1.70×10^{-8}	0.0095	0.0017	rs7647972	0.66	ERC2	intronic	16.64	5
rs6841699	4	174591579	T	C	0.39	1.10×10^{-8}	-0.0090	0.0016	rs56063997	0.83	RANP6	intergenic	21.2	5
No rsID	5	64388906	CAGAA CTTCA	C	0.30	1.10×10^{-15}	-0.0136	0.0017	rs370763	0.66	Y_RNA	intergenic	13.35	NA
rs9276689	6	32751962	C	T	0.08	3.40×10^{-8}	0.0129	0.0023	rs45506201	0.69	HLA-DQB2	intergenic	15.38	7
rs9403480	6	143605472	G	T	0.41	3.90×10^{-11}	0.0104	0.0016	rs6570555	0.85	AIG1	intronic	19.26	5
rs2216917	6	143677306	A	G	0.43	8.00×10^{-13}	0.0111	0.0016	rs6570555	1.00	RP1-95L4.4	intergenic	12.41	5
rs9644098	8	25394154	A	G	0.29	4.70×10^{-10}	-0.0106	0.0017	rs10481336	0.66	CDCA2	intergenic	18.69	5
rs17053917	8	25405187	T	C	0.29	4.30×10^{-10}	-0.0106	0.0017	rs10481336	0.67	CDCA2	intergenic	19.5	6
rs75194519	8	25420831	T	A	0.21	2.80×10^{-12}	-0.0133	0.0019	rs10481336	0.97	CDCA2	intergenic	12.45	7
rs6986087	8	25675953	A	T	0.41	1.50×10^{-46}	-0.0228	0.0016	rs6983815	0.79	RP11-299D14.2	intergenic	14.47	5
rs75444263	8	25707171	T	TTTTGC TGTCG	0.46	4.80×10^{-53}	-0.0239	0.0016	rs6983815	0.97	EBF2	intronic	14.58	NA
rs4618702	8	25708820	G	T	0.45	1.80×10^{-54}	-0.0242	0.0016	rs6983815	1.00	EBF2	intronic	14.94	7
rs4524799	8	25712812	T	C	0.46	4.00×10^{-54}	-0.0241	0.0016	rs6983815	0.99	EBF2	intronic	19.54	5
rs7850168	9	16766118	C	A	0.07	1.70×10^{-8}	0.0159	0.0028	rs7850168	1.00	BNC2	intronic	16.05	6
rs1460123	12	66322322	C	A	0.25	6.40×10^{-9}	-0.0106	0.0018	rs12810758	1.00	HMGA2	intronic	13.43	5
rs12422566	12	66368872	G	C	0.24	1.90×10^{-8}	-0.0108	0.0019	rs12810758	0.87	HMGA2	intergenic	15.24	7

Femoral Hernia

rsID	Chr	Position	A1	A2	A1Freq	P	BETA	SE	Index SNP	r2	Nearest Gene	Functionality	CADD	RDB
rs2785986	1	219706327	A	G	0.33	1.90×10^{-8}	-0.0404	0.0072	rs7538503	0.66	RP11-95P13.2	intergenic	14.7	NA
rs4846567	1	219750717	G	T	0.29	3.30×10^{-9}	-0.0443	0.0075	rs7538503	0.83	RP11-95P13.2	intergenic	14.9	5
rs2820443	1	219753509	T	C	0.29	2.20×10^{-9}	-0.0449	0.0075	rs7538503	0.85	RP11-95P13.2	intergenic	12.99	NA

Umbilical Hernia

rsID	Chr	Position	A1	A2	A1Freq	P	BETA	SE	Index SNP	r2	Nearest Gene	Functionality	CADD	RDB
rs2785986	1	219706327	A	G	0.33	1.10×10^{-14}	-0.0235	0.0030	rs4846567	0.75	RP11-95P13.2	intergenic	14.7	NA
rs4846567	1	219750717	G	T	0.29	1.70×10^{-18}	-0.0276	0.0031	rs4846567	1.00	RP11-95P13.2	intergenic	14.9	5
rs2820443	1	219753509	T	C	0.29	2.90×10^{-18}	-0.0274	0.0031	rs4846567	0.98	RP11-95P13.2	intergenic	12.99	NA
**rs12532492**	**7**	**134565347**	**C**	**A**	**0.38**	**1.40×10^{-8}**	**-0.0170**	**0.0030**	**rs12707188**	**0.64**	**CALD1**	**intronic**	**16.02**	**1f**
rs11562045	7	134592554	C	G	0.37	8.60×10^{-15}	-0.0235	0.0030	rs12707188	1.00	CALD1	intronic	17.86	4

Hiatus Hernia

rsID	Chr	Position	A1	A2	A1Freq	P	BETA	SE	Index SNP	r2	Nearest Gene	Functionality	CADD	RDB
**rs1522552**	**3**	**70907252**	**A**	**G**	**0.31**	**3.40×10^{-11}**	**0.0086**	**0.0013**	**rs4499560**	**0.96**	**AC096971.1**	**intergenic**	**19.71**	**2b**
rs2597302	3	70917189	T	G	0.29	1.90×10^{-10}	0.0084	0.0013	rs4499560	0.84	AC096971.1	intergenic	14.24	7
rs6776545	3	70923584	A	G	0.29	1.60×10^{-10}	-0.0083	0.0013	rs4499560	0.84	AC096971.1	intergenic	12.41	5
rs9393735	6	26582327	A	G	0.13	2.70×10^{-8}	0.0095	0.0017	rs9393735	1.00	ABT1	intergenic	12.51	7
rs11562045	7	134592554	C	G	0.37	1.40×10^{-8}	0.0070	0.0012	rs4728341	0.61	CALD1	intronic	17.86	4
rs10761288	9	96609363	G	A	0.41	8.10×10^{-9}	0.0070	0.0012	rs4075733	0.77	RP11-53B5.1	intergenic	13.57	7
No rsID	11	32496942	GT	G	0.45	5.00×10^{-14}	0.0091	0.0012	rs11031796	0.67	WT1-AS	intergenic	13.14	NA
