# Supplementary material for: Shared genetic architecture of hernias: A genome-wide association study with multivariable meta-analysis of multiple hernia phenotypes
Source: PLoS One. 2022 Dec 30;17(12):e0272261. doi: 10.1371/journal.pone.0272261 (PMC9803250; doi:10.1371/journal.pone.0272261)
Supplement: S8 Table — 67 protein-coding genes met the threshold for genome-wide significance (P < 2.64x10^-6^, 0.05/18,917) in this analysis. 59 of the 67 genes lay within the FUMA-defined loci borders and are highlighted in red; genes are arranged in descending order according to the P-value of the MAGMA association. (PDF) [file pone.0272261.s008.pdf]

S1 Table 8. Genome-wide gene-based association analysis for inguinal hernia in MAGMA. 67 protein-coding genes met the threshold for genome-wide significance ($P < 2.64 \times 10^{-6}$, 0.05/18,917) in this analysis. 59 of the 67 genes lay within the FUMA-defined loci borders and are highlighted in red; genes are arranged in descending order according to the P-value of the MAGMA association.

Gene	Chromosome	Number of SNPs	Z-statistic	P-value
EBF2	8	692	14.219	3.47×10^{-46}
ERC2	3	3184	9.8817	2.50×10^{-23}
EFEMP1	2	208	8.8012	6.77×10^{-19}
WT1	11	204	7.2111	2.77×10^{-13}
THADA	2	1213	6.856	3.54×10^{-12}
HIST1H2BN	6	30	6.4779	4.65×10^{-11}
HIST1H4A	6	1	6.4445	5.80×10^{-11}
BNC2	9	1612	6.3046	1.44×10^{-10}
HIST1H4L	6	1	6.1707	3.40×10^{-10}
AIG1	6	602	6.1231	4.59×10^{-10}
HIST1H3J	6	7	6.1069	5.08×10^{-10}
TRIM38	6	47	6.0624	6.71×10^{-10}
SLC17A3	6	154	5.9998	9.88×10^{-10}
HMGA2	12	262	5.982	1.10×10^{-9}
ELN	7	70	5.9703	1.18×10^{-9}
ZKSCAN8	6	53	5.8834	2.01×10^{-9}
HFE	6	30	5.8525	2.42×10^{-9}
CCDC73	11	681	5.8039	3.24×10^{-9}
OR2B2	6	4	5.7282	5.08×10^{-9}
SLC17A1	6	155	5.6167	9.73×10^{-9}

HIST1H1B	6	4	5.6121	9.99×10^{-9}
ZKSCAN4	6	37	5.6106	1.01×10^{-8}
ZNF165	6	23	5.5177	1.72×10^{-8}
ZSCAN16	6	10	5.4995	1.90×10^{-8}
BTN2A1	6	69	5.4966	1.94×10^{-8}
CRISPLD2	16	558	5.4422	2.63×10^{-8}
HIST1H3I	6	1	5.4376	2.70×10^{-8}
ADAMTS6	5	899	5.4345	2.75×10^{-8}
ZSCAN9	6	29	5.4341	2.75×10^{-8}
LTBP4	19	118	5.4131	3.10×10^{-8}
ZSCAN31	6	111	5.4041	3.26×10^{-8}
VWA7	6	29	5.3984	3.36×10^{-8}
MSH5	6	68	5.3966	3.40×10^{-8}
EIF3M	11	75	5.3841	3.64×10^{-8}
RXFP2	13	210	5.3792	3.74×10^{-8}
HIST1H3C	6	3	5.3713	3.91×10^{-8}
HIST1H2AJ	6	4	5.369	3.96×10^{-8}
TRIM31	6	73	5.3371	4.72×10^{-8}
BTN3A2	6	102	5.3293	4.93×10^{-8}
MSH5-SAPCD1	6	67	5.3278	4.97×10^{-8}
MECOM	3	1887	5.2985	5.84×10^{-8}
HIST1H2BL	6	2	5.17	1.17×10^{-7}
ZKSCAN3	6	73	5.1365	1.40×10^{-7}
C6orf15	6	18	5.0851	1.84×10^{-7}
IFT140	16	363	4.9935	2.96×10^{-7}
CDCA2	8	132	4.986	3.08×10^{-7}
HIST1H2AL	6	2	4.9737	3.28×10^{-7}

ZFP36L2	2	12	4.94	$3.91{\times}10^{-7}$
PNPT1	2	250	4.9352	$4.00{\times}10^{-7}$
RP11-463D19.2	8	407	4.9025	$4.73{\times}10^{-7}$
ZSCAN12	6	72	4.8929	$4.97{\times}10^{-7}$
ADCY3	2	414	4.8897	$5.05{\times}10^{-7}$
LRRC16A	6	1603	4.8859	$5.15{\times}10^{-7}$
LSM2	6	32	4.8027	$7.83{\times}10^{-7}$
TRIM26	6	132	4.7593	$9.72{\times}10^{-7}$
CCDC66	3	325	4.7346	$1.10{\times}10^{-6}$
SHKBP1	19	50	4.724	$1.16{\times}10^{-6}$
CWC27	5	533	4.7222	$1.17{\times}10^{-6}$
PGBD1	6	63	4.7022	$1.29{\times}10^{-6}$
SLC17A2	6	63	4.6967	$1.32{\times}10^{-6}$
TMEM204	16	101	4.6902	$1.36{\times}10^{-6}$
HIST1H1A	6	4	4.6872	$1.39{\times}10^{-6}$
HIST1H2BC	6	32	4.6699	$1.51{\times}10^{-6}$
VARS	6	39	4.5914	$2.20{\times}10^{-6}$
ZNF322	6	74	4.578	$2.35{\times}10^{-6}$
ARHGEF15	17	45	4.5612	$2.54{\times}10^{-6}$
SLC17A4	6	113	4.5549	$2.62{\times}10^{-6}$
