# Supplementary material for: Shared genetic architecture of hernias: A genome-wide association study with multivariable meta-analysis of multiple hernia phenotypes
Source: PLoS One. 2022 Dec 30;17(12):e0272261. doi: 10.1371/journal.pone.0272261 (PMC9803250; doi:10.1371/journal.pone.0272261)
Supplement: S9 Table — The three probes (genes) that met the Bonferroni-corrected significance threshold PSMR < 1.12×10^−4^ (0.05/4,455) and passed the HEIDI test (PHEIDI ≥ 8.33x10^-3^) (0.05/6)) are shown. eQTL tissues tested were for both GTEx v7 Skeletal muscle and Cells Transformed Fibroblast, however only three probes from skeletal muscle tissue met the HEIDI enrichment threshold. All three were mapped to within the realms of the FUMA-defined susceptibility loci. aProbe ID. bProbe chromosome. cGene name. dSNP name. eAllele 1. fAllele 2. gFrequency of Allele 1 in the study population. hEffect size of the allele iStandard error of the effect size jGWAS P-value. keQTL P-value. lSMR P-value. mHEIDI P-value. Probes mapped to within the realms of the FUMA-defined susceptibility loci are highlighted in red. (PDF) [file pone.0272261.s009.pdf]

S1 Table 9. Summary-based Mendelian Randomisation (SMR) for inguinal hernia using eQTL data from GTEx v7. The three probes (genes) that met the Bonferroni-corrected significance threshold $P_{SMR} < 1.12\times10^{-4}$ (0.05/4,455) and passed the HEIDI test ($P_{HEIDI} \geq 8.33\text{x}10^{-3}$) (0.05/6)) are shown. eQTL tissues tested were for both GTEx v7 Skeletal muscle and Cells Transformed Fibroblast, however only three probes from skeletal muscle tissue met the HEIDI enrichment threshold. All three were mapped to within the realms of the FUMA-defined susceptibility loci.

Probe ID[a]	Chr[b]	Gene[c]	Top SNP[d]	A1[e]	A2[f]	A1Freq[g]	BETA[h]	SE[i]	P_{GWAS}[j]	P_{eQTL}[k]	P_{SMR}[l]	P_{HEIDI}[m]
ENSG00000197279.3	6	ZNF165	rs156739	A	C	0.296754	-0.0089422	0.00167788	9.80×10^{-8}	7.35×10^{-16}	8.74×10^{-6}	3.69×10^{-2}
ENSG00000244731.3	6	C4A	rs389884	G	A	0.132133	-0.0119456	0.00226295	1.30×10^{-7}	2.53×10^{-27}	2.09×10^{-6}	3.50×10^{-2}
ENSG00000204257.10	6	HLA-DMA	rs1480380	T	C	0.104061	-0.0137961	0.00251022	3.90×10^{-8}	1.17×10^{-32}	6.05×10^{-7}	8.23×10^{-2}

[a]*Probe ID.*
[b]*Probe chromosome.*
[c]*Gene name.*
[d]*SNP name.*
[e]*Allele 1.*
[f]*Allele 2.*
[g]*Frequency of Allele 1 in the study population.*
[h]*Effect size of the allele*
[i]*Standard error of the effect size* [j]*GWAS P-value.*
[k]*eQTL P-value.*
[l]*SMR P-value.*
[m]*HEIDI P-value. Probes mapped to within the realms of the FUMA-defined susceptibility loci are highlighted in red.*
