# Supplementary material for: Shared genetic architecture of hernias: A genome-wide association study with multivariable meta-analysis of multiple hernia phenotypes
Source: PLoS One. 2022 Dec 30;17(12):e0272261. doi: 10.1371/journal.pone.0272261 (PMC9803250; doi:10.1371/journal.pone.0272261)
Supplement: S10 Table — 101 unique genes (169 total) were mapped to 21 of 24 inguinal hernia susceptibility loci by one or more gene mapping strategies. 53 genes were mapped via positional mapping, 42 genes were mapped via eQTL mapping, 64 genes were mapped using MAGMA and 3 genes were mapped using summary-based mendelian randomisation. Overlap between the four different mapping strategies is shown (and highlighted in pink). (PDF) [file pone.0272261.s010.pdf]

S1 Table 10. Genes mapped to the inguinal hernia-associated loci using the four mapping strategies. 101 unique genes (169 total) were mapped to 21 of 24 inguinal hernia susceptibility loci by one or more gene mapping strategies. 53 genes were mapped via positional mapping, 42 genes were mapped via eQTL mapping, 64 genes were mapped using MAGMA and 3 genes were mapped using summary-based mendelian randomisation. Overlap between the four different mapping strategies is shown (and highlighted in pink).

Chromosome	Lead SNP	Position	53 FUMA Positionally Mapped Genes	42 FUMA eQTL Mapped Genes	64 MAGMA Mapped Genes	3 SMR Mapped Genes	Number of Gene Mapping Approaches
1	rs1106370	9443340	*SPSB1*				1
2	rs76684055	43665943	*THADA*	*THADA*	*THADA*		3
2	rs76684055	43665943	*ZFP36L2*		*ZFP36L2*		2
2	rs11899888	56102744	*EFEMP1*	*EFEMP1*	*EFEMP1*		3
2	rs11899888	56102744			*PNPT1*		1
2	rs59985551	56106928	*EFEMP1*	*EFEMP1*	*EFEMP1*		3
2	rs59985551	56106928			*PNPT1*		1
3	rs61613824	55602137	*ERC2*		*ERC2*		2
3	rs7647972	56141843	*ERC2*		*ERC2*		2
3	rs7647972	56141843			*CCDC66*		1
3	rs13083051	100297679	*TMEM45A*				1
5	rs370763	64355060			*CWC27*		1
5	rs370763	64355060			*ADAMTS6*		1
6	rs13212652	26099279	*SCGN*				1
6	rs13212652	26099279	*SLC17A4*		*SLC17A4*		2
6	rs13212652	26099279	*SLC17A1*		*SLC17A1*		2
6	rs13212652	26099279	*SLC17A3*		*SLC17A3*		2

6	rs13212652	26099279	*SLC17A2*		*SLC17A2*		2
6	rs13212652	26099279	*TRIM38*		*TRIM38*		2
6	rs13212652	26099279	*HIST1H1A*		*HIST1H1A*		2
6	rs13212652	26099279	*HIST1H3A*				1
6	rs13212652	26099279	*HIST1H4A*		*HIST1H4A*		2
6	rs13212652	26099279	*HIST1H4B*				1
6	rs13212652	26099279	*HIST1H3B*				1
6	rs13212652	26099279	*HIST1H2AB*				1
6	rs13212652	26099279	*HIST1H2BB*				1
6	rs13212652	26099279	*HIST1H3C*		*HIST1H3C*		2
6	rs13212652	26099279	*HIST1H1C*	*HIST1H1C*			2
6	rs13212652	26099279	*HFE*		*HFE*		2
6	rs13212652	26099279	*HIST1H4C*				1
6	rs13212652	26099279	*HIST1H1T*				1
6	rs13212652	26099279	*HIST1H2BC*		*HIST1H2BC*		2
6	rs13212652	26099279	*HIST1H2AC*				1
6	rs13212652	26099279			*LRRC16A*		1
6	rs13212652	26099279		*BTN3A2*	*BTN3A2*		2
6	rs13212652	26099279			*BTN2A1*		1
6	rs13212652	26099279			*ZNF322*		1
6	rs13212652	26099279			*HIST1H2BL*		1
6	rs13212652	26099279			*HIST1H2AJ*		1
6	rs13212652	26099279			*HIST1H2BN*		1
6	rs13212652	26099279			*HIST1H2AL*		1
6	rs13212652	26099279			*HIST1H1B*		1
6	rs13212652	26099279			*HIST1H3I*		1

6	rs13212652	26099279			*HIST1H4L*		1
6	rs13212652	26099279			*HIST1H3J*		1
6	rs13212652	26099279			*OR2B2*		1
6	rs13212652	26099279			*ZNF165*	*ZNF165*	2
6	rs13212652	26099279			*ZSCAN16*		1
6	rs13212652	26099279			*ZKSCAN8*		1
6	rs13212652	26099279			*ZSCAN9*		1
6	rs13212652	26099279			*ZKSCAN4*		1
6	rs13212652	26099279			*PGBD1*		1
6	rs13212652	26099279			*ZSCAN31*		1
6	rs13212652	26099279			*ZKSCAN3*		1
6	rs13212652	26099279			*ZSCAN12*		1
6	rs13212652	26099279			*TRIM31*		1
6	rs13212652	26099279			*TRIM26*		1
6	rs13212652	26099279			*C6orf15*		1
6	rs45506201	32808299	*C6orf10*				1
6	rs45506201	32808299	*HLA-DRA*				1
6	rs45506201	32808299	*HLA-DQB1*	*HLA-DQB1*			2
6	rs45506201	32808299	*HLA-DOB*				1
6	rs45506201	32808299	*TAP2*				1
6	rs45506201	32808299	*PSMB8*				1
6	rs45506201	32808299	*PSMB9*				1
6	rs45506201	32808299	*TAP1*				1
6	rs45506201	32808299	*HLA-DMB*	*HLA-DMB*			2

6	rs45506201	32808299	*XXbac-BPG181M17.5*				1
6	rs45506201	32808299	*HLA-DMA*	*HLA-DMA*		*HLA-DMA*	3
6	rs45506201	32808299	*BRD2*				1
6	rs45506201	32808299			*MSH5*		1
6	rs45506201	32808299			*MSH5-SAPCD1*		1
6	rs45506201	32808299			*VWA7*		1
6	rs45506201	32808299			*VARS*		1
6	rs45506201	32808299			*LSM2*		1
6	rs45506201	32808299		*MICB*			1
6	rs45506201	32808299		*APOM*			1
6	rs45506201	32808299		*GPANK1*			1
6	rs45506201	32808299		*LY6G5B*			1
6	rs45506201	32808299		*SKIV2L*			1
6	rs45506201	32808299		*C4A*		*C4A*	2
6	rs45506201	32808299		*C4B*			1
6	rs45506201	32808299		*RNF5*			1
6	rs45506201	32808299		*NOTCH4*			1
6	rs45506201	32808299		*HLA-DRB5*			1
6	rs45506201	32808299		*HLA-DQB2*			1
6	rs62400367	45481873	*RUNX2*				1
6	rs6570555	143676186	*AIG1*		*AIG1*		2
7	rs3895707	73540726			*ELN*		1
7	rs3895707	73540726	*LIMK1*				1
8	rs10481336	25435170	*DOCK5*				1

8	rs10481336	25435170	*GNRH1*				1
8	rs10481336	25435170	*KCTD9*	*KCTD9*			2
8	rs10481336	25435170	*CDCA2*		*CDCA2*		2
8	rs6983815	25717620	*EBF2*		*EBF2*		2
9	rs7850168	16766118	*BNC2*		*BNC2*		2
11	rs7924571	32350027			*EIF3M*		1
11	rs7924571	32350027			*CCDC73*		1
11	rs4140413	32459228	*WT1*		*WT1*		2
11	rs4140413	32459228			*EIF3M*		1
11	rs4140413	32459228			*CCDC73*		1
12	rs12810758	66328027	*HMGA2*		*HMGA2*		2
12	rs12810758	66328027	*AC090673.2*				1
13	rs796861335	32398964			*RXFP2*		1
13	rs796861335	32398964		*FRY*			1
16	rs4238714	84856552	*CRISPLD2*	*CRISPLD2*	*CRISPLD2*		3
