# Supplementary material for: Shared genetic architecture of hernias: A genome-wide association study with multivariable meta-analysis of multiple hernia phenotypes
Source: PLoS One. 2022 Dec 30;17(12):e0272261. doi: 10.1371/journal.pone.0272261 (PMC9803250; doi:10.1371/journal.pone.0272261)
Supplement: S11 Table — Three protein-coding genes met the threshold for genome-wide significance (p<2.64x10^-6^, 0.05/18,916) in this analysis. The one gene that lays within the realms of the genome-wide significant susceptibility loci and are highlighted in red. (PDF) [file pone.0272261.s011.pdf]

S1 Table 11. Genome-wide gene-based association analysis for umbilical hernia in MAGMA. Three protein-coding genes met the threshold for genome-wide significance ($p<2.64x10^{-6}$, 0.05/18,916) in this analysis. The one gene that lays within the realms of the genome-wide significant susceptibility loci and are highlighted in red.

Gene	Chromosome	Number of SNPs	Z-statistic	P-value
CALD1	7	683	7.4936	3.35×10^{-14}
ADAMTS5	21	156	4.6936	1.34×10^{-6}
CDK5RAP3	17	42	4.6011	2.10×10^{-6}
