# Supplementary material for: Shared genetic architecture of hernias: A genome-wide association study with multivariable meta-analysis of multiple hernia phenotypes
Source: PLoS One. 2022 Dec 30;17(12):e0272261. doi: 10.1371/journal.pone.0272261 (PMC9803250; doi:10.1371/journal.pone.0272261)
Supplement: S12 Table — 26 protein-coding genes met the threshold for genome-wide significance (p<2.64x10^-6^, 0.05/18,918) in this analysis. 11 of the 26 genes lay within the realms of the FUMA-defined genome-wide significant susceptibility loci and are highlighted in red. (PDF) [file pone.0272261.s012.pdf]

S1 Table 12. Genome-wide gene-based association analysis for hiatus hernia in MAGMA. 26 protein-coding genes met the threshold for genome-wide significance ($p<2.64x10^{-6}$, 0.05/18,918) in this analysis. 11 of the 26 genes lay within the realms of the FUMA-defined genome-wide significant susceptibility loci and are highlighted in red.

Gene	Chromosome	Number of SNPs	Z-statistic	P-value
CRTC1	19	283	6.3335	1.20×10^{-10}
KLHL26	19	108	6.2493	2.06×10^{-10}
WT1	11	204	6.2036	2.76×10^{-10}
CALD1	7	683	5.8701	2.18×10^{-9}
GDF5	20	39	5.6753	6.92×10^{-9}
TMEM59L	19	47	5.3728	3.88×10^{-8}
SOX7	8	629	5.3015	5.74×10^{-8}
HLA-B	6	154	5.1937	1.03×10^{-7}
UQCC1	20	194	5.1747	1.14×10^{-7}
SOX7	8	623	5.1714	1.16×10^{-7}
CEP250	20	108	5.162	1.22×10^{-7}
TAF2	8	318	5.0474	2.24×10^{-7}
PINX1	8	452	5.0204	2.58×10^{-7}
HIST1H2BN	6	30	4.9558	3.60×10^{-7}
HIST1H4L	6	1	4.9354	4.00×10^{-7}
PITPNM1	11	10	4.9146	4.45×10^{-7}
OR2B2	6	4	4.8496	6.19×10^{-7}
DSCC1	8	104	4.7968	8.06×10^{-7}
PTH1R	3	56	4.7957	8.11×10^{-7}
PRDM6	5	443	4.7694	9.24×10^{-7}
BTN2A1	6	69	4.7546	9.94×10^{-7}
MYPN	10	491	4.7271	1.14×10^{-6}
FBP2	9	207	4.6699	1.51×10^{-6}

ZNF311	6	33	4.6325	1.81×10^{-6}
DUSP6	12	30	4.6318	1.81×10^{-6}
BTN3A2	6	102	4.6289	1.84×10^{-6}
