# Supplementary material for: Shared genetic architecture of hernias: A genome-wide association study with multivariable meta-analysis of multiple hernia phenotypes
Source: PLoS One. 2022 Dec 30;17(12):e0272261. doi: 10.1371/journal.pone.0272261 (PMC9803250; doi:10.1371/journal.pone.0272261)
Supplement: S13 Table — 15 unique genes (20 total) were mapped to 5 of 8 hiatus hernia susceptibility loci by one or more gene mapping strategies. 5 genes were mapped via positional mapping, 4 genes were mapped via eQTL mapping, 11 genes were mapped using MAGMA and no genes were mapped using summary-based mendelian randomisation. Overlap between the four different mapping strategies is shown (and highlighted in pink). (PDF) [file pone.0272261.s013.pdf]

S1 Table 13. Genes mapped to the hiatus hernia-associated loci using the four mapping strategies. 15 unique genes (20 total) were mapped to 5 of 8 hiatus hernia susceptibility loci by one or more gene mapping strategies. 5 genes were mapped via positional mapping, 4 genes were mapped via eQTL mapping, 11 genes were mapped using MAGMA and no genes were mapped using summary-based mendelian randomisation. Overlap between the four different mapping strategies is shown (and highlighted in pink).

Chromosome	Lead SNP	Position	5 FUMA Positionally Mapped Genes	4 FUMA eQTL Mapped Genes	11 MAGMA Mapped Genes	0 SMR Mapped Genes	Number of Gene Mapping Approaches
2	rs10207635	56040035	*EFEMP1*				1
6	rs9393735	26582327		*BTN3A2*	*BTN3A2*		2
6	rs9393735	26582327			*BTN2A1*		1
6	rs9393735	26582327		*HMGN4*			1
6	rs9393735	26582327		*ZNF391*			1
6	rs9393735	26582327			*HIST1H2BN*		1
6	rs9393735	26582327			*HIST1H4L*		1
6	rs9393735	26582327			*OR2B2*		1
6	rs9393735	26582327			*ZNF311*		1
7	rs4728341	134605106	*CALD1*		*CALD1*		2
11	rs11031796	32479807	*WT1*		*WT1*		2
19	rs2891698	18787981			*TMEM59L*		1
19	rs2891698	18787981	*KLHL26*		*KLHL26*		2
19	rs2891698	18787981	*CRTC1*		*CRTC1*		2
19	rs2891698	18787981		*UBA52*			1
