# Supplementary material for: Shared genetic architecture of hernias: A genome-wide association study with multivariable meta-analysis of multiple hernia phenotypes
Source: PLoS One. 2022 Dec 30;17(12):e0272261. doi: 10.1371/journal.pone.0272261 (PMC9803250; doi:10.1371/journal.pone.0272261)
Supplement: S14 Table — Six genome-wide significant intronic and intergenic variants predicted to be deleterious according to a CADD ≥ 12.37 and associated with overlap hernia as identified by FUMA SNP2GENE. (PDF) [file pone.0272261.s014.pdf]

S1 Table 14. Predicted functional intronic and intergenic variants associated with overlap hernia. Six genome-wide significant intronic and intergenic variants predicted to be deleterious according to a CADD ≥ 12.37 and associated with overlap hernia as identified by FUMA SNP2GENE.

rsID	Chr	Position	A1	A2	A1Freq	P-value	BETA	SE	Index SNP	r2	Nearest Gene	Functionality	CADD	RDB
rs2785986	1	219706327	A	G	0.33	$1.60×10^{-15}$	-0.0244	0.0031	rs1415287	0.76	*RP11-95P13.2*	intergenic	14.70	NA
rs4846567	1	219750717	G	T	0.29	$1.40×10^{-16}$	-0.0262	0.0032	rs1415287	0.99	*RP11-95P13.2*	intergenic	14.9	5
rs2820443	1	219753509	T	C	0.29	$3.40×10^{-16}$	-0.0259	0.0032	rs1415287	0.99	*RP11-95P13.2*	intergenic	12.99	NA
rs3791679	2	56096892	A	G	0.23	$1.40×10^{-17}$	0.0300	0.0035	rs1346786	0.66	*EFEMP1*	intronic	17.76	NA
rs7422809	2	56176031	T	C	0.37	$1.30×10^{-14}$	0.0234	0.0030	rs981037	0.68	*RN7SKP208*	intergenic	15.22	5
No rsID	11	32496942	GT	G	0.45	$4.10×10^{-8}$	0.0163	0.0030	rs3858458	0.67	*WT1-AS*	intergenic	13.14	NA
