# Supplementary material for: Shared genetic architecture of hernias: A genome-wide association study with multivariable meta-analysis of multiple hernia phenotypes
Source: PLoS One. 2022 Dec 30;17(12):e0272261. doi: 10.1371/journal.pone.0272261 (PMC9803250; doi:10.1371/journal.pone.0272261)
Supplement: S15 Table — 38 genome-wide significant intronic and intergenic variants predicted to be deleterious according to a CADD ≥ 12.37, and associated with umbrella hernia as identified by FUMA SNP2GENE. Functional variants with a RegulomeDB score of 2b or less are highlighted in blue. (PDF) [file pone.0272261.s015.pdf]

S1 Table 15. Predicted functional intronic and intergenic variants associated with umbrella hernia. 38 genome-wide significant intronic and intergenic variants predicted to be deleterious according to a CADD ≥ 12.37, and associated with umbrella hernia as identified by FUMA SNP2GENE. Functional variants with a RegulomeDB score of 2b or less are highlighted in blue.

rsID	Chr	Position	A1	A2	A1 Freq	P-value	BETA	SE	Index SNP	r2	Nearest Gene	Functionality	CADD	RDB
rs2799098	1	218521609	G	A	0.20	9.30×10^{-15}	-0.009	0.001	rs2799098	1.00	*TGFB2*	intronic	19.34	NA
rs5781117	1	219642187	T	TG	0.34	1.40×10^{-15}	0.007	0.001	rs2820441	0.60	*RP11-95P13.1*	intergenic	20.3	NA
rs2791552	1	219652033	A	C	0.33	5.10×10^{-15}	0.007	0.001	rs2820441	0.61	*RP11-95P13.1*	intergenic	18.63	NA
rs2785986	1	219706327	A	G	0.33	1.90×10^{-21}	-0.009	0.001	rs2820441	0.89	*RP11-95P13.2*	intergenic	14.7	NA
rs4846567	1	219750717	G	T	0.29	1.10×10^{-22}	-0.009	0.001	rs2820441	0.84	*RP11-95P13.2*	intergenic	14.9	5
rs2820443	1	219753509	T	C	0.29	1.10×10^{-22}	-0.009	0.001	rs2820441	0.84	*RP11-95P13.2*	intergenic	12.99	NA
rs6752931	2	56004219	G	T	0.22	7.60×10^{-21}	0.010	0.001	rs59985551	0.68	*PNPT1*	intergenic	18.77	5
rs4146922	2	56067182	T	A	0.20	1.60×10^{-23}	0.011	0.001	rs59985551	0.84	*EFEMP1*	intergenic	14.74	5
rs3791679	2	56096892	A	G	0.23	5.60×10^{-33}	0.012	0.001	rs59985551	1.00	*EFEMP1*	intronic	17.76	NA
rs7422809	2	56176031	T	C	0.37	1.00×10^{-21}	0.008	0.001	rs13431149	0.77	*RN7SKP208*	intergenic	15.22	5
rs17785206	5	5373748	T	C	0.09	1.80×10^{-11}	-0.009	0.001	rs7715383	0.71	*ALG3P1*	intergenic	15.76	5
No rsID	5	64388906	CAGAA CTTCA	C	0.30	2.90×10^{-10}	-0.006	0.001	rs370763	0.66	*Y_RNA*	intergenic	13.35	NA
rs34104395	6	26478252	C	T	0.08	9.50×10^{-15}	0.010	0.001	rs28360634	0.76	*BTN2A1*	intergenic	13.34	4
**rs13198716**	**6**	**26582035**	**C**	**T**	**0.07**	8.10×10^{-16}	**0.011**	**0.001**	**rs28360634**	**0.89**	***ABT1***	**intergenic**	**12.54**	**1f**
rs13211434	6	26743531	G	C	0.10	3.50×10^{-15}	0.010	0.001	rs28360634	0.81	*RP11-457M11.5*	intergenic	13.07	6
**rs67457459**	**6**	**27198343**	**T**	**G**	**0.10**	4.20×10^{-13}	**0.009**	**0.001**	**rs28360634**	**0.71**	***PRSS16***	**intergenic**	**13.45**	**2b**
rs34573979	6	27480526	C	T	0.07	1.20×10^{-15}	0.011	0.001	rs28360634	0.99	*XXbac-BPGBPG34I8.1*	intergenic	15.35	5
rs56405707	6	27640246	G	A	0.07	1.10×10^{-15}	0.011	0.001	rs28360634	0.99	*RP1-15D7.1*	intergenic	13.34	4
rs13193480	6	27702561	A	G	0.07	3.40×10^{-15}	0.010	0.001	rs28360634	0.97	*RP1-97D16.1*	intergenic	15.29	5

rs71559054	6	27896799	A	C	0.07	8.60×10^{-16}	0.011	0.001	rs28360634	0.95	*OR2W6P*	intergenic	14.09	7
No rsID	6	27914359	AAATGAAC TGAAGGAG AGGTCCC	A	0.07	9.30×10^{-16}	0.011	0.001	rs28360634	0.95	*OR2W6P*	intergenic	13.54	NA
rs13211507	6	28257377	T	C	0.07	3.50×10^{-15}	0.010	0.001	rs28360634	0.86	*PGBD1*	intronic	13.79	6
No rsID	6	28277346	AT	A	0.07	2.00×10^{-15}	0.010	0.001	rs28360634	0.86	*PGBD1*	intergenic	12.91	NA
rs56075693	6	28290328	T	G	0.07	9.60×10^{-16}	0.011	0.001	rs28360634	0.88	*ZSCAN31*	intergenic	12.71	5
rs35744819	6	28318331	G	T	0.07	2.40×10^{-15}	0.011	0.001	rs28360634	0.85	*ZSCAN31:ZKSCAN3*	intronic	13.57	3a
rs1405212	6	117490664	T	C	0.36	7.30×10^{-9}	0.005	0.001	rs200889152	1.00	*VGLL2*	intergenic	17.69	NA
rs9403480	6	143605472	G	T	0.41	7.10×10^{-11}	0.006	0.001	rs6917403	0.88	*AIG1*	intronic	19.26	5
rs2216917	6	143677306	A	G	0.43	1.00×10^{-11}	0.006	0.001	rs6917403	0.97	*RP1-95L4.4*	intergenic	12.41	5
rs3757582	7	73440241	T	C	0.05	1.50×10^{-10}	-0.011	0.002	rs2356532	1.00	*ELN*	intergenic	18.7	5
rs5884929	7	73445306	G	GT	0.05	9.60×10^{-11}	-0.012	0.002	rs2356532	1.00	*ELN*	intronic	16.23	NA
rs6986087	8	25675953	A	T	0.41	5.10×10^{-18}	-0.008	0.001	rs4368985	0.82	*RP11-299D14.2*	intergenic	14.47	5
rs75444263	8	25707171	T	TTTTGC TGTCG	0.46	4.80×10^{-18}	-0.008	0.001	rs4368985	0.94	*EBF2*	intronic	14.58	NA
rs4618702	8	25708820	G	T	0.45	1.80×10^{-18}	-0.008	0.001	rs4368985	0.95	*EBF2*	intronic	14.94	7
rs4524799	8	25712812	T	C	0.46	2.70×10^{-18}	-0.008	0.001	rs4368985	0.95	*EBF2*	intronic	19.54	5
**rs7870070**	**9**	**133039461**	**A**	**G**	**0.50**	**2.10×10^{-8}**	**0.005**	**0.001**	**rs9299329**	**1.00**	***HMCN2***	**intergenic**	**14.07**	**1b**
rs769701	12	89743589	G	A	0.20	7.80×10^{-9}	-0.006	0.001	rs797267	0.99	*DUSP6*	intronic	20.4	4
rs769700	12	89743614	A	G	0.20	7.60×10^{-9}	-0.006	0.001	rs797267	0.99	*DUSP6*	intronic	22.3	NA
rs704076	12	89743921	C	A	0.20	7.30×10^{-9}	-0.006	0.001	rs797267	0.99	*DUSP6*	intronic	13.15	NA
