# Supplementary material for: Shared genetic architecture of hernias: A genome-wide association study with multivariable meta-analysis of multiple hernia phenotypes
Source: PLoS One. 2022 Dec 30;17(12):e0272261. doi: 10.1371/journal.pone.0272261 (PMC9803250; doi:10.1371/journal.pone.0272261)
Supplement: S16 Table — 18 genome-wide significant exonic SNPs associated with umbrella hernia that were identified by FUMA SNP2GENE. All exonic SNPs are in high linkage with the index SNP at each locus (r2 > 0.6). Non-synonymous missense SNPs predicted to be damaging and deleterious by PolyPhen and SIFT are highlighted in blue. (PDF) [file pone.0272261.s016.pdf]

S1 Table 16. Umbrella hernia associated exonic variants. 18 genome-wide significant exonic SNPs associated with umbrella hernia that were identified by FUMA SNP2GENE. All exonic SNPs are in high linkage with the index SNP at each locus ($r^2 > 0.6$). Non-synonymous missense SNPs predicted to be damaging and deleterious by PolyPhen and SIFT are highlighted in blue.

rsID	Chr	Position	A1	A2	A1 Freq	P-value	BETA	SE	Index SNP	r2	Nearest Gene	CADD	RDB	Functionality	HGVSp	GERP	Polyphen	SIFT
rs41266839	6	26409890	G	C	0.07	2.80×10^{-16}	0.0111	0.0014	rs28360634	0.82	*BTN3A1*	0.504	4	Missense	p.Arg282Thr	-2.69	Benign	Tolerated
**rs13195401**	**6**	**26463574**	**G**	**T**	**0.07**	3.80×10^{-16}	**0.0111**	**0.0014**	**rs28360634**	**0.88**	***BTN2A1***	**22.8**	**5**	**Missense**	**p.Trp178Leu**	**2.25**	**Probably damaging**	**deleterious**
**rs13195402**	**6**	**26463575**	**G**	**T**	**0.07**	1.30×10^{-15}	**0.0108**	**0.0014**	**rs28360634**	**0.88**	***BTN2A1***	**23.7**	**5**	**Missense**	**p.Trp178Cys**	**2.25**	**Probably damaging**	**deleterious**
rs13195509	6	26463660	G	A	0.08	1.60×10^{-14}	0.0100	0.0013	rs28360634	0.76	*BTN2A1*	22.5	1f	Missense	p.Val207Met	-0.76	Possibly damaging	tolerated
rs3734542	6	26468326	G	A	0.08	1.10×10^{-14}	0.0101	0.0013	rs28360634	0.76	*BTN2A1*	5.384	5	Missense	p.Arg378Gln	-4.47	Benign	Tolerated
rs3734543	6	26468545	G	C	0.08	1.10×10^{-14}	0.0101	0.0013	rs28360634	0.76	*BTN2A1*	11.89	5	Missense	p.Gly451Ala	-2.24	Benign	Tolerated
rs35555795	6	26509382	C	T	0.08	1.00×10^{-14}	0.0101	0.0013	rs28360634	0.77	*BTN1A1*	0.919	7	Missense	p.Pro521Ser	-4.7	Benign	Tolerated
rs139332558	6	26637724	T	C	0.07	4.40×10^{-16}	0.0114	0.0014	rs28360634	0.89	*ZNF322*	19.82	5	Missense	-	-0.09	Benign	Tolerated
rs200484	6	27775674	A	G	0.09	2.30×10^{-14}	0.0094	0.0012	rs28360634	0.81	*HIST1H2BL*	16.23	1f	Missense	p.Leu4Pro	2.28	Benign	Tolerated (low confidence)
rs200981	6	27833174	A	G	0.09	2.20×10^{-14}	0.0095	0.0012	rs28360634	0.78	*HIST1H2AL*	15.9	NA	Synonymous	-	-	-	-
rs17763089	6	27835218	G	A	0.07	1.90×10^{-15}	0.0104	0.0013	rs28360634	0.96	*HIST1H1B*	7.407	4	Synonymous	-	-	-	-
rs200948	6	27835272	T	C	0.09	2.10×10^{-14}	0.0095	0.0012	rs28360634	0.80	*HIST1H1B*	12.12	1f	Synonymous		-		
**rs34788973**	**6**	**27879200**	**C**	**A**	**0.07**	4.80×10^{-17}	**0.0112**	**0.0013**	**rs28360634**	**0.95**	***OR2B2***	**23.2**	**7**	**Missense**	**p.Ala300Ser**	**2.28**	**Possibly damaging**	**Deleterious**
**rs61742093**	**6**	**27879982**	**A**	**G**	**0.07**	4.70×10^{-16}	**0.0108**	**0.0013**	**rs28360634**	**0.95**	***OR2B2***	**22.6**	**3a**	**Missense**	**p.Ile39Thr**	**2.37**	**Possibly damaging**	**Deleterious**
rs33932084	6	28268824	A	G	0.07	1.20×10^{-15}	0.0106	0.0013	rs28360634	0.85	*PGBD1*	14.75	3a	Missense	p.Asn398Ser	-1.27	Benign	Tolerated
rs17855988	7	73474825	G	C	0.09	3.80×10^{-12}	0.0101	0.0015	rs17855988	1.00	*ELN:CTB-51J22.1*	25.9	2b	Missense	p.Gly581Arg	3.75	Unknown	Deleterious (low confidence)
rs10866845	8	25708267	T	C	0.45	1.80×10^{-18}	-0.0076	0.0009	rs4368985	0.93	*EBF2*	0.004	7	Synonymous	-	-	-	-
rs770087	12	89744773	A	C	0.20	6.70×10^{-9}	-0.0063	0.0011	rs797267	0.99	*DUSP6*	23	NA	Missense	p.Ser144Ala	-1.48	Benign	Tolerated
