# Supplementary material for: Shared genetic architecture of hernias: A genome-wide association study with multivariable meta-analysis of multiple hernia phenotypes
Source: PLoS One. 2022 Dec 30;17(12):e0272261. doi: 10.1371/journal.pone.0272261 (PMC9803250; doi:10.1371/journal.pone.0272261)
Supplement: S17 Table — The convergence of 15,496 gene sets (15,381from MSigDB v7.0) were tested. A Bonferroni-corrected threshold of P < 3.23×10^–6^ (0.05/15,496) was set, resulting in 21 significant Gene Ontology (GO) gene sets and three curated gene sets. This analysis was performed using the SNP2GENE tool in FUMA. (PDF) [file pone.0272261.s017.pdf]

S1 Table 17. Enriched gene sets from the genome-wide gene-based enrichment analysis of overlap hernia in MAGMA v1.07. The convergence of 15,496 gene sets (15,381from MSigDB v7.0) were tested. A Bonferroni-corrected threshold of $P < 3.23\times10^{-6}$ (0.05/15,496) was set, resulting in 21 significant Gene Ontology (GO) gene sets and three curated gene sets. This analysis was performed using the SNP2GENE tool in FUMA.

Gene Set	Number of genes	Beta	Beta S.D.	SE	P-value
GO_bp:go_pericardium_development	20	1.3046	0.0424	0.2401	2.81×10^{-8}
Curated_gene_sets:wong_endmetrium_cancer_dn	71	0.6319	0.0386	0.1167	3.14×10^{-8}
GO_bp:go_endocrine_system_development	127	0.4471	0.0365	0.0828	3.43×10^{-8}
GO_bp:go_positive_regulation_of_transcription_by_rna_polymerase_ii	1124	0.1492	0.0353	0.028	4.79×10^{-8}
GO_bp:go_blastoderm_segmentation	19	1.1606	0.0368	0.2231	9.93×10^{-8}
GO_bp:go_negative_regulation_of_cell_proliferation_involved_in_kidney_development	5	2.514	0.0409	0.4863	1.19×10^{-7}
Curated_gene_sets:reactome_elastic_fibre_formation	46	0.6941	0.0342	0.1362	1.74×10^{-7}
GO_bp:go_gland_development	424	0.2225	0.0329	0.0445	2.87×10^{-7}
GO_bp:go_aortic_valve_development	28	0.9251	0.0356	0.1852	2.99×10^{-7}
Curated_gene_sets:reactome_molecules_associated_with_elastic_fibres	39	0.7278	0.033	0.1464	3.38×10^{-7}
GO_bp:go_mesenchyme_development	256	0.2876	0.0332	0.058	3.61×10^{-7}
GO_bp:go_heart_development	526	0.2011	0.0331	0.0412	5.21×10^{-7}
GO_bp:go_connective_tissue_development	262	0.2772	0.0324	0.0568	5.45×10^{-7}
GO_bp:go_outflow_tract_morphogenesis	76	0.548	0.0347	0.1126	5.73×10^{-7}
GO_mf:go_dna_binding_transcription_activator_activity	398	0.2324	0.0333	0.0479	6.06×10^{-7}
GO_bp:go_olfactory_bulb_interneuron_differentiation	11	1.4441	0.0348	0.302	8.74×10^{-7}

GO_bp:go_semi_lunar_valve_development	36	0.7671	0.0334	0.161	9.51×10^{-7}
GO_bp:go_tripartite_regional_subdivision	15	1.2359	0.0348	0.2614	1.14×10^{-6}
GO_bp:go_animal_organ_morphogenesis	1005	0.1431	0.0321	0.0304	1.26×10^{-6}
GO_bp:go_positive_regulation_of_gene_expression	1838	0.1015	0.0301	0.0217	1.52×10^{-6}
GO_bp:go_regionalization	331	0.2555	0.0335	0.0552	1.87×10^{-6}
GO_mf:go_regulatory_region_nucleic_acid_binding	890	0.1441	0.0305	0.0313	2.11×10^{-6}
GO_bp:go_pattern_specification_process	420	0.2191	0.0323	0.0483	2.95×10^{-6}
GO_bp:go_negative_regulation_of_metanephros_development	8	1.6631	0.0342	0.3685	3.21×10^{-6}
