# Supplementary material for: Shared genetic architecture of hernias: A genome-wide association study with multivariable meta-analysis of multiple hernia phenotypes
Source: PLoS One. 2022 Dec 30;17(12):e0272261. doi: 10.1371/journal.pone.0272261 (PMC9803250; doi:10.1371/journal.pone.0272261)
Supplement: S18 Table — (PDF) [file pone.0272261.s018.pdf]

S1 Table 18. Gene-based enrichment analysis for overlap hernia associated genes in eXploring Genomic Relations (XGR).

Term Name	Z-score	P-value	FDR	Overlapping genes	Genes
Regulation of Telomerase	4.88	0.00038	0.0013	1	WT1
Genes encoding structural ECM glycoproteins	2.7	0.0032	0.0054	1	EFEMP1
