# Supplementary material for: Shared genetic architecture of hernias: A genome-wide association study with multivariable meta-analysis of multiple hernia phenotypes
Source: PLoS One. 2022 Dec 30;17(12):e0272261. doi: 10.1371/journal.pone.0272261 (PMC9803250; doi:10.1371/journal.pone.0272261)
Supplement: S19 Table — The convergence of 15,496 gene sets (15,381from MSigDB v7.0) were tested. A Bonferroni-corrected threshold of P < 3.23×10^–6^ (0.05/15,496) was set, resulting in two significant Gene Ontology (GO) gene sets. This analysis was performed using the SNP2GENE tool in FUMA. (PDF) [file pone.0272261.s019.pdf]

**S1 Table 19. Enriched gene sets from the genome-wide gene-based enrichment analysis of umbrella hernia in MAGMA v1.07.** The convergence of 15,496 gene sets (15,381from MSigDB v7.0) were tested. A Bonferroni-corrected threshold of P < $3.23\times10^{-6}$ (0.05/15,496) was set, resulting in two significant Gene Ontology (GO) gene sets. This analysis was performed using the SNP2GENE tool in FUMA.

| Gene Set | N genes | Beta | SE | SE | P |
|---|---|---|---|---|---|
| GO_bp:go_negative_regulation_of_cell_proliferation_involved_in_kidney_development | 5 | 2.14 | 0.035 | 0.44 | $5.76\times10^{-7}$ |
| GO_bp:go_diaphragm_development | 9 | 1.32 | 0.029 | 0.29 | $3.00\times10^{-6}$ |
