# Supplementary material for: Shared genetic architecture of hernias: A genome-wide association study with multivariable meta-analysis of multiple hernia phenotypes
Source: PLoS One. 2022 Dec 30;17(12):e0272261. doi: 10.1371/journal.pone.0272261 (PMC9803250; doi:10.1371/journal.pone.0272261)
Supplement: S20 Table — Hernia cases were defined if they had a diagnostic (ICD-10 or self-report) and/or operative (OPCS4 or self-report) code for either inguinal, femoral, umbilical or hiatus hernia. Overlapping hernia cases were removed to construct four cohorts of individual hernia cases. (PDF) [file pone.0272261.s020.pdf]

S1 Table 20. Phenotype codes used for four individual hernia case definitions. Hernia cases were defined if they had a diagnostic (ICD-10 or self-report) and/or operative (OPCS4 or self-report) code for either inguinal, femoral, umbilical or hiatus hernia. Overlapping hernia cases were removed to construct four cohorts of individual hernia cases.

Inguinal hernia

Source of Data	UK Biobank Data Field	Code	Description	N
Primary ICD-10	41202	K40	Inguinal hernia	20,321
Secondary ICD-10	41204	As above	As above	1,243
Primary OPCS	41200	T19<br>T20<br>T21	Simple excision of inguinal hernia sac<br>Primary repair of inguinal hernia<br>Repair of recurrent inguinal hernia	20,493
Secondary OPCS	41210	As above	As above	582
Non-cancer illness (self-report)	20002	1513	Inguinal hernia	3,279
Operation (self-report)	20004	1563	Inguinal hernia repair	9,828
Total unique cases (Pre QC)				27,695
Total unique cases (Post QC)				23,007
**Unique cases with no overlap with other hernia phenotypes**				**18,791**

Femoral hernia

Source of Data	UK Biobank Data Field	Code	Description	N
Primary ICD-10	41202	K41	Femoral hernia	766

Secondary ICD-10	41204	As above	As above	125
Primary OPCS	41200	T22<br>T23	Primary repair of femoral hernia<br>Repair of recurrent femoral hernia	757
Secondary OPCS	41210	As above	As above	95
Non-cancer illness (self-report)	20002	1605	Femoral hernia	407
Operation (self-report)	20004	1564	Femoral hernia repair	764
Total unique cases (Pre QC)				1,897
Total unique cases (Post QC)				1,578
**Unique cases with no overlap with other hernia phenotypes**				**973**

Umbilical hernia

Source of Data	UK Biobank Data Field	Code	Description	N
Primary ICD-10	41202	K42	Umbilical hernia	4,117
Secondary ICD-10	41204	As above	As above	1,486
Primary OPCS	41200	T24<br>T97	Primary repair of umbilical hernia<br>Repair of recurrent umbilical hernia	4,013
Secondary OPCS	41210	As above	As above	858
Non-cancer illness (self-report)	20002	1512	Umbilical hernia	887
Operation (self-report)	20004	1404	Umbilical hernia repair	4,959
Total unique cases (Pre QC)				9,289

Total unique cases (Post QC)				7,432
**Unique cases with no overlap with other hernia phenotypes**				**5,356**

Hiatus hernia

Source of Data	UK Biobank Data Field	Code	Description	N
Primary ICD-10	41202	K44	Diaphragmatic hernia	13,758
Secondary ICD-10	41204	As above	As above	30,780
Primary OPCS	41200	G23	Repair of diaphragmatic hernia	317
Secondary OPCS	41210	As above	As above	172
Non-cancer illness (self-report)	20002	1474	Hiatus hernia	12,387
Operation (self-report)	20004	1482	Oesphageal fundoplication/hiatus hernia surgery	1,531
Total unique cases (Pre QC)				44,327
Total unique cases (Post QC)				36,139
**Unique cases with no overlap with other hernia phenotypes**				**31,543**
