# Supplementary material for: Shared genetic architecture of hernias: A genome-wide association study with multivariable meta-analysis of multiple hernia phenotypes
Source: PLoS One. 2022 Dec 30;17(12):e0272261. doi: 10.1371/journal.pone.0272261 (PMC9803250; doi:10.1371/journal.pone.0272261)

S2 SUPPLEMENTARY FIGURES

S2 Fig 1. Manhattan plots for the four individual hernia analyses in UK Biobank. Manhattan plots are annotated with the gene names of loci that demonstrate shared susceptibility across two or more individual analyses. The 6q24.2 (*AIG1*) locus is plotted for inguinal hernia because it shows shared susceptibility with the overlap hernia analysis. *ZC3H11B* is shown as a putative gene at 1q41 for femoral hernia as it was mapped in the joint analysis in metaUSAT.

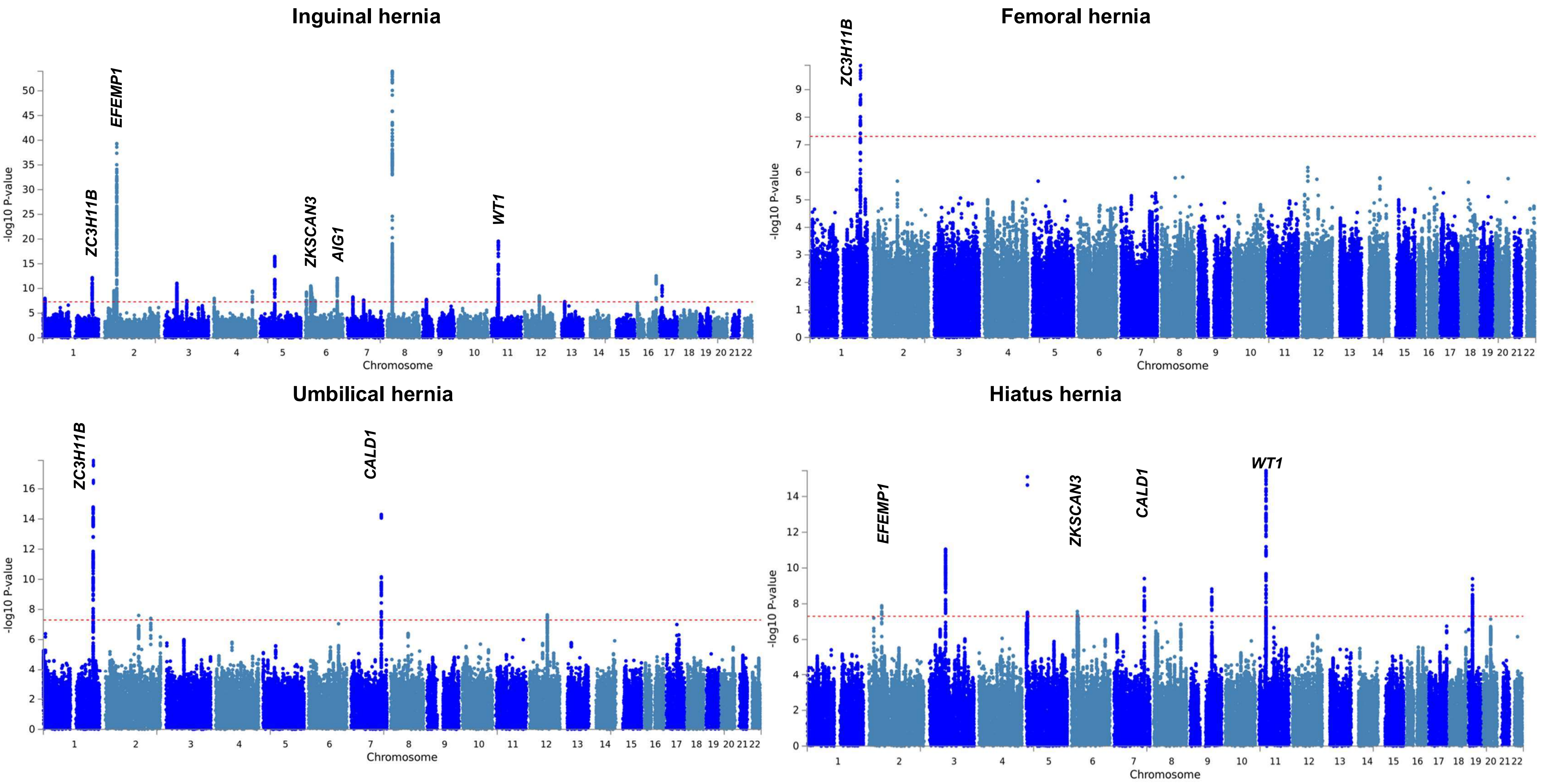

Supplement: S1 Fig — Manhattan plots are annotated with the gene names of loci that demonstrate shared susceptibility across two or more individual analyses. The 6q24.2 (AIG1) locus is plotted for inguinal hernia because it shows shared susceptibility with the overlap hernia analysis. ZC3H11B is shown as a putative gene at 1q41 for femoral hernia as it was mapped in the joint analysis in metaUSAT. (PDF) [file pone.0272261.s021.pdf]
