# Supplementary material for: Shared genetic architecture of hernias: A genome-wide association study with multivariable meta-analysis of multiple hernia phenotypes
Source: PLoS One. 2022 Dec 30;17(12):e0272261. doi: 10.1371/journal.pone.0272261 (PMC9803250; doi:10.1371/journal.pone.0272261)

S2 Figure 2. Quantile-quantile (Q-Q) plots for all four individual hernia analyses. A: Inguinal hernia analysis. B: Femoral hernia analysis. C: Umbilical hernia analysis. D: Hiatus hernia analysis. The λ_{GC} demonstrated nominal inflation levels across the four association analyses, ranging from 1.00-1.20 (λ_{GC}-femoral: 1.00; λ_{GC}-umbilical: 1.05; λ_{GC}-inguinal: 1.15; λ_{GC}-hiatus: 1.20), however the LDSC intercept range of 1.00-1.03 (Femoral: 1.00; Umbilical: 1.01; Inguinal: 1.02; Hiatus: 1.03) and an attenuation ratio of 0.08-0.19 (Umbilical: 0.08; Inguinal:0.11; Hiatus: 0.13; Femoral: 0.19) is fully in keeping with the effects of polygenicity and large sample size.

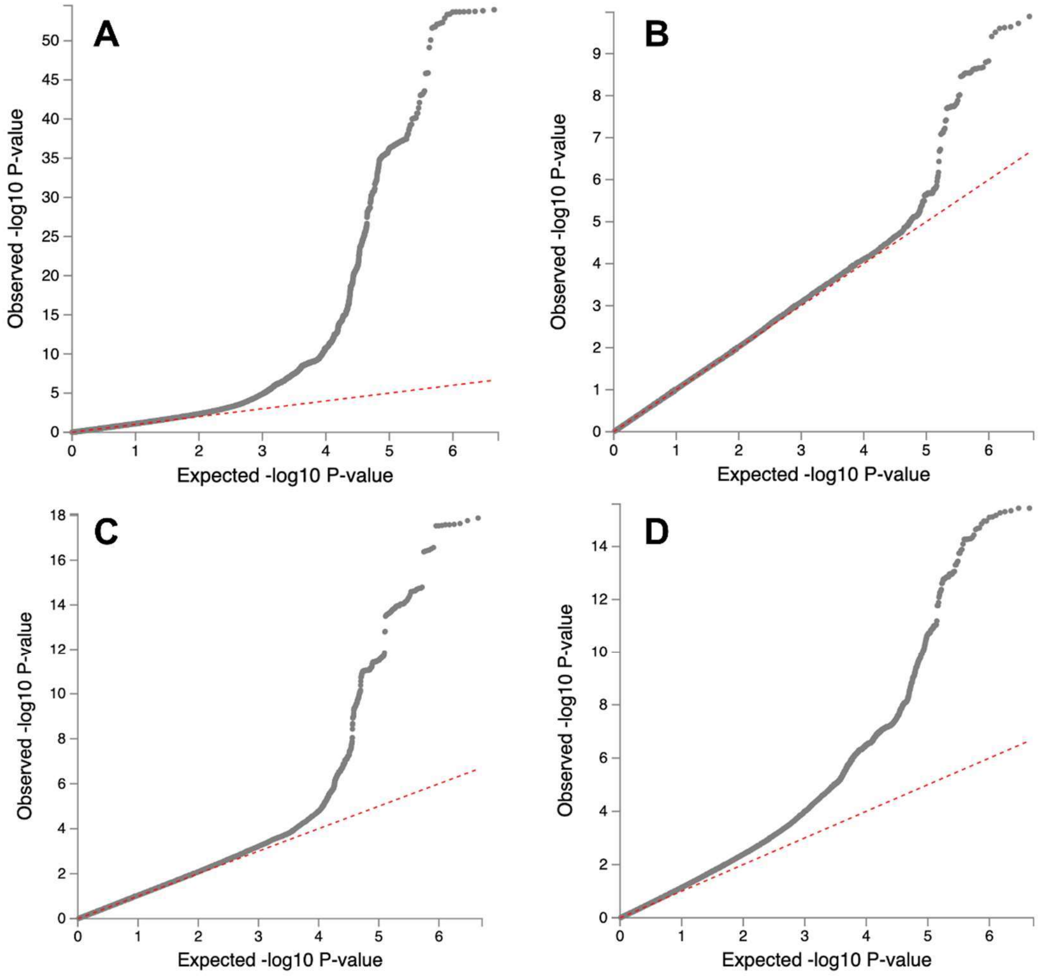

Supplement: S2 Fig — A: Inguinal hernia analysis. B: Femoral hernia analysis. C: Umbilical hernia analysis. D: Hiatus hernia analysis. The λGC demonstrated nominal inflation levels across the four association analyses, ranging from 1.00–1.20 (λGC-femoral: 1.00; λGC-umbilical: 1.05; λGC-inguinal: 1.15; λGC-hiatus: 1.20), however the LDSC intercept range of 1.00–1.03 (Femoral: 1.00; Umbilical: 1.01; Inguinal: 1.02; Hiatus: 1.03) and an attenuation ratio of 0.08–0.19 (Umbilical: 0.08; Inguinal: 0.11; Hiatus: 0.13; Femoral: 0.19) is fully in keeping with the effects of polygenicity and large sample size. (PDF) [file pone.0272261.s022.pdf]
