# Supplementary material for: Shared genetic architecture of hernias: A genome-wide association study with multivariable meta-analysis of multiple hernia phenotypes
Source: PLoS One. 2022 Dec 30;17(12):e0272261. doi: 10.1371/journal.pone.0272261 (PMC9803250; doi:10.1371/journal.pone.0272261)
Supplement: S3 Fig — LocusZoom plots of the 28 inguinal, 1 femoral, 5 umbilical and 8 hiatus hernia independent genome-wide significant associated signals. Plots are ordered by chromosome number and genomic position. SNP position is shown on the x-axis, and strength of association on the y-axis (-log10 P-value). The linkage disequilibrium (LD) relationship between the lead SNP and the surrounding SNPs is indicated by the r2 legend. In the lower panel of each figure, genes within 500kb of the index SNP are shown. The position on each chromosome is shown in relation to Human Genome build hg19. (PDF) [file pone.0272261.s023.pdf]

S2 Fig 3. Regional Locus Zoom plots for all four Individual hernia associated signals. LocusZoom plots of the 28 inguinal, 1 femoral, 5 umbilical and 8 hiatus hernia independent genome-wide significant associated signals. Plots are ordered by chromosome number and genomic position. SNP position is shown on the x-axis, and strength of association on the y-axis (-log10 P-value). The linkage disequilibrium (LD) relationship between the lead SNP and the surrounding SNPs is indicated by the r^2 legend. In the lower panel of each figure, genes within 500kb of the index SNP are shown. The position on each chromosome is shown in relation to Human Genome build hg19.

Inguinal Hernia

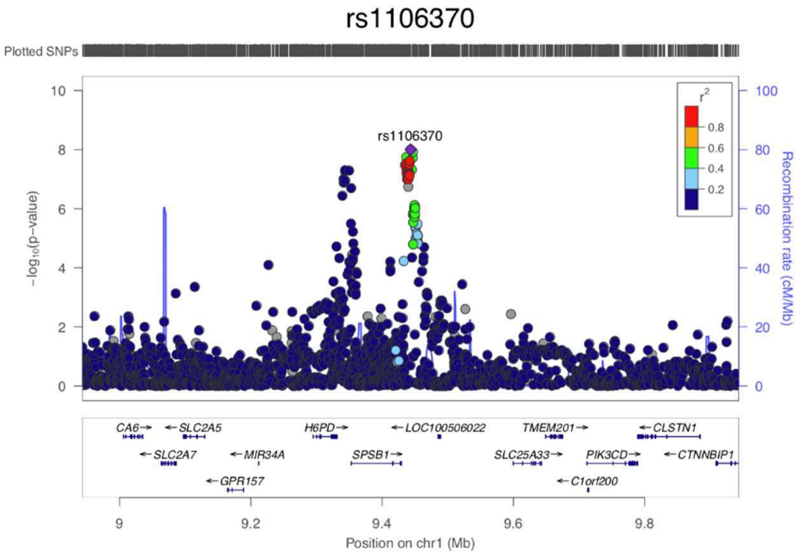

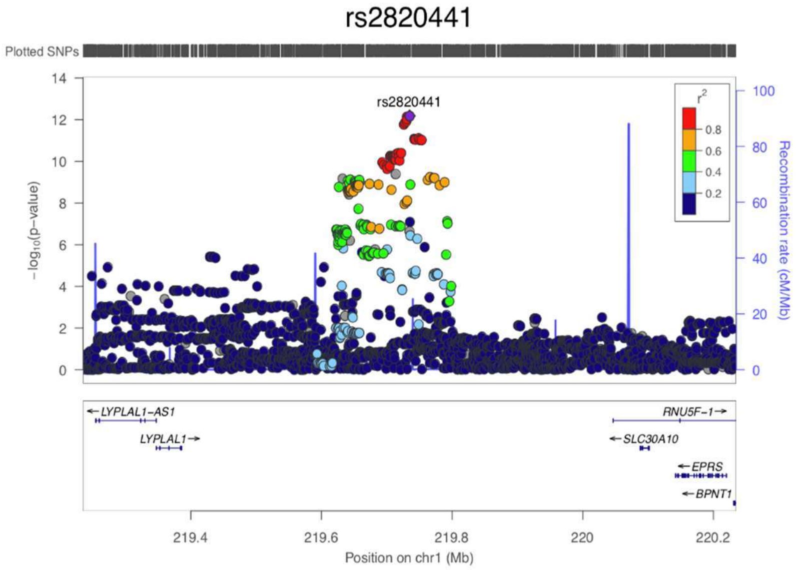

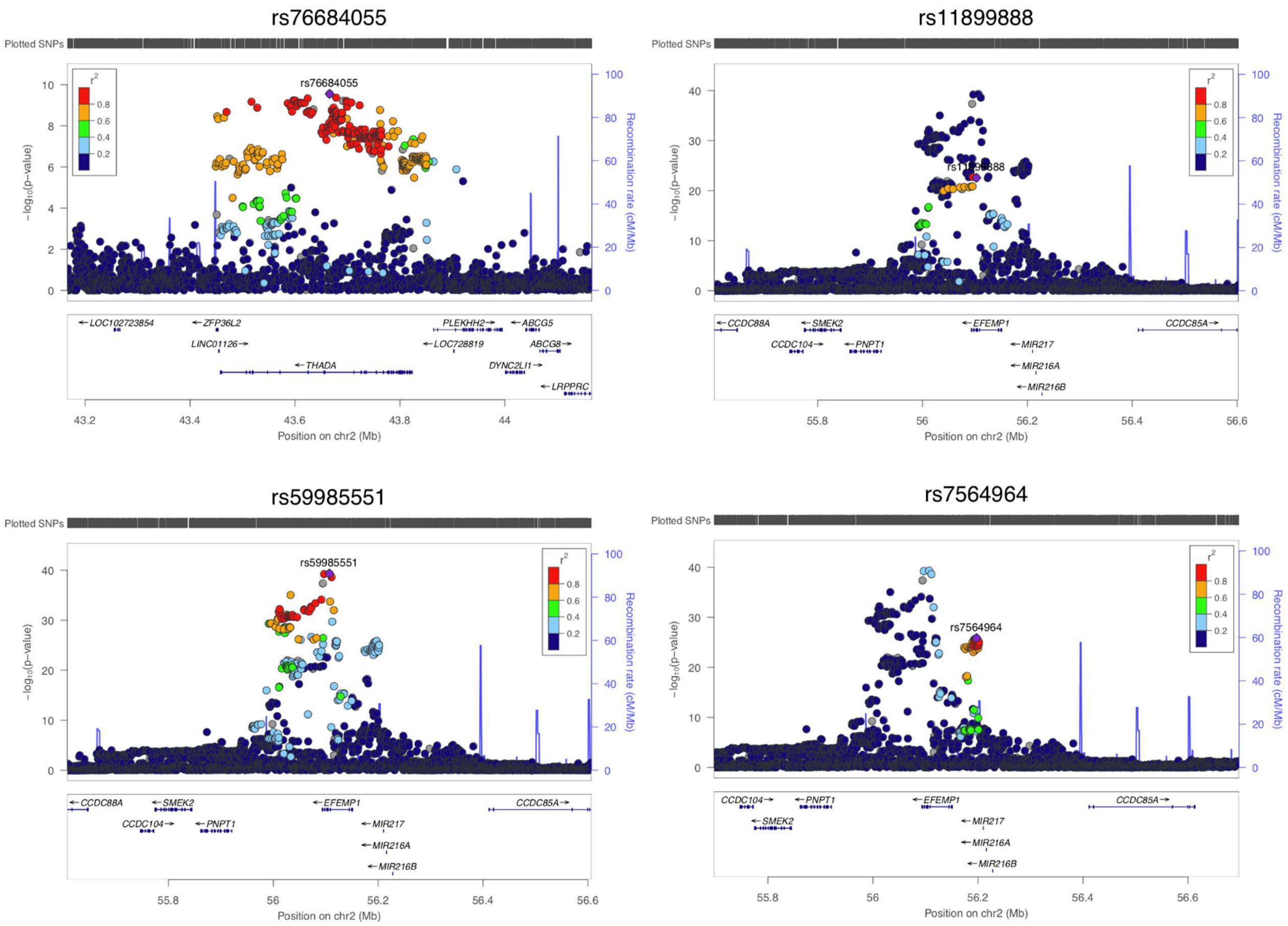
rs76684055
Plotted SNPs
rs76684055
−log10(p−value)
Recombination rate (cM/Mb)
LOC102723854
ZFP36L2
LINC01126
THADA
PLEKHH2
ABCG5
LOC728819
ABCG8
DYNC2LI1
LRPPRC
Position on chr2 (Mb)
rs11899888
Plotted SNPs
rs11899888
CCDC88A
SMEK2
CCDC104
PNPT1
EFEMP1
MIR217
MIR216A
MIR216B
CCDC85A
Position on chr2 (Mb)
rs59985551
Plotted SNPs
rs59985551
CCDC88A
SMEK2
CCDC104
PNPT1
EFEMP1
MIR217
MIR216A
MIR216B
CCDC85A
Position on chr2 (Mb)
rs7564964
Plotted SNPs
rs7564964
CCDC104
PNPT1
SMEK2
EFEMP1
MIR217
MIR216A
MIR216B
CCDC85A
Position on chr2 (Mb)

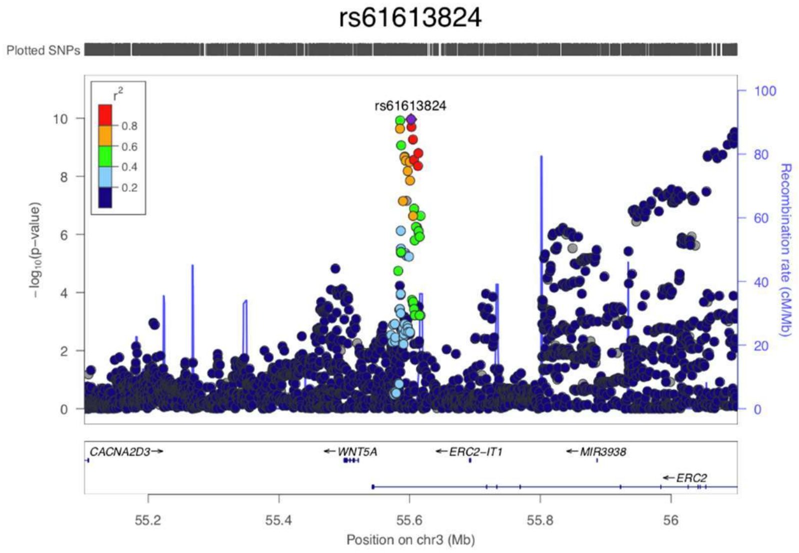
rs61613824
Plotted SNPs
rs61613824
r2
0.8
0.6
0.4
0.2
−log10(p−value)
Recombination rate (cM/Mb)
CACNA2D3→
←WNT5A
←ERC2−IT1
←MIR3938
←ERC2
55.2
55.4
55.6
55.8
56
Position on chr3 (Mb)

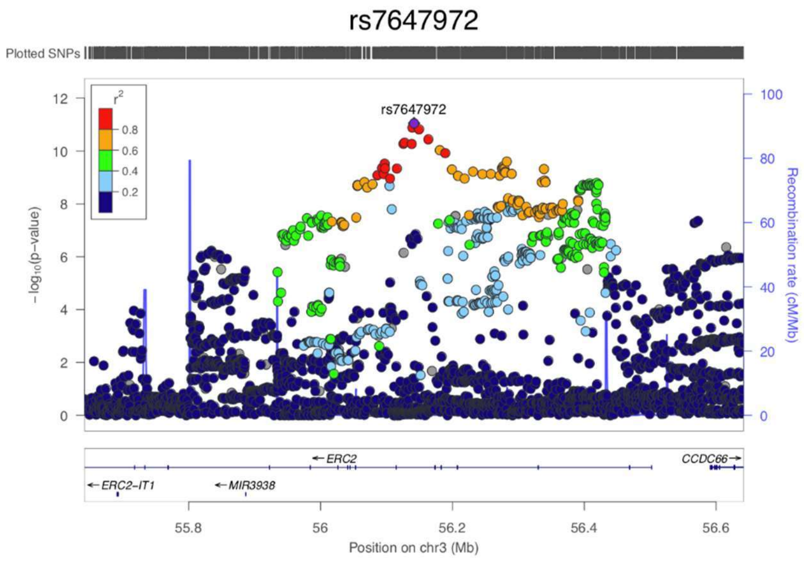
rs7647972
Plotted SNPs
rs7647972
r2
0.8
0.6
0.4
0.2
−log10(p−value)
Recombination rate (cM/Mb)
←ERC2
CCDC66→
←ERC2−IT1
←MIR3938
55.8
56
56.2
56.4
56.6
Position on chr3 (Mb)

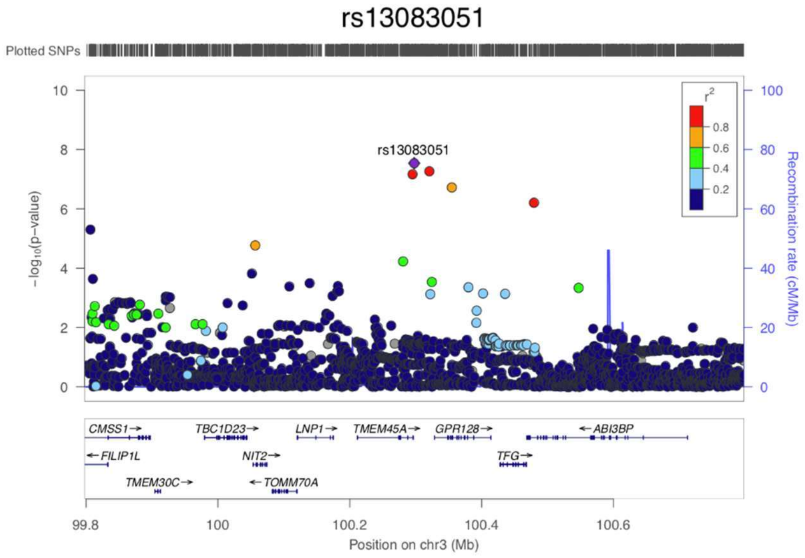
rs13083051
Plotted SNPs
rs13083051
r2
0.8
0.6
0.4
0.2
−log10(p−value)
Recombination rate (cM/Mb)
CMSS1→
TBC1D23→
LNP1→
TMEM45A→
GPR128→
←ABI3BP
←FILIP1L
NIT2→
TFG→
TMEM30C→
←TOMM70A
99.8
100
100.2
100.4
100.6
Position on chr3 (Mb)

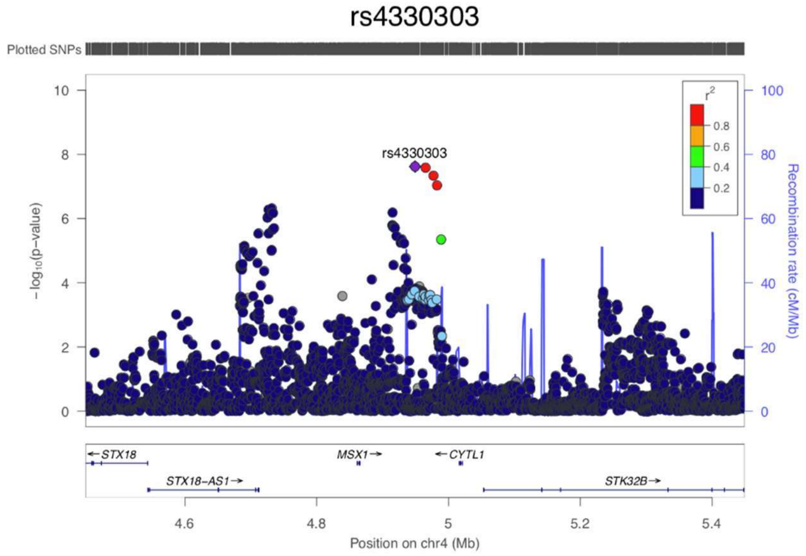
rs4330303
Plotted SNPs
rs4330303
r2
0.8
0.6
0.4
0.2
−log10(p−value)
Recombination rate (cM/Mb)
←STX18
MSX1→
←CYTL1
STX18−AS1→
STK32B→
4.6
4.8
5
5.2
5.4
Position on chr4 (Mb)

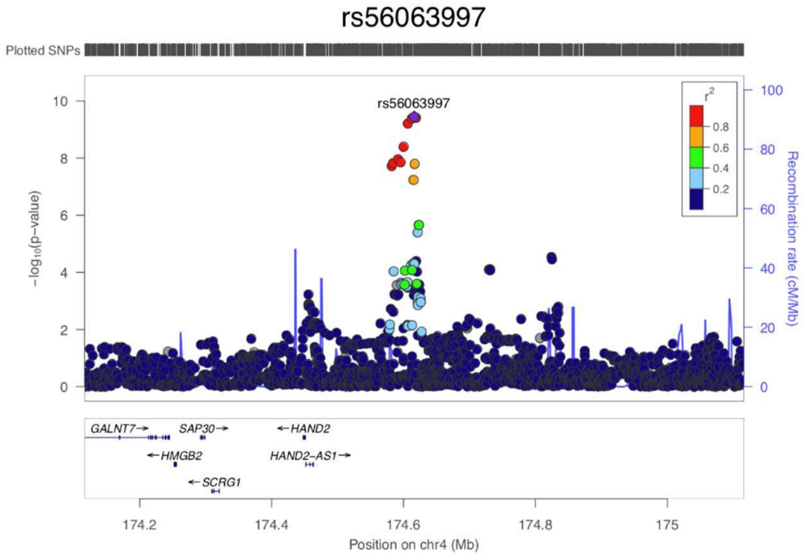
rs56063997
Plotted SNPs
rs56063997
r²
0.8
0.6
0.4
0.2
−log10(p−value)
Recombination rate (cM/Mb)
GALNT7
SAP30
HAND2
HMGB2
HAND2−AS1
SCRG1
174.2
174.4
174.6
174.8
175
Position on chr4 (Mb)

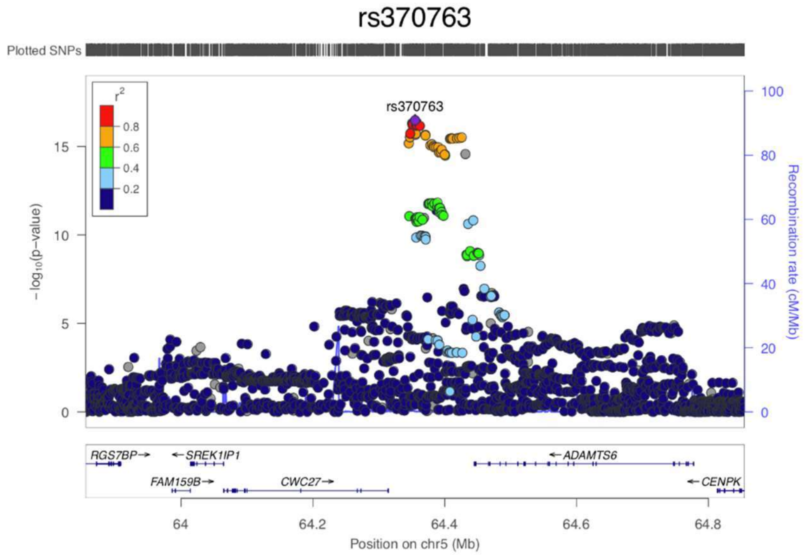
rs370763
Plotted SNPs
rs370763
r²
0.8
0.6
0.4
0.2
−log10(p−value)
Recombination rate (cM/Mb)
RGS7BP
SREK1IP1
ADAMTS6
FAM159B
CWC27
CENPK
64
64.2
64.4
64.6
64.8
Position on chr5 (Mb)

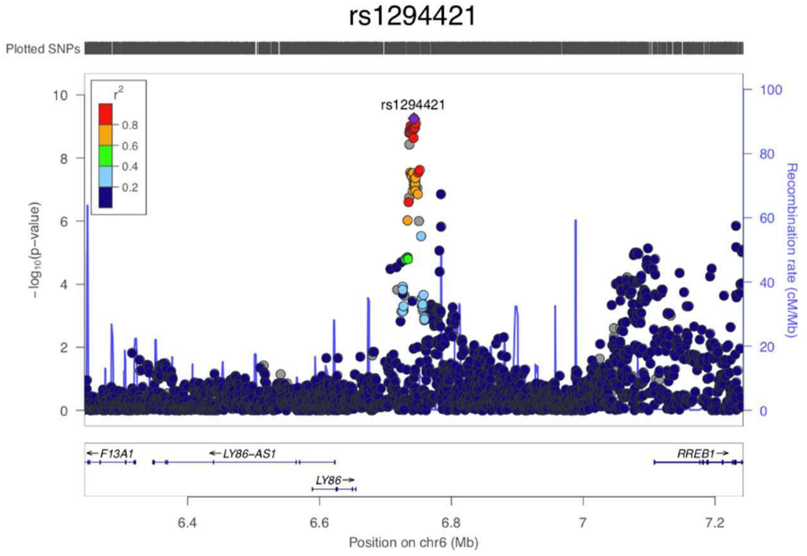
rs1294421
Plotted SNPs
rs1294421
r²
0.8
0.6
0.4
0.2
−log10(p−value)
Recombination rate (cM/Mb)
F13A1
LY86−AS1
RREB1
LY86
6.4
6.6
6.8
7
7.2
Position on chr6 (Mb)

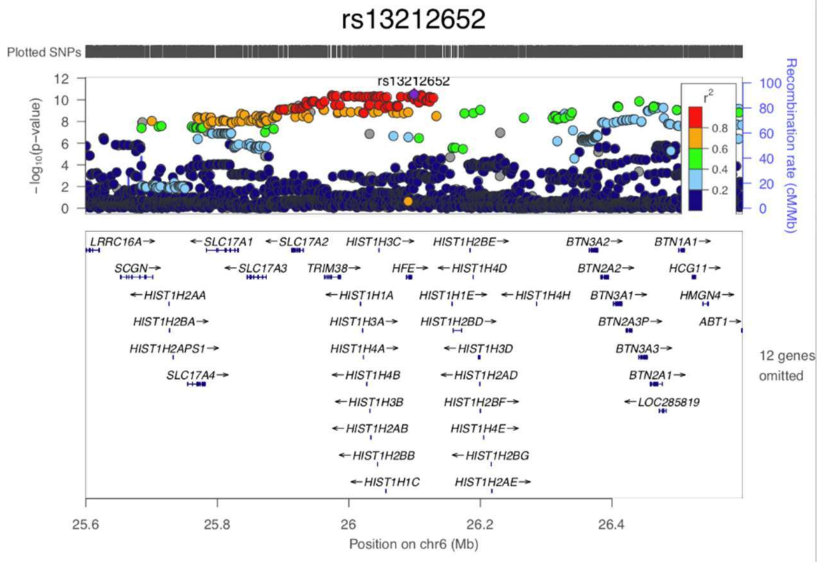
rs13212652
Plotted SNPs
rs13212652
r²
0.8
0.6
0.4
0.2
−log10(p−value)
Recombination rate (cM/Mb)
LRRC16A
SLC17A1
SLC17A2
HIST1H3C
HIST1H2BE
BTN3A2
BTN1A1
SCGN
SLC17A3
TRIM38
HFE
HIST1H4D
BTN2A2
HCG11
HIST1H2AA
HIST1H1A
HIST1H1E
HIST1H4H
BTN3A1
HMGN4
HIST1H2BA
HIST1H3A
HIST1H2BD
BTN2A3P
ABT1
HIST1H2APS1
HIST1H4A
HIST1H3D
BTN3A3
SLC17A4
HIST1H4B
HIST1H2AD
BTN2A1
HIST1H3B
HIST1H2BF
LOC285819
HIST1H2AB
HIST1H4E
HIST1H2BB
HIST1H2BG
HIST1H1C
HIST1H2AE
12 genes omitted
25.6
25.8
26
26.2
26.4
Position on chr6 (Mb)

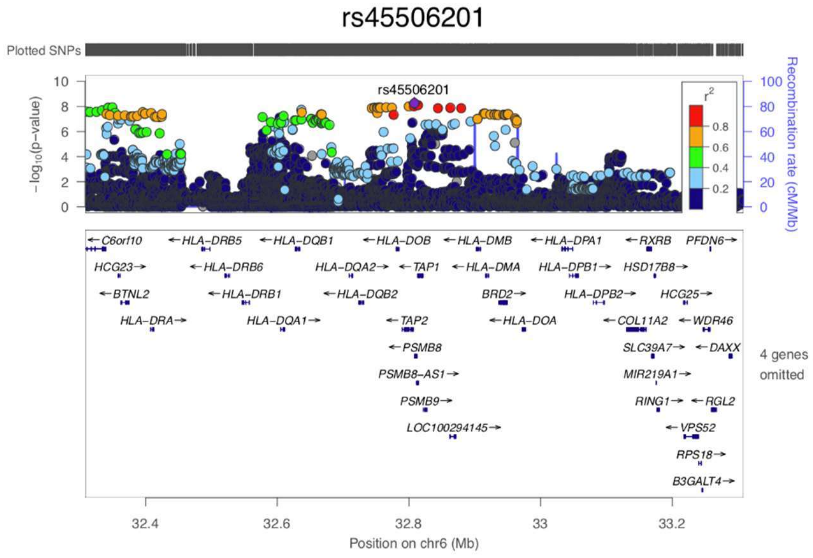
rs45506201
Plotted SNPs
-log10(p-value)
Recombination rate (cM/Mb)
r2
rs45506201
C6orf10
HLA-DRB5
HLA-DQB1
HLA-DOB
HLA-DMB
HLA-DPA1
RXRB
PFDN6
HCG23
HLA-DRB6
HLA-DQA2
TAP1
HLA-DMA
HLA-DPB1
HSD17B8
BTNL2
HLA-DRB1
HLA-DQB2
BRD2
HLA-DPB2
HCG25
HLA-DRA
HLA-DQA1
TAP2
HLA-DOA
COL11A2
WDR46
PSMB8
SLC39A7
DAXX
PSMB8-AS1
MIR219A1
PSMB9
RING1
RGL2
LOC100294145
VPS52
RPS18
B3GALT4
4 genes omitted
Position on chr6 (Mb)

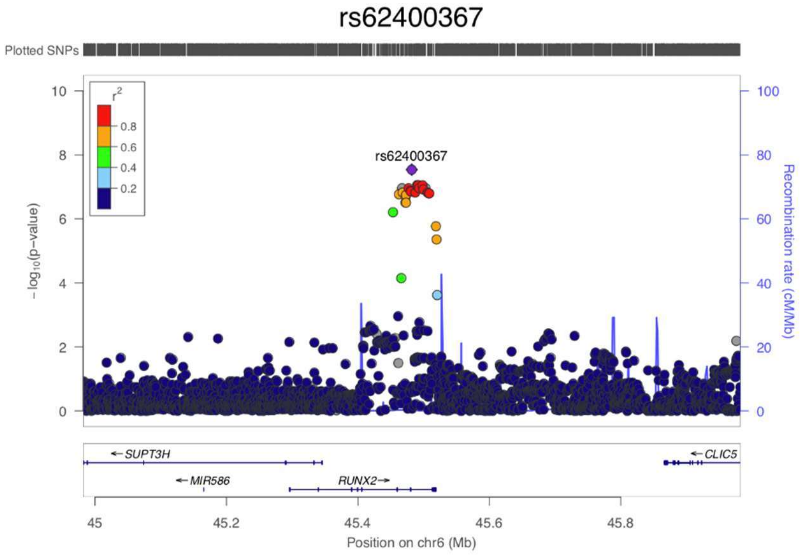
rs62400367
Plotted SNPs
-log10(p-value)
Recombination rate (cM/Mb)
r2
rs62400367
SUPT3H
CLIC5
MIR586
RUNX2
Position on chr6 (Mb)

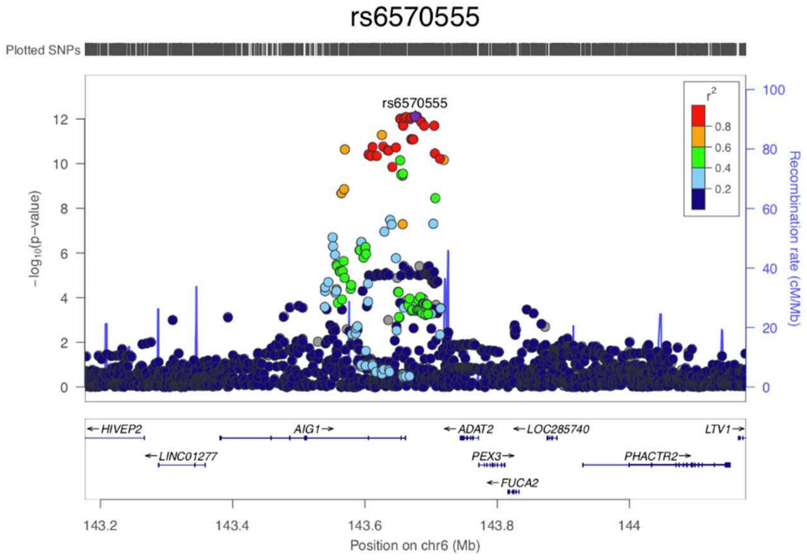
rs6570555
Plotted SNPs
-log10(p-value)
Recombination rate (cM/Mb)
r2
rs6570555
HIVEP2
AIG1
ADAT2
LOC285740
LTV1
LINC01277
PEX3
PHACTR2
FUCA2
Position on chr6 (Mb)

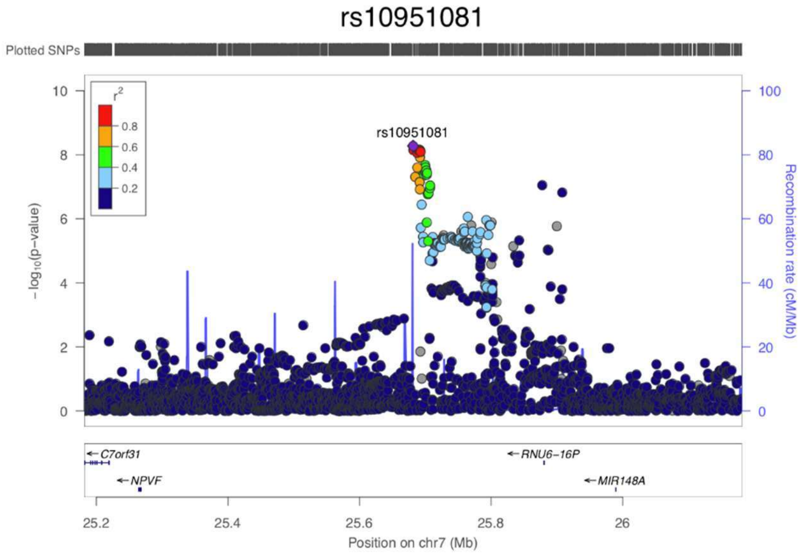
rs10951081
Plotted SNPs
-log10(p-value)
Recombination rate (cM/Mb)
r2
rs10951081
C7orf31
RNU6-16P
NPVF
MIR148A
Position on chr7 (Mb)

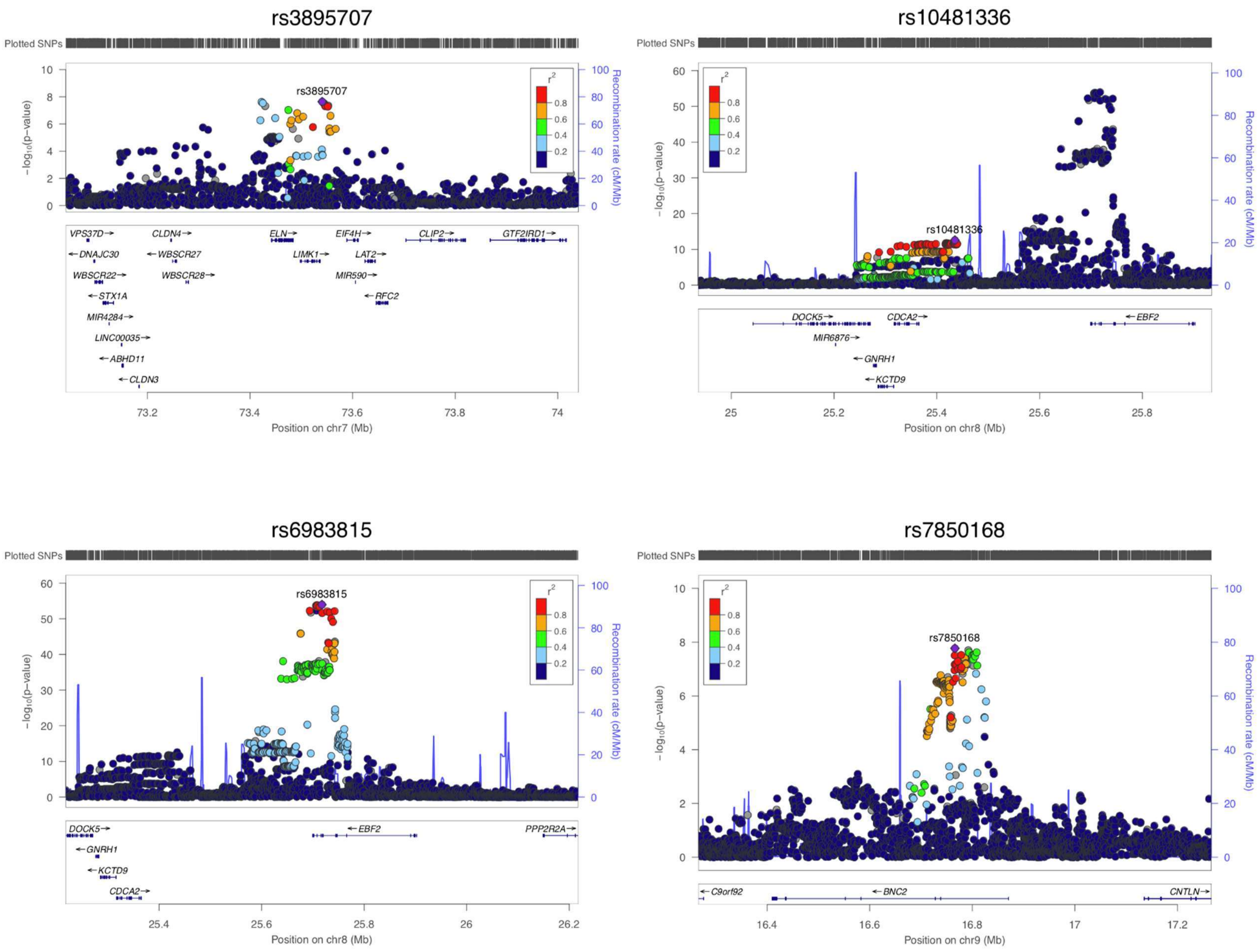
rs3895707
Plotted SNPs
−log10(p−value)
Recombination rate (cM/Mb)
rs3895707
r2
VPS37D→
←DNAJC30
WBSCR22→
←STX1A
MIR4284→
LINC00035→
←ABHD11
←CLDN3
CLDN4→
←WBSCR27
WBSCR28→
ELN→
LIMK1→
EIF4H→
LAT2→
MIR590→
←RFC2
CLIP2→
GTF2IRD1→
Position on chr7 (Mb)
rs10481336
Plotted SNPs
−log10(p−value)
Recombination rate (cM/Mb)
rs10481336
r2
DOCK5→
MIR6876→
←GNRH1
←KCTD9
CDCA2→
←EBF2
Position on chr8 (Mb)
rs6983815
Plotted SNPs
−log10(p−value)
Recombination rate (cM/Mb)
rs6983815
r2
DOCK5→
←GNRH1
←KCTD9
CDCA2→
←EBF2
PPP2R2A→
Position on chr8 (Mb)
rs7850168
Plotted SNPs
−log10(p−value)
Recombination rate (cM/Mb)
rs7850168
r2
←C9orf92
←BNC2
CNTLN→
Position on chr9 (Mb)

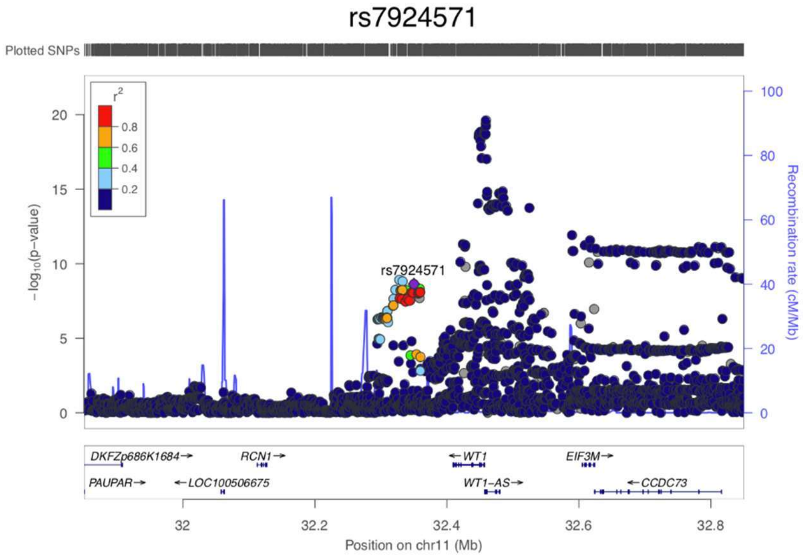
rs7924571
Plotted SNPs
rs7924571
−log10(p−value)
Recombination rate (cM/Mb)
DKFZp686K1684→
RCN1→
←WT1
EIF3M→
PAUPAR→
←LOC100506675
WT1−AS→
←CCDC73
Position on chr11 (Mb)

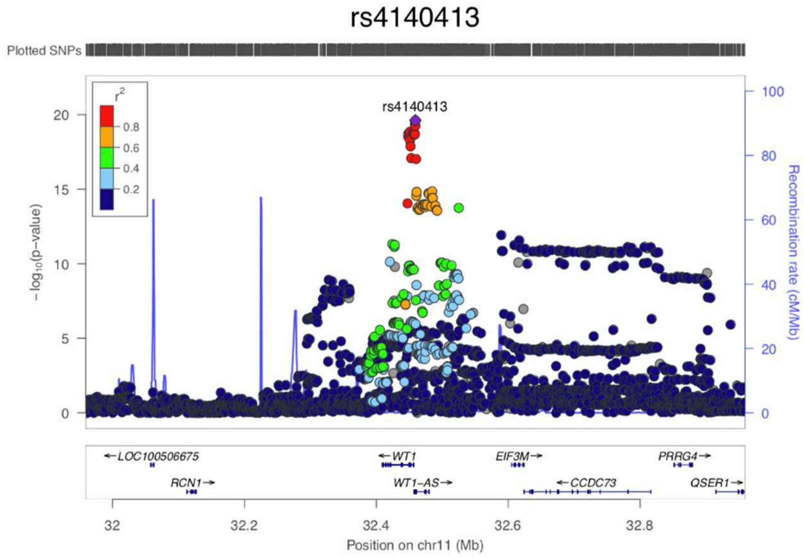
rs4140413
Plotted SNPs
rs4140413
−log10(p−value)
Recombination rate (cM/Mb)
←LOC100506675
←WT1
EIF3M→
PRRG4→
RCN1→
WT1−AS→
←CCDC73
QSER1→
Position on chr11 (Mb)

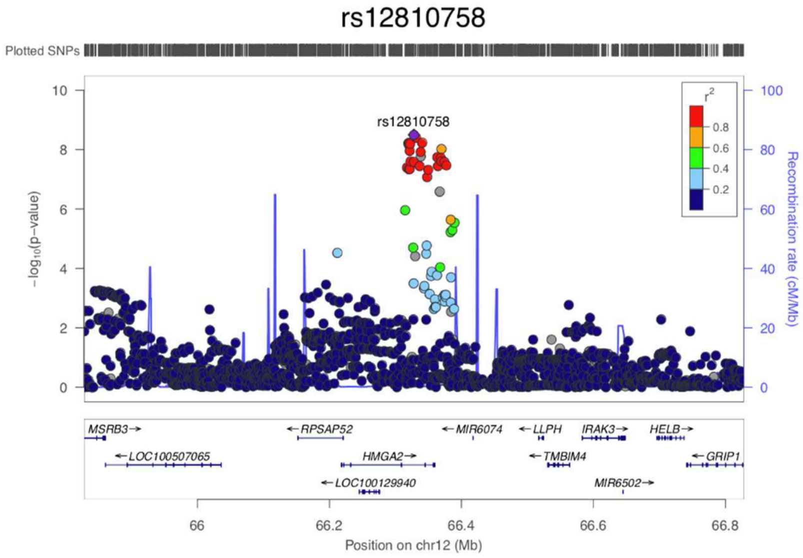
rs12810758
Plotted SNPs
rs12810758
−log10(p−value)
Recombination rate (cM/Mb)
MSRB3→
←RPSAP52
←MIR6074
←LLPH
IRAK3→
HELB→
←LOC100507065
HMGA2→
←TMBIM4
←GRIP1
←LOC100129940
MIR6502→
Position on chr12 (Mb)

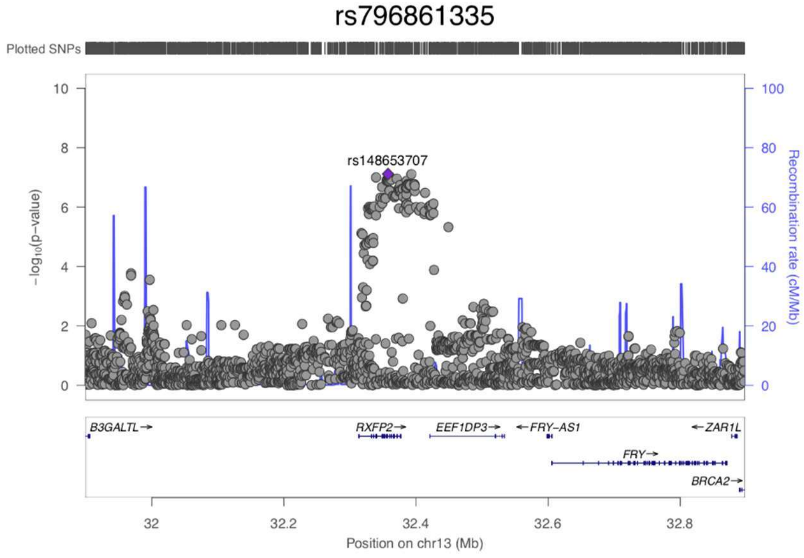
rs796861335
Plotted SNPs
rs148653707
−log10(p−value)
Recombination rate (cM/Mb)
B3GALTL→
RXFP2→
EEF1DP3→
←FRY−AS1
←ZAR1L
FRY→
BRCA2→
Position on chr13 (Mb)

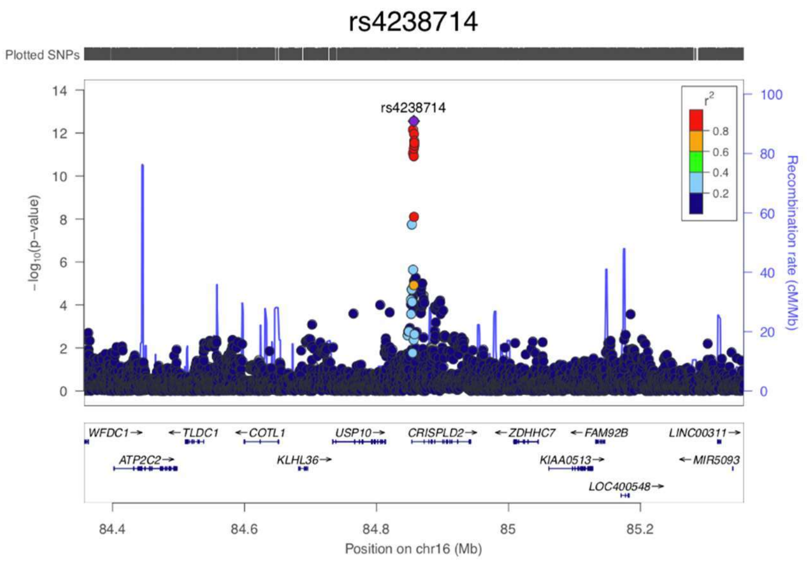

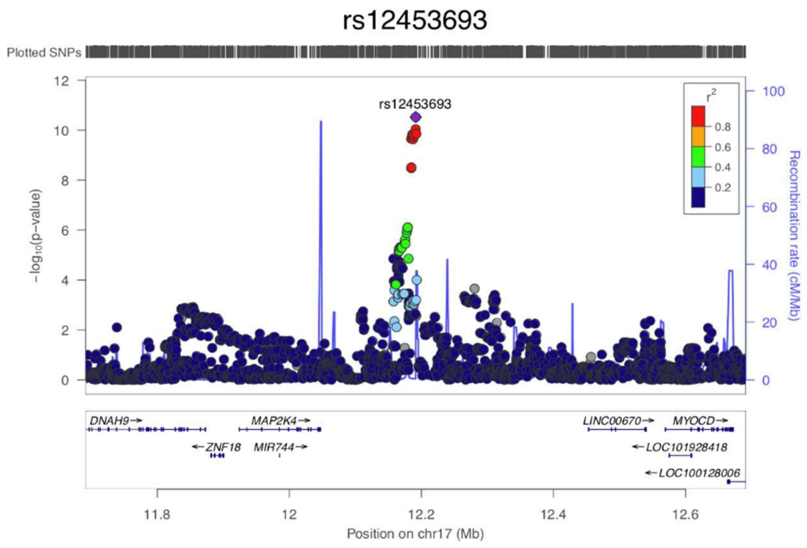

Femoral Hernia

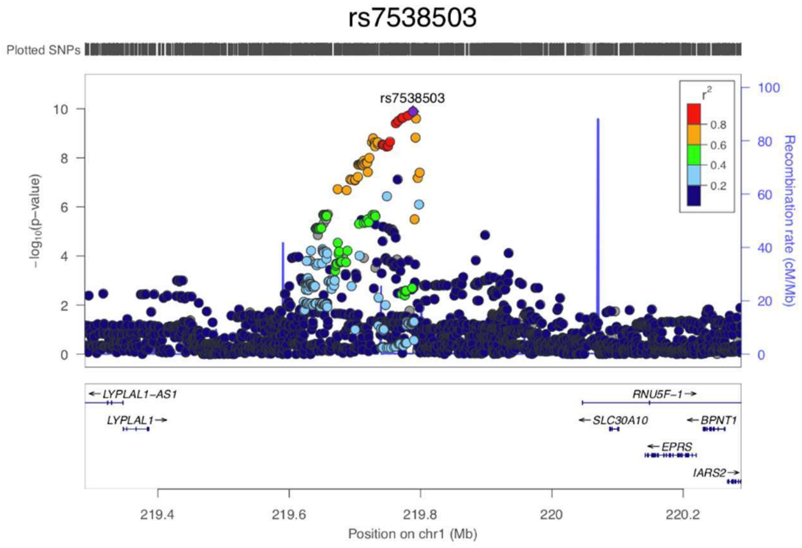

Umbilical Hernia

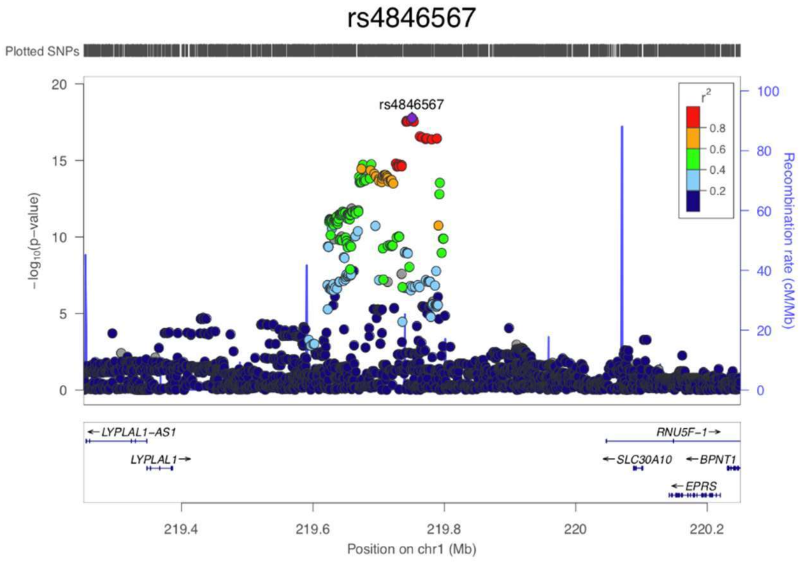

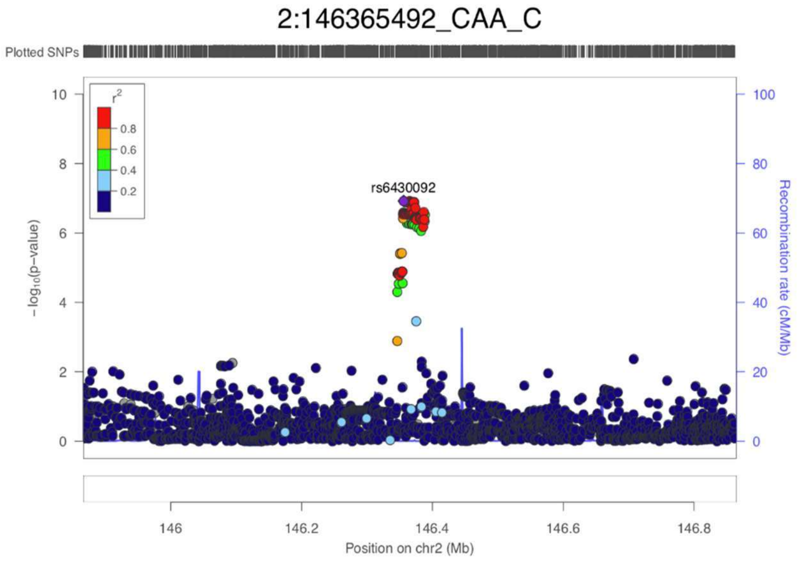

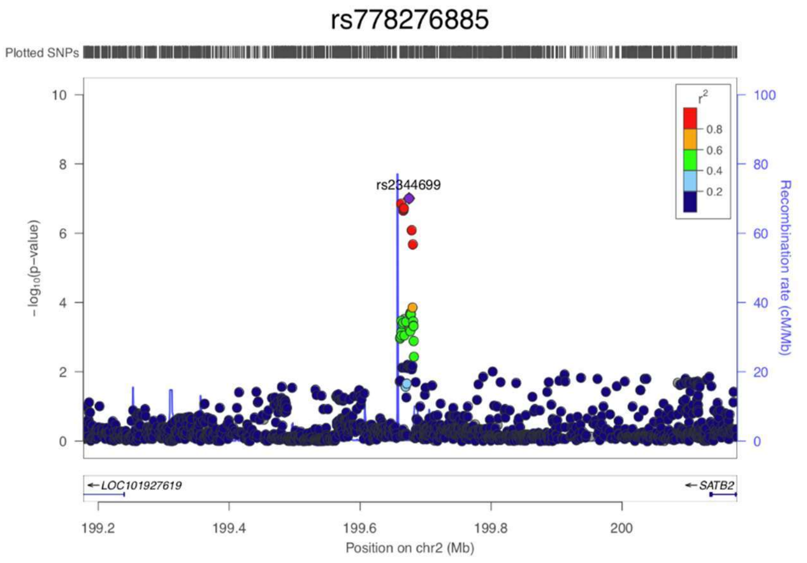

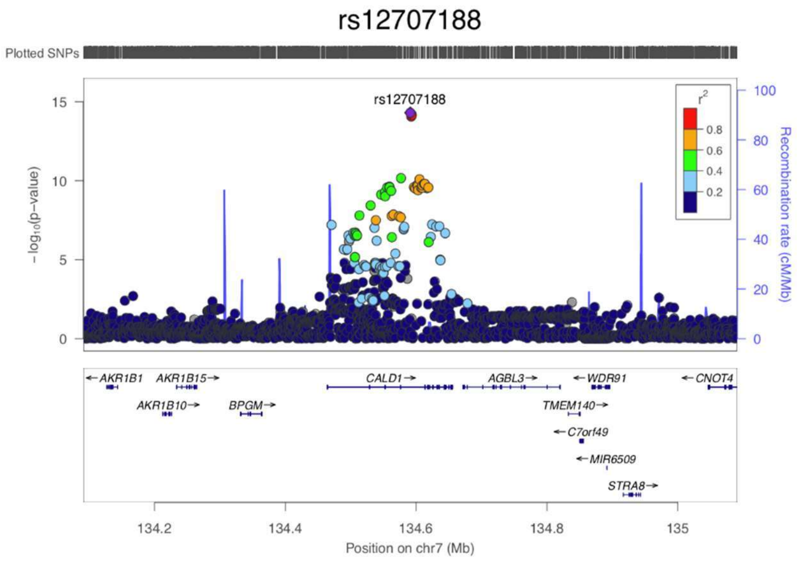

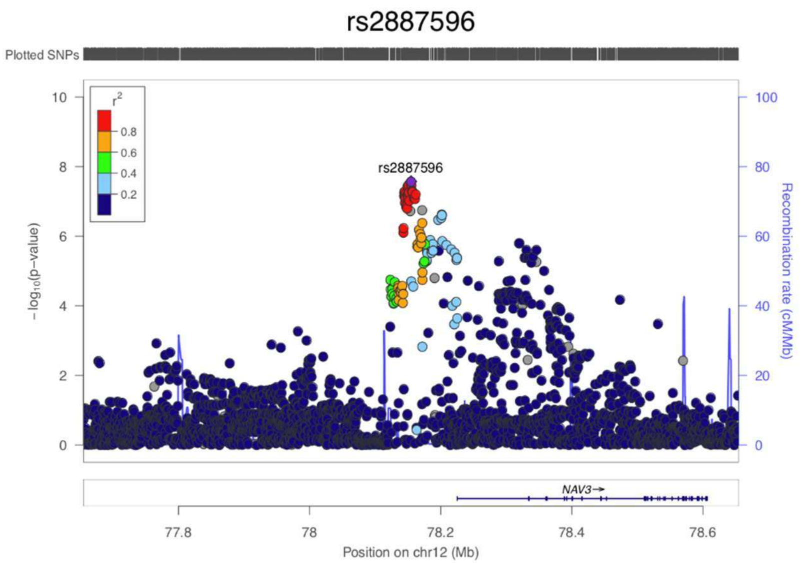

Hiatus Hernia

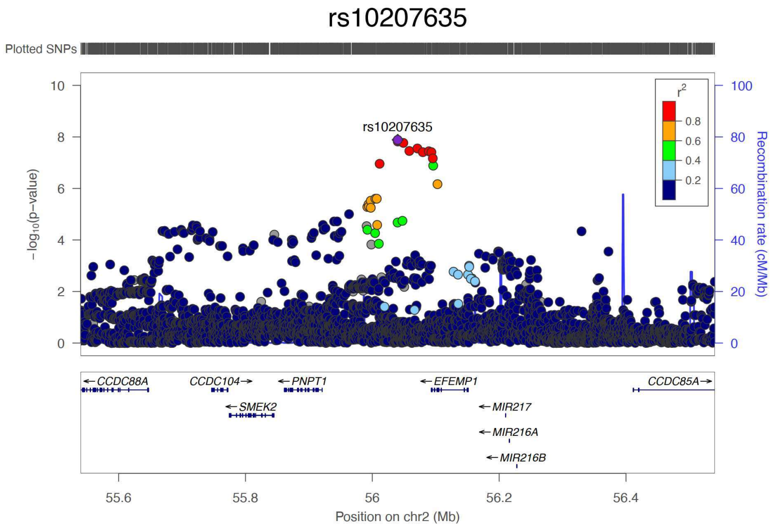

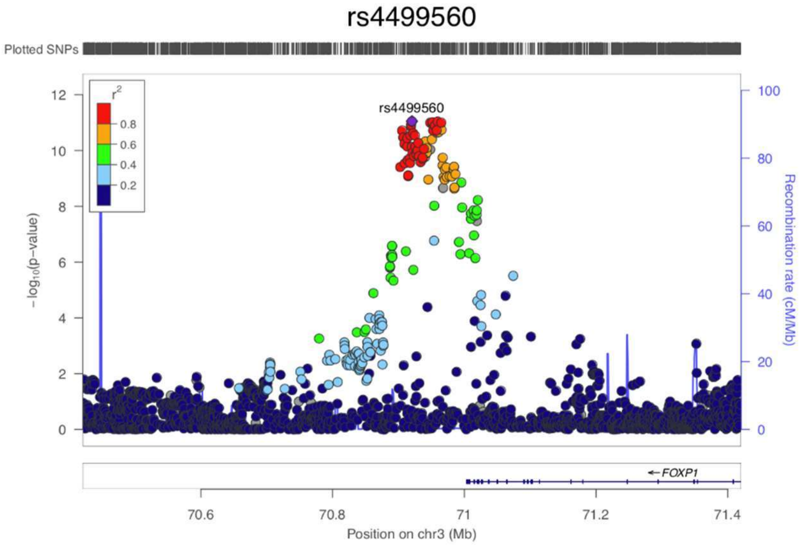

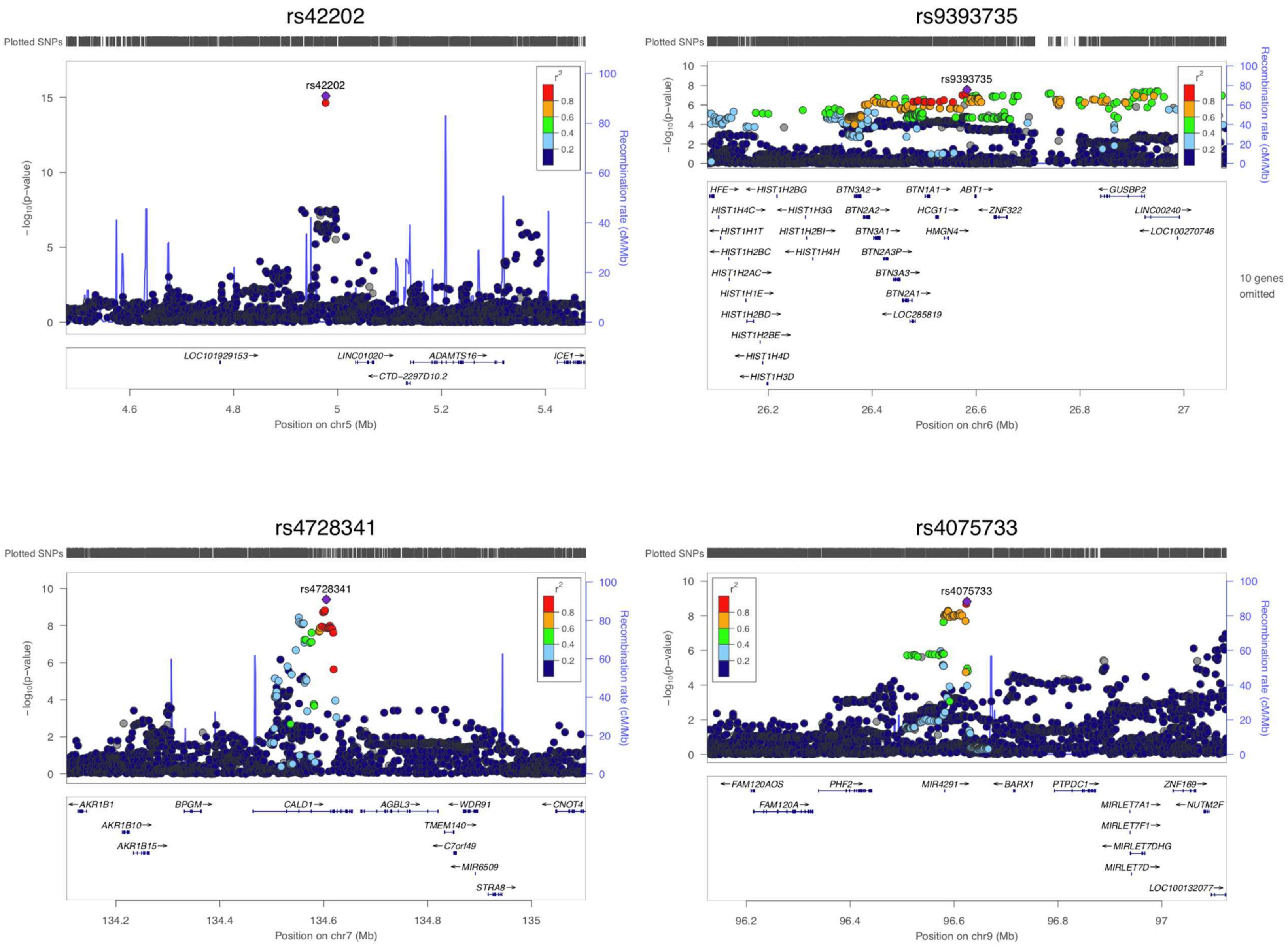
rs42202
Plotted SNPs
−log10(p-value)
Recombination rate (cM/Mb)
LOC101929153→
LINC01020→
ADAMTS16→
ICE1→
←CTD-2297D10.2
Position on chr5 (Mb)
rs9393735
HFE→
←HIST1H2BG
BTN3A2→
BTN1A1→
ABT1→
←GUSBP2
HIST1H4C→
←HIST1H3G
BTN2A2→
HCG11→
←ZNF322
LINC00240→
←HIST1H1T
HIST1H2BI→
BTN3A1→
HMGN4→
←LOC100270746
←HIST1H2BC
←HIST1H4H
BTN2A3P→
HIST1H2AC→
BTN3A3→
HIST1H1E→
BTN2A1→
HIST1H2BD→
←LOC285819
HIST1H2BE→
←HIST1H4D
←HIST1H3D
10 genes omitted
Position on chr6 (Mb)
rs4728341
←AKR1B1
BPGM→
CALD1→
AGBL3→
←WDR91
←CNOT4
AKR1B10→
TMEM140→
AKR1B15→
←C7orf49
←MIR6509
STRA8→
Position on chr7 (Mb)
rs4075733
←FAM120AOS
PHF2→
MIR4291→
←BARX1
PTPDC1→
ZNF169→
FAM120A→
MIRLET7A1→
←NUTM2F
MIRLET7F1→
←MIRLET7DHG
MIRLET7D→
LOC100132077→
Position on chr9 (Mb)

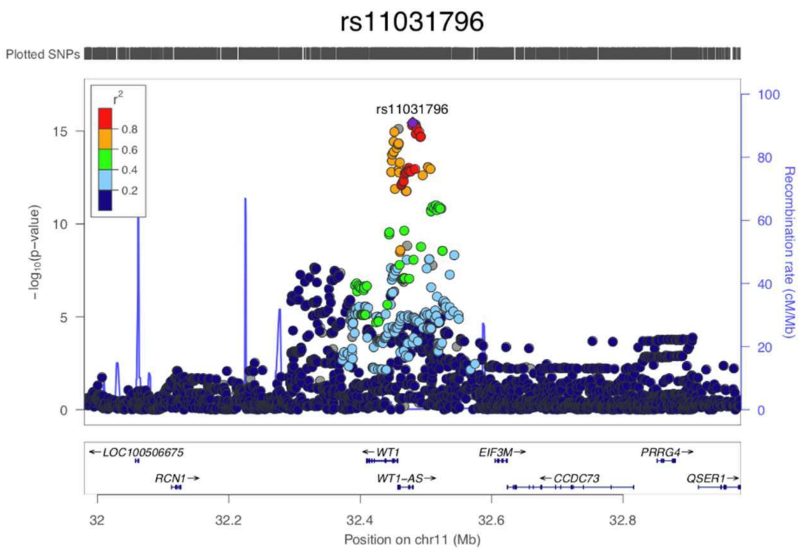
rs11031796
Plotted SNPs
rs11031796
r2
0.8
0.6
0.4
0.2
-log10(p-value)
Recombination rate (cM/Mb)
LOC100506675
RCN1
WT1
WT1-AS
EIF3M
CCDC73
PRRG4
QSER1
Position on chr11 (Mb)

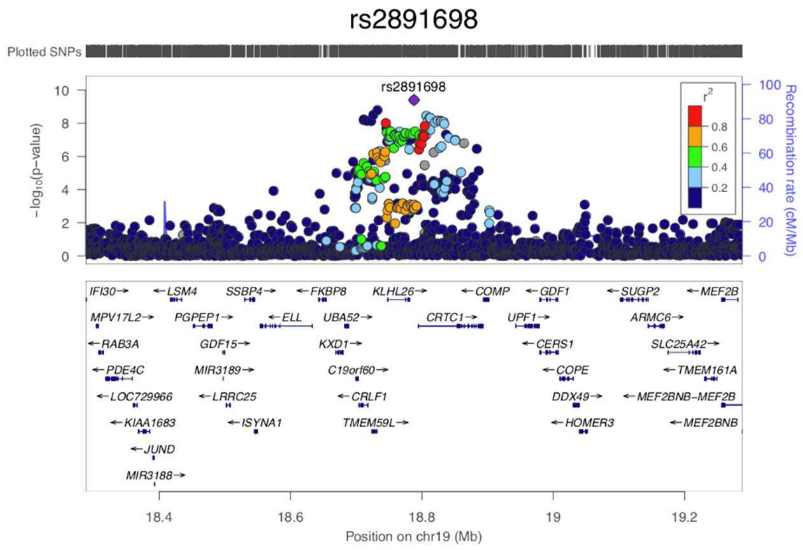
rs2891698
Plotted SNPs
rs2891698
r2
-log10(p-value)
Recombination rate (cM/Mb)
IFI30
LSM4
SSBP4
FKBP8
KLHL26
COMP
GDF1
SUGP2
MEF2B
MPV17L2
PGPEP1
ELL
UBA52
CRTC1
UPF1
ARMC6
RAB3A
GDF15
KXD1
CERS1
SLC25A42
PDE4C
MIR3189
C19orf60
COPE
TMEM161A
LOC729966
LRRC25
CRLF1
DDX49
MEF2BNB-MEF2B
KIAA1683
ISYNA1
TMEM59L
HOMER3
MEF2BNB
JUND
MIR3188
Position on chr19 (Mb)
