# Supplementary material for: Shared genetic architecture of hernias: A genome-wide association study with multivariable meta-analysis of multiple hernia phenotypes
Source: PLoS One. 2022 Dec 30;17(12):e0272261. doi: 10.1371/journal.pone.0272261 (PMC9803250; doi:10.1371/journal.pone.0272261)

S2 Fig 4. Quantile-quantile (Q-Q) plots of A) all overlap hernia associated signals and B) all umbrella hernia associated signals. Across both overlap and umbrella hernia analyses, the λ_{GC} was 1.05 and 1.20, respectively, with an LDSC intercept of 1.01 and 1.03 and an attenuation ratio of 0.15 and 0.10

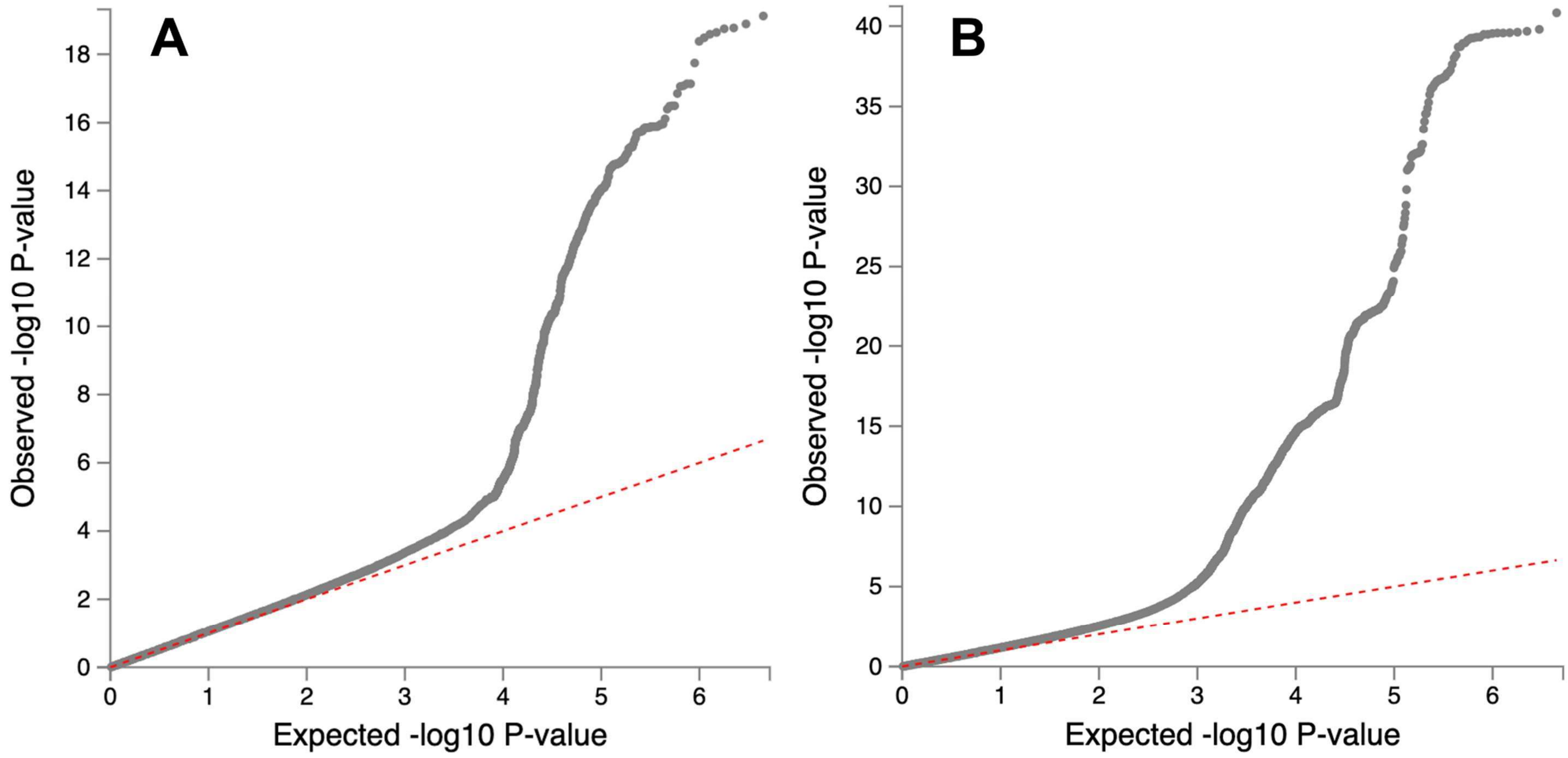

Supplement: S4 Fig — Quantile-quantile (Q-Q) plots of A) all overlap hernia associated signals and B) all umbrella hernia associated signals. Across both overlap and umbrella hernia analyses, the λGC was 1.05 and 1.20, respectively, with an LDSC intercept of 1.01 and 1.03 and an attenuation ratio of 0.15 and 0.10. (PDF) [file pone.0272261.s024.pdf]
