# Supplementary material for: Shared genetic architecture of hernias: A genome-wide association study with multivariable meta-analysis of multiple hernia phenotypes
Source: PLoS One. 2022 Dec 30;17(12):e0272261. doi: 10.1371/journal.pone.0272261 (PMC9803250; doi:10.1371/journal.pone.0272261)
Supplement: S5 Fig — LocusZoom plots of the six independent genome-wide significant SNPs at the four overlap hernia associated susceptibility loci. Plots are ordered by chromosome number and genomic position. SNP position is shown on the x-axis, and strength of association on the y-axis (-log10 P-value). The linkage disequilibrium (LD) relationship between the lead SNP and the surrounding SNPs is indicated by the r2 legend. In the lower panel of each figure, genes within 500kb of the index SNP are shown. The position on each chromosome is shown in relation to Human Genome build hg19. (PDF) [file pone.0272261.s025.pdf]

S2 Fig 5. Regional Locus Zoom plots of all overlap hernia associated signals. LocusZoom plots of the six independent genome-wide significant SNPs at the four overlap hernia associated susceptibility loci. Plots are ordered by chromosome number and genomic position. SNP position is shown on the x-axis, and strength of association on the y-axis (-log10 P-value). The linkage disequilibrium (LD) relationship between the lead SNP and the surrounding SNPs is indicated by the r^2 legend. In the lower panel of each figure, genes within 500kb of the index SNP are shown. The position on each chromosome is shown in relation to Human Genome build hg19.

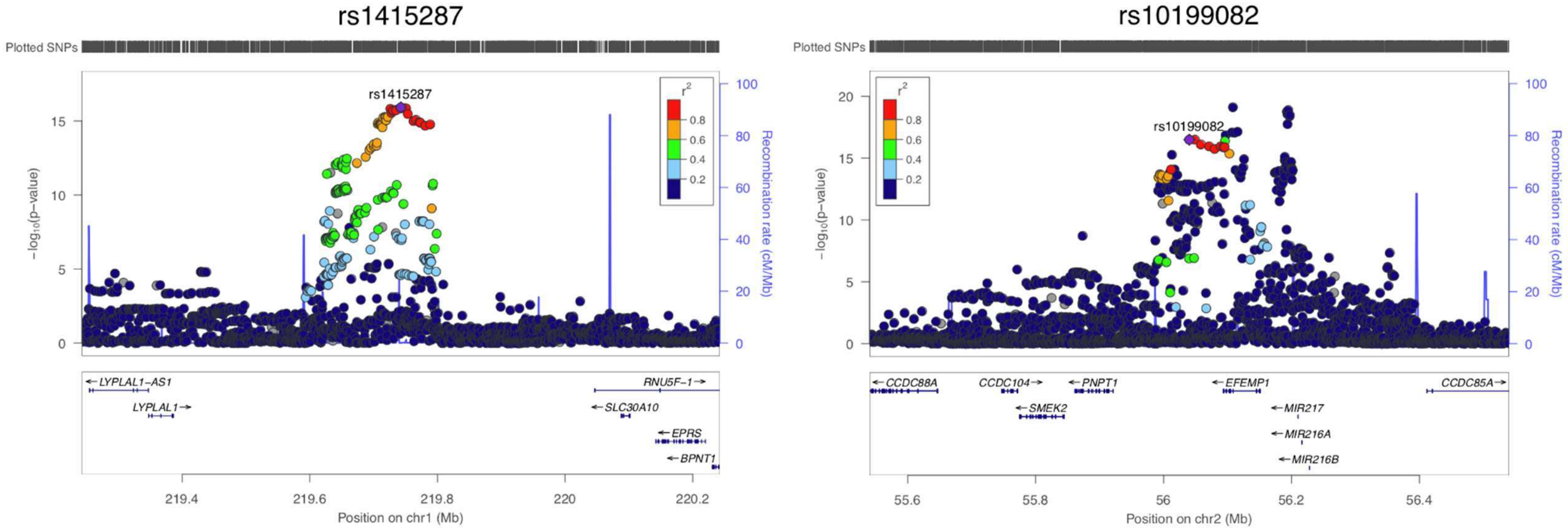

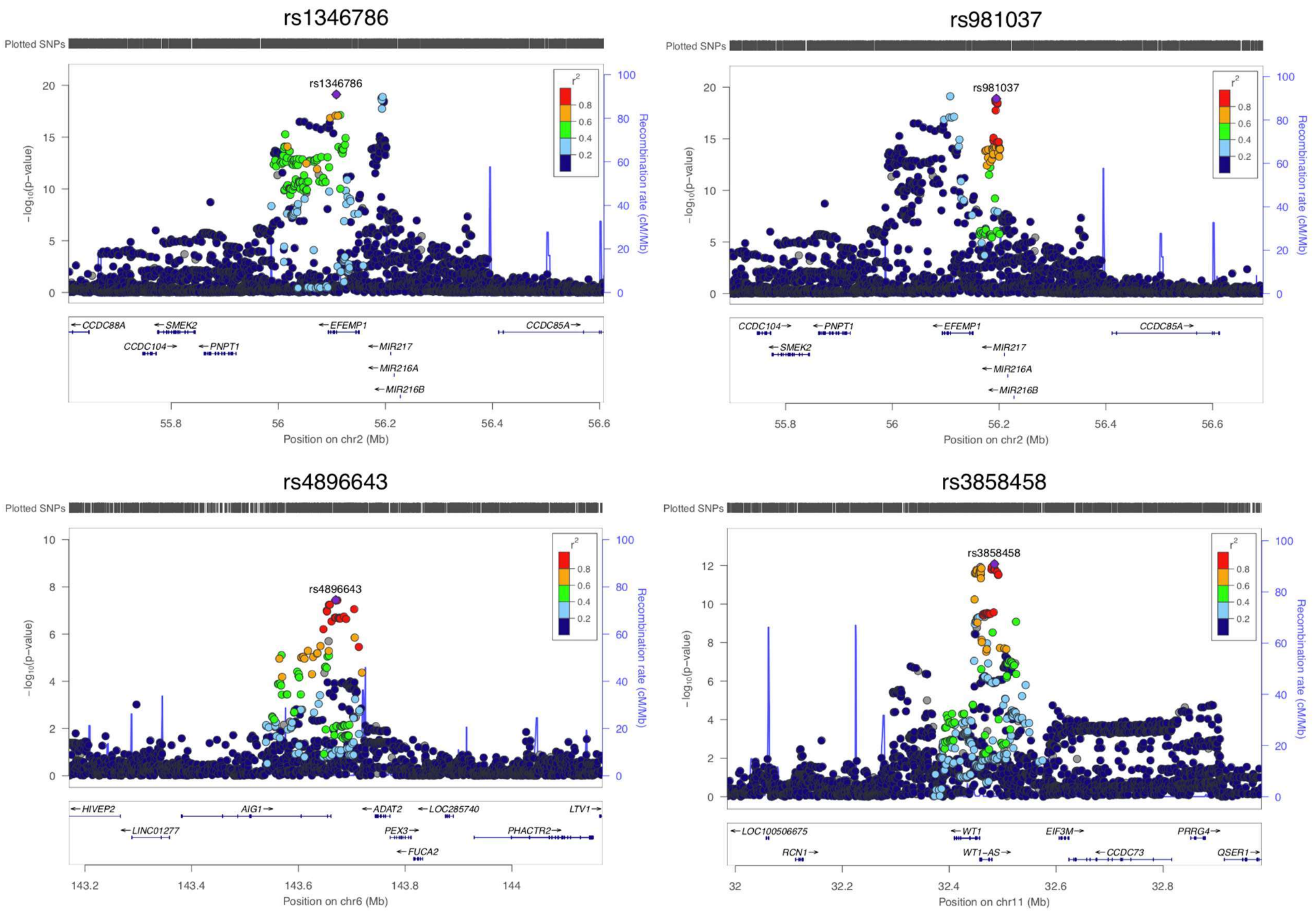
rs1346786
Plotted SNPs
rs1346786
-log10(p-value)
Recombination rate (cM/Mb)
r2
0.8
0.6
0.4
0.2
CCDC88A
SMEK2
CCDC104
PNPT1
EFEMP1
MIR217
MIR216A
MIR216B
CCDC85A
Position on chr2 (Mb)
rs981037
Plotted SNPs
rs981037
-log10(p-value)
Recombination rate (cM/Mb)
CCDC104
PNPT1
SMEK2
EFEMP1
MIR217
MIR216A
MIR216B
CCDC85A
Position on chr2 (Mb)
rs4896643
Plotted SNPs
rs4896643
-log10(p-value)
Recombination rate (cM/Mb)
HIVEP2
LINC01277
AIG1
ADAT2
LOC285740
PEX3
FUCA2
PHACTR2
LTV1
Position on chr6 (Mb)
rs3858458
Plotted SNPs
rs3858458
-log10(p-value)
Recombination rate (cM/Mb)
LOC100506675
RCN1
WT1
WT1-AS
EIF3M
CCDC73
PRRG4
QSER1
Position on chr11 (Mb)
