# Supplementary material for: Shared genetic architecture of hernias: A genome-wide association study with multivariable meta-analysis of multiple hernia phenotypes
Source: PLoS One. 2022 Dec 30;17(12):e0272261. doi: 10.1371/journal.pone.0272261 (PMC9803250; doi:10.1371/journal.pone.0272261)
Supplement: S6 Fig — LocusZoom plots of the 25 independent genome-wide significant SNPs at the 19 hernia-associated susceptibility loci. Plots are ordered by chromosome number and genomic position. SNP position is shown on the x-axis, and strength of association on the y-axis (-log10 P-value). The linkage disequilibrium (LD) relationship between the lead SNP and the surrounding SNPs is indicated by the r2 legend. In the lower panel of each figure, genes within 500kb of the index SNP are shown. The position on each chromosome is shown in relation to Human Genome build hg19. (PDF) [file pone.0272261.s026.pdf]

S2 Fig 6. Regional Locus Zoom plots of all umbrella hernia associated signals. LocusZoom plots of the 25 independent genome-wide significant SNPs at the 19 hernia-associated susceptibility loci. Plots are ordered by chromosome number and genomic position. SNP position is shown on the x-axis, and strength of association on the y-axis (-log10 P-value). The linkage disequilibrium (LD) relationship between the lead SNP and the surrounding SNPs is indicated by the r^2 legend. In the lower panel of each figure, genes within 500kb of the index SNP are shown. The position on each chromosome is shown in relation to Human Genome build hg19.

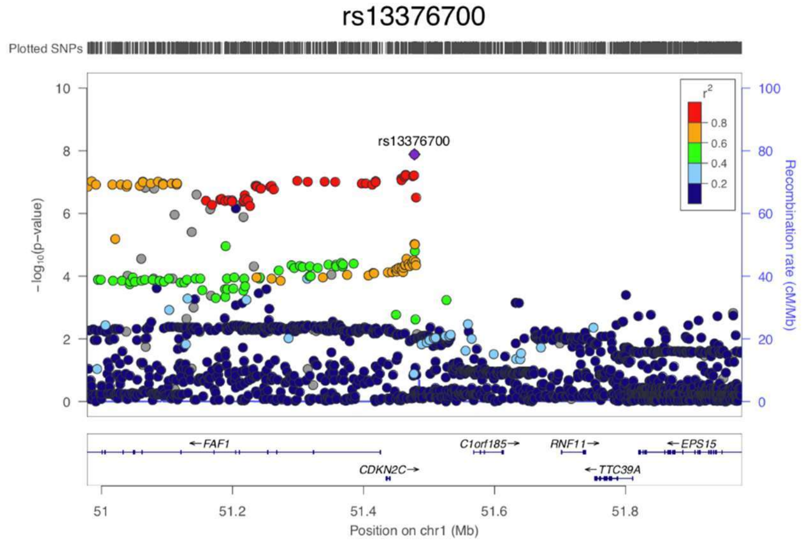

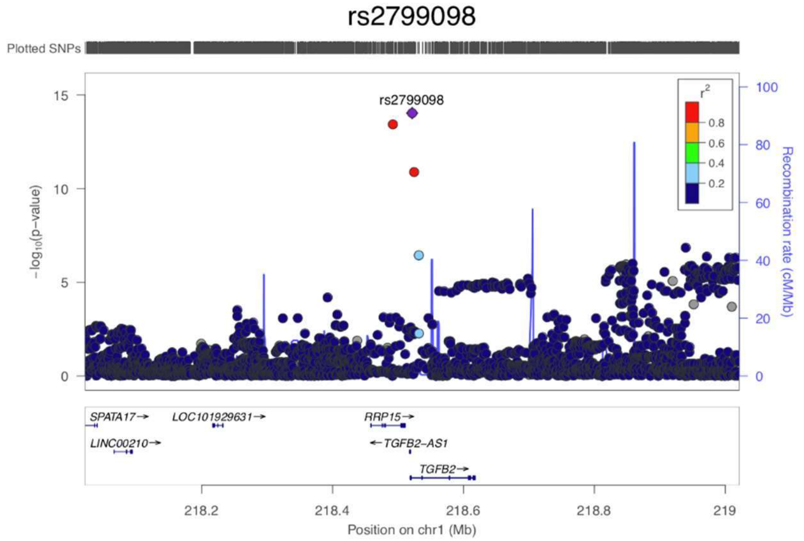

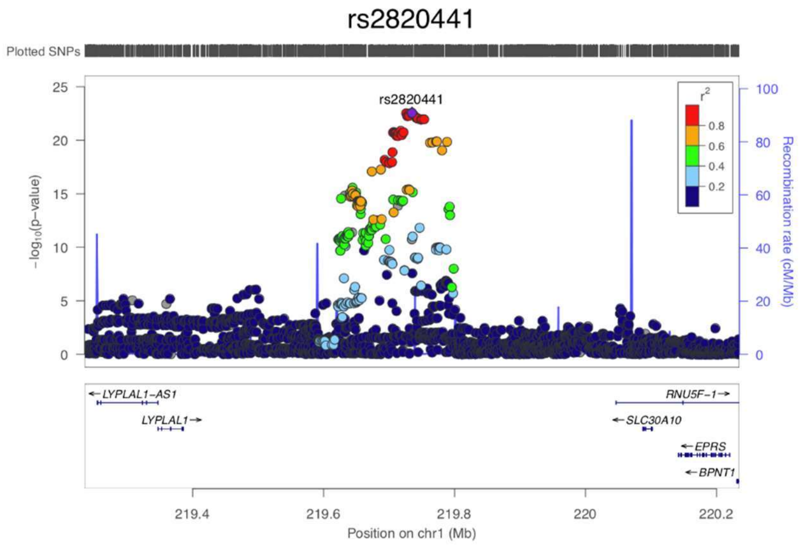
rs2820441
Plotted SNPs
rs2820441
-log10(p-value)
Recombination rate (cM/Mb)
r2
LYPLAL1-AS1
LYPLAL1
RNU5F-1
SLC30A10
EPRS
BPNT1
Position on chr1 (Mb)

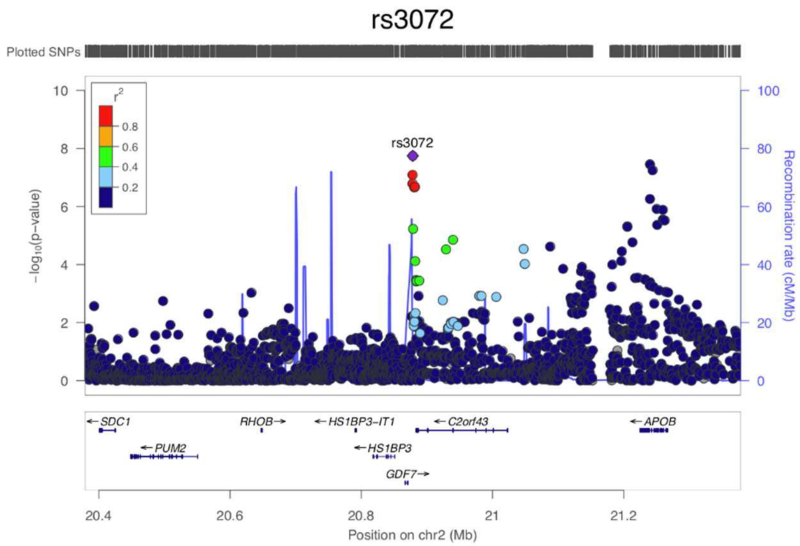
rs3072
Plotted SNPs
rs3072
-log10(p-value)
Recombination rate (cM/Mb)
r2
SDC1
PUM2
RHOB
HS1BP3-IT1
HS1BP3
GDF7
C2orf43
APOB
Position on chr2 (Mb)

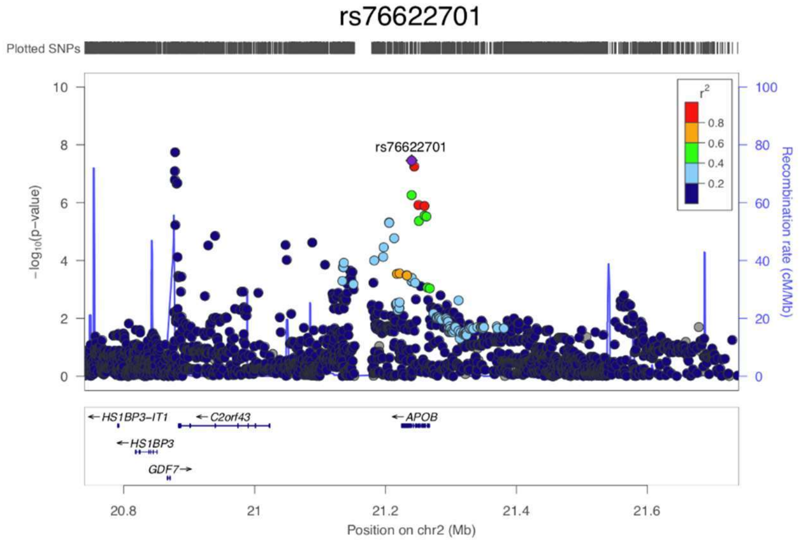
rs76622701
Plotted SNPs
rs76622701
-log10(p-value)
Recombination rate (cM/Mb)
r2
HS1BP3-IT1
HS1BP3
GDF7
C2orf43
APOB
Position on chr2 (Mb)

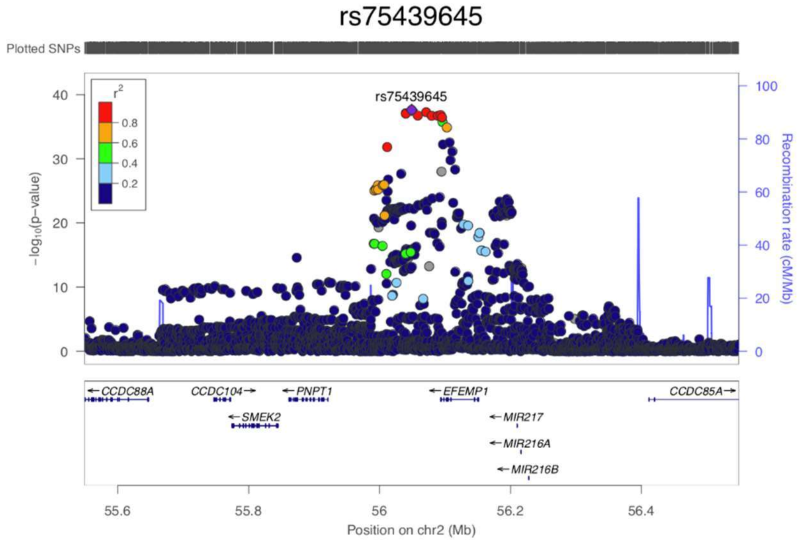
rs75439645
Plotted SNPs
rs75439645
-log10(p-value)
Recombination rate (cM/Mb)
r2
CCDC88A
CCDC104
SMEK2
PNPT1
EFEMP1
MIR217
MIR216A
MIR216B
CCDC85A
Position on chr2 (Mb)

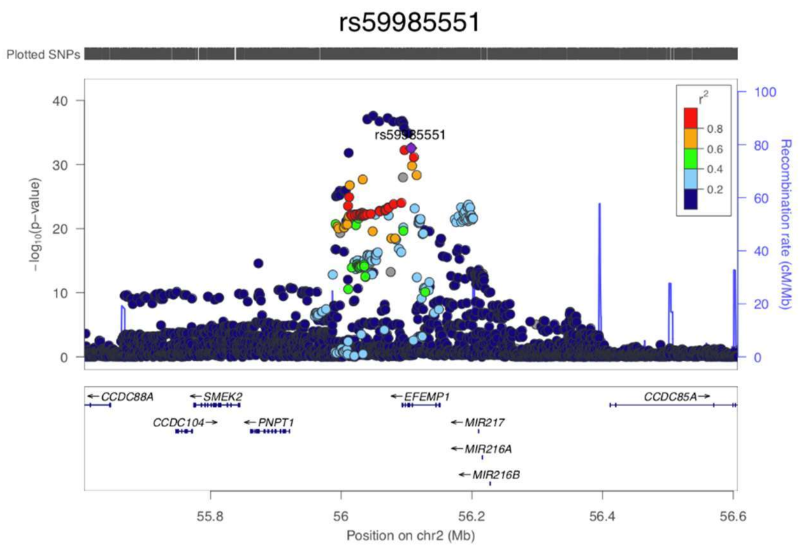
rs59985551
Plotted SNPs
−log10(p−value)
Recombination rate (cM/Mb)
r2
CCDC88A
SMEK2
CCDC104
PNPT1
EFEMP1
MIR217
MIR216A
MIR216B
CCDC85A
Position on chr2 (Mb)

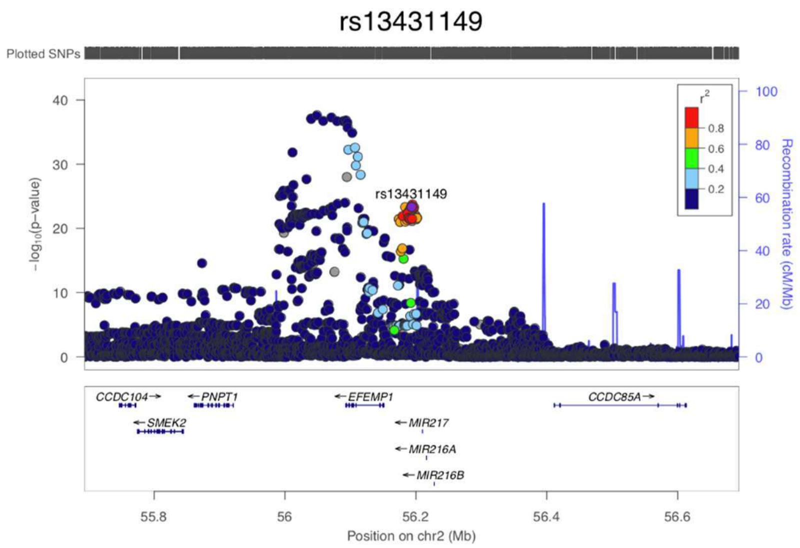
rs13431149
Plotted SNPs
−log10(p−value)
Recombination rate (cM/Mb)
r2
CCDC104
PNPT1
SMEK2
EFEMP1
MIR217
MIR216A
MIR216B
CCDC85A
Position on chr2 (Mb)

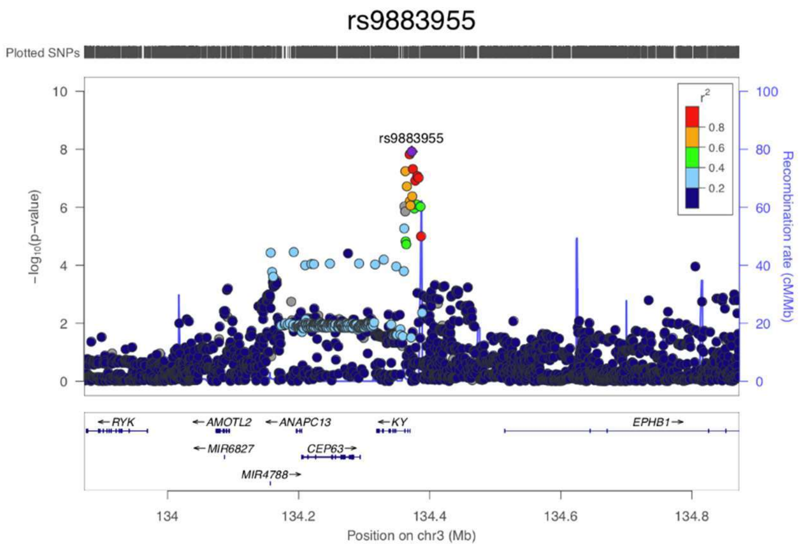
rs9883955
Plotted SNPs
−log10(p−value)
Recombination rate (cM/Mb)
r2
RYK
AMOTL2
ANAPC13
KY
EPHB1
MIR6827
CEP63
MIR4788
Position on chr3 (Mb)

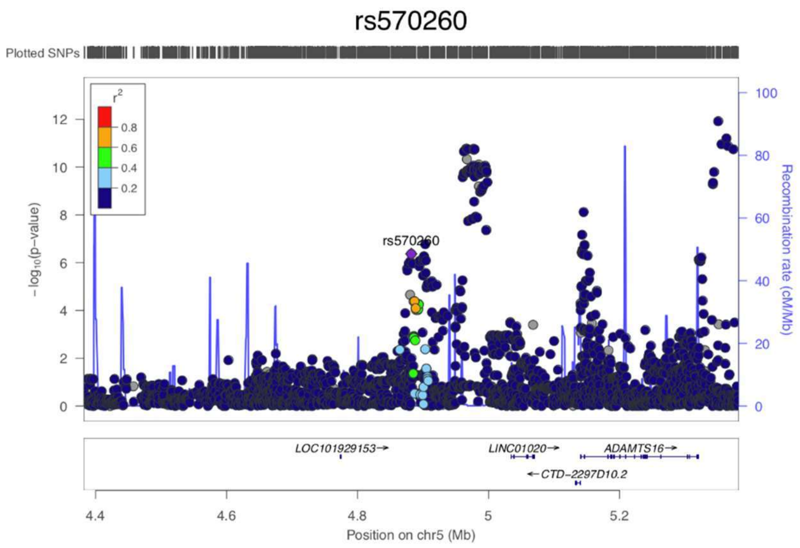
rs570260
Plotted SNPs
−log10(p−value)
Recombination rate (cM/Mb)
r2
LOC101929153
LINC01020
ADAMTS16
CTD−2297D10.2
Position on chr5 (Mb)

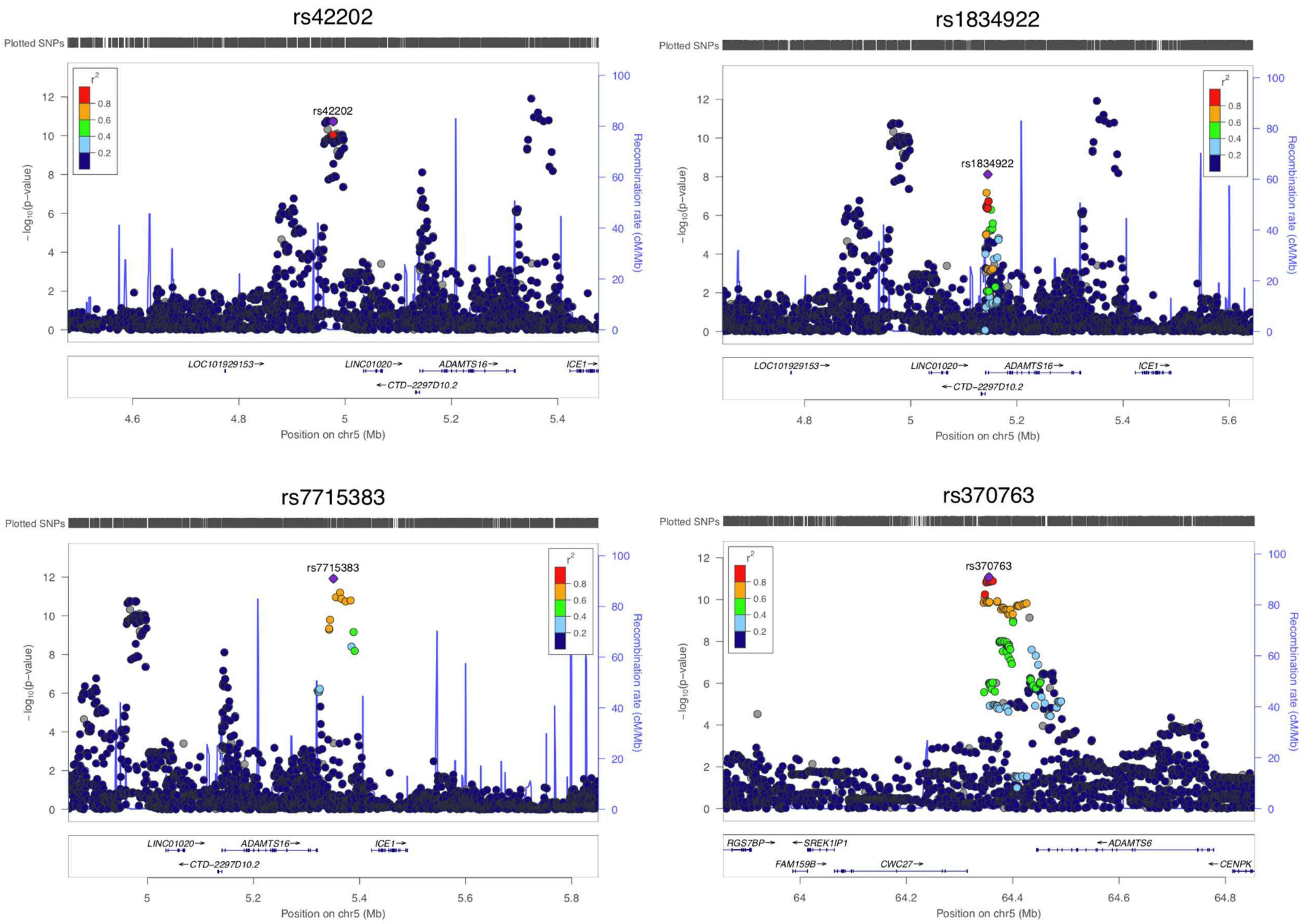
rs42202
Plotted SNPs
rs42202
r²
0.8
0.6
0.4
0.2
−log10(p−value)
Recombination rate (cM/Mb)
LOC101929153→
LINC01020→
ADAMTS16→
ICE1→
←CTD−2297D10.2
Position on chr5 (Mb)
rs1834922
Plotted SNPs
rs1834922
Position on chr5 (Mb)
rs7715383
Plotted SNPs
rs7715383
Position on chr5 (Mb)
rs370763
Plotted SNPs
rs370763
RGS7BP→
←SREK1IP1
FAM159B→
CWC27→
←ADAMTS6
←CENPK
Position on chr5 (Mb)

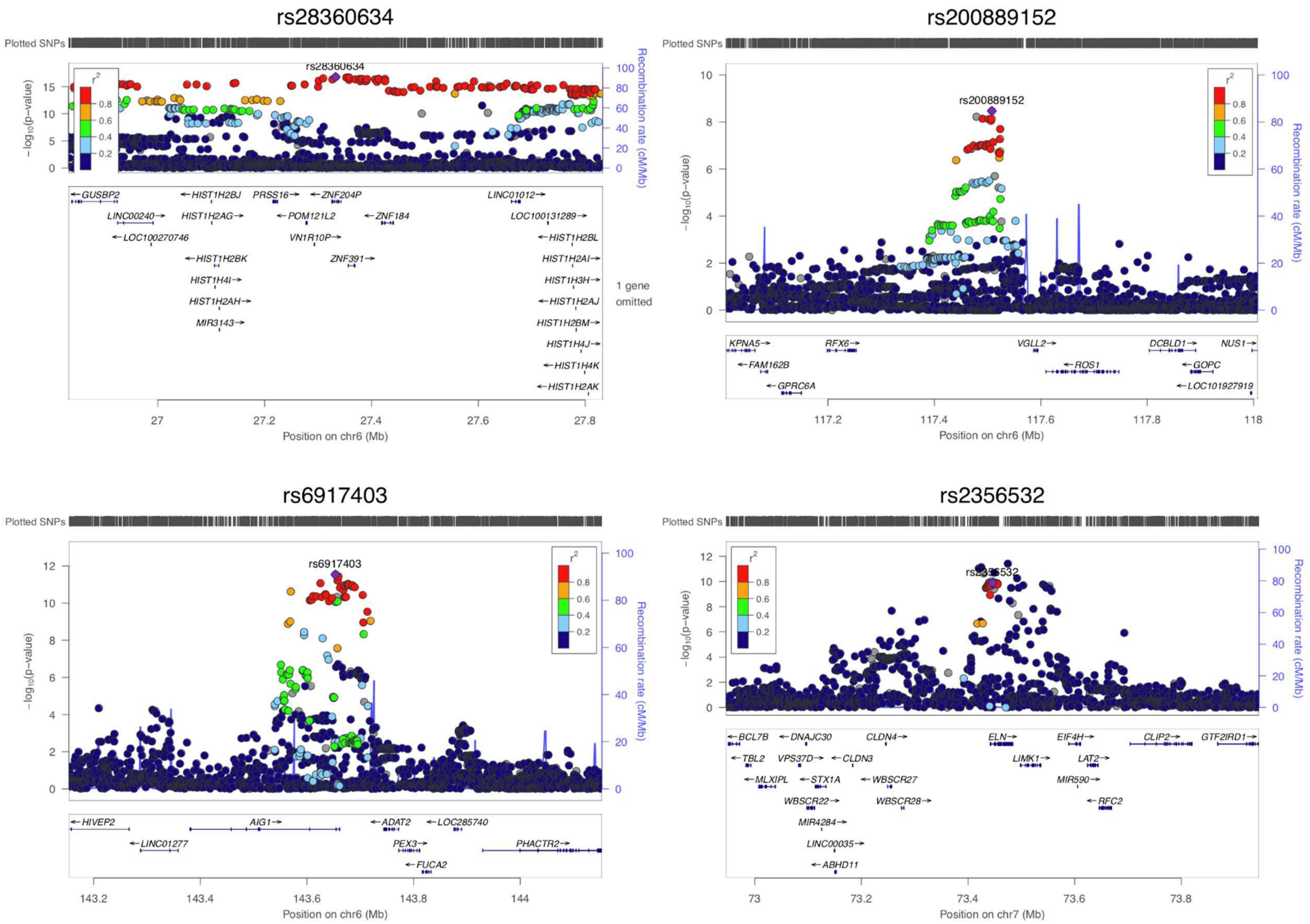
rs28360634
Plotted SNPs
−log10(p−value)
Recombination rate (cM/Mb)
r2
0.8
0.6
0.4
0.2
GUSBP2
HIST1H2BJ
PRSS16
ZNF204P
LINC01012
LINC00240
HIST1H2AG
POM121L2
ZNF184
LOC100131289
LOC100270746
VN1R10P
HIST1H2BL
HIST1H2BK
ZNF391
HIST1H2AI
HIST1H4I
HIST1H3H
HIST1H2AH
HIST1H2AJ
MIR3143
HIST1H2BM
HIST1H4J
HIST1H4K
HIST1H2AK
1 gene omitted
Position on chr6 (Mb)
rs200889152
KPNA5
RFX6
VGLL2
DCBLD1
NUS1
FAM162B
ROS1
GOPC
GPRC6A
LOC101927919
Position on chr6 (Mb)
rs6917403
HIVEP2
AIG1
ADAT2
LOC285740
LINC01277
PEX3
PHACTR2
FUCA2
Position on chr6 (Mb)
rs2356532
BCL7B
DNAJC30
CLDN4
ELN
EIF4H
CLIP2
GTF2IRD1
TBL2
VPS37D
CLDN3
LIMK1
LAT2
MLXIPL
STX1A
WBSCR27
MIR590
WBSCR22
WBSCR28
RFC2
MIR4284
LINC00035
ABHD11
Position on chr7 (Mb)

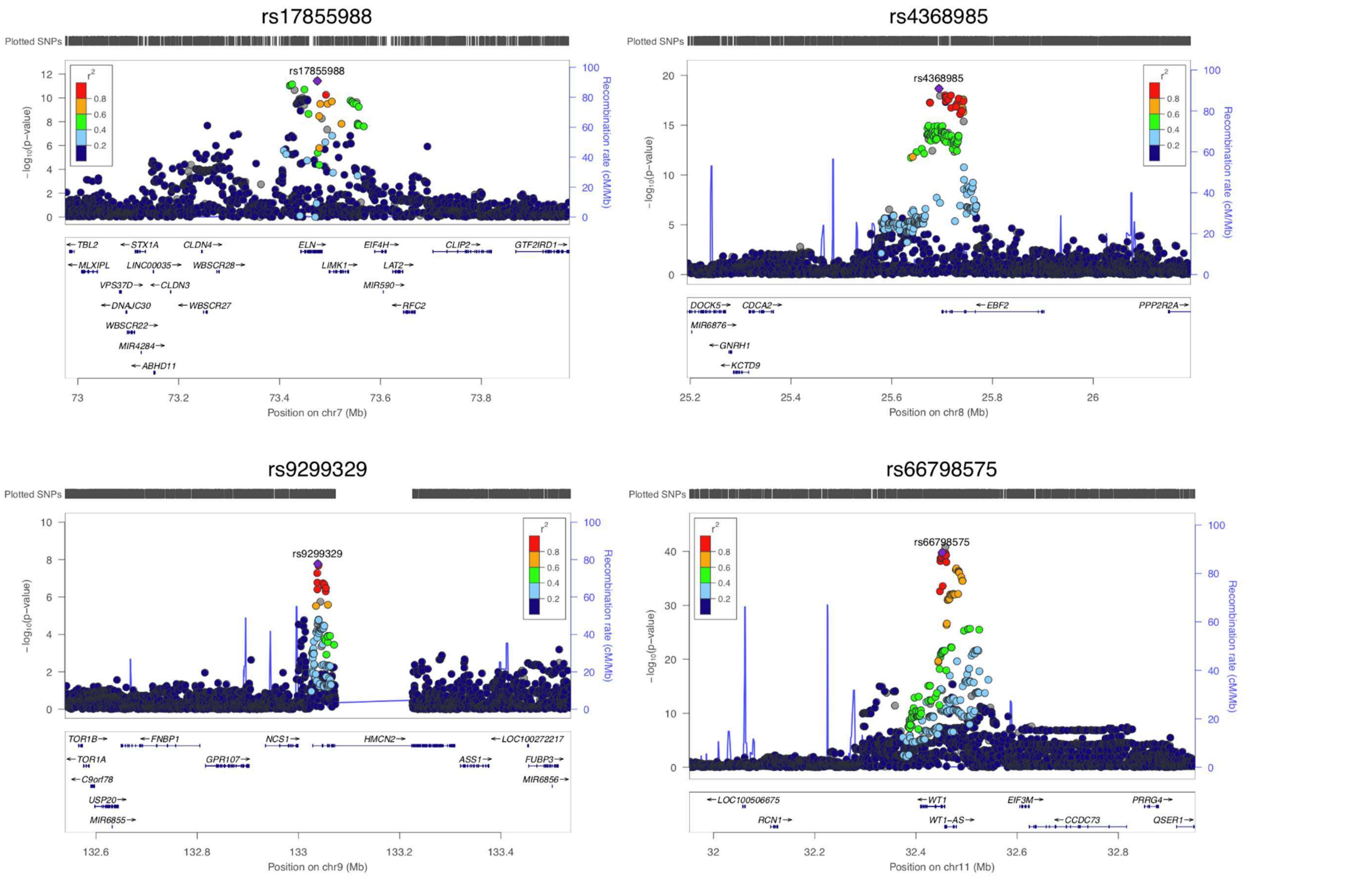
rs17855988
Plotted SNPs
−log10(p−value)
Recombination rate (cM/Mb)
r2
0.8
0.6
0.4
0.2
TBL2
STX1A
CLDN4
ELN
EIF4H
CLIP2
GTF2IRD1
MLXIPL
LINC00035
WBSCR28
LIMK1
LAT2
VPS37D
CLDN3
MIR590
DNAJC30
WBSCR27
RFC2
WBSCR22
MIR4284
ABHD11
Position on chr7 (Mb)
rs4368985
DOCK5
CDCA2
EBF2
PPP2R2A
MIR6876
GNRH1
KCTD9
Position on chr8 (Mb)
rs9299329
TOR1B
FNBP1
NCS1
HMCN2
LOC100272217
TOR1A
GPR107
ASS1
FUBP3
C9orf78
MIR6856
USP20
MIR6855
Position on chr9 (Mb)
rs66798575
LOC100506675
WT1
EIF3M
PRRG4
RCN1
WT1-AS
CCDC73
QSER1
Position on chr11 (Mb)

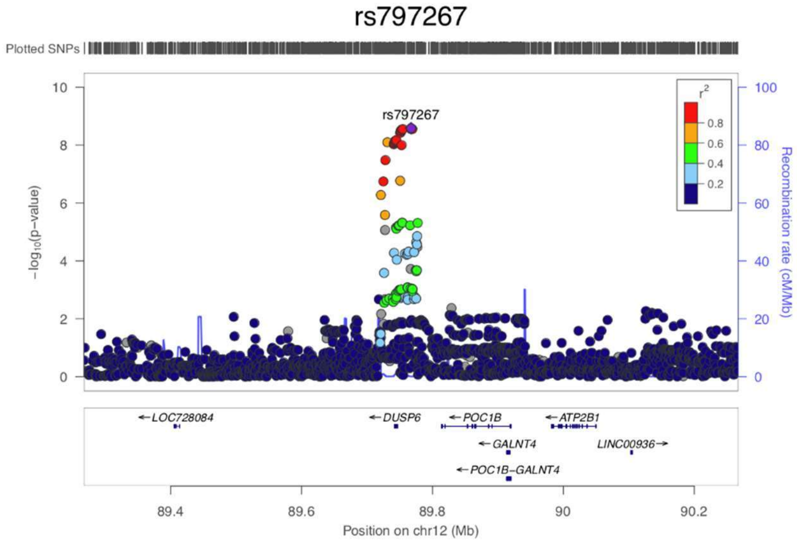
rs797267
Plotted SNPs
rs797267
r²
0.8
0.6
0.4
0.2
−log10(p−value)
Recombination rate (cM/Mb)
LOC728084
DUSP6
POC1B
ATP2B1
GALNT4
LINC00936
POC1B−GALNT4
89.4
89.6
89.8
90
90.2
Position on chr12 (Mb)

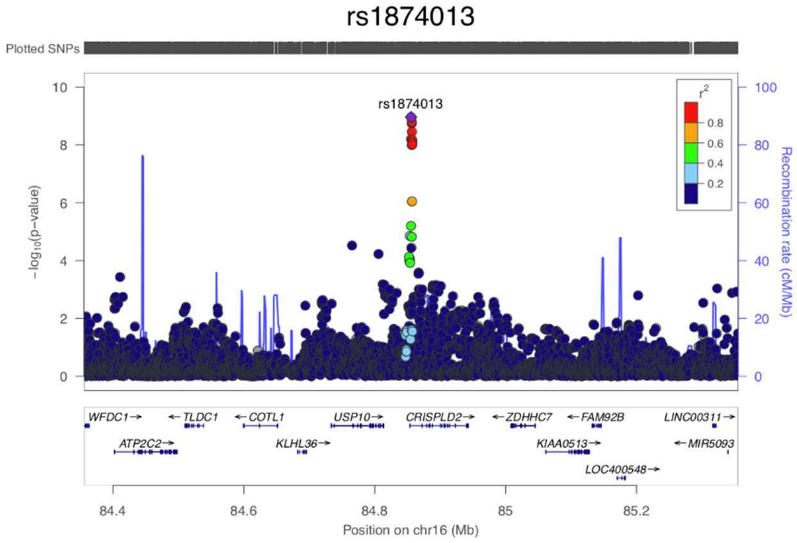
rs1874013
Plotted SNPs
rs1874013
r²
0.8
0.6
0.4
0.2
−log10(p−value)
Recombination rate (cM/Mb)
WFDC1
TLDC1
COTL1
USP10
CRISPLD2
ZDHHC7
FAM92B
LINC00311
ATP2C2
KLHL36
KIAA0513
MIR5093
LOC400548
84.4
84.6
84.8
85
85.2
Position on chr16 (Mb)

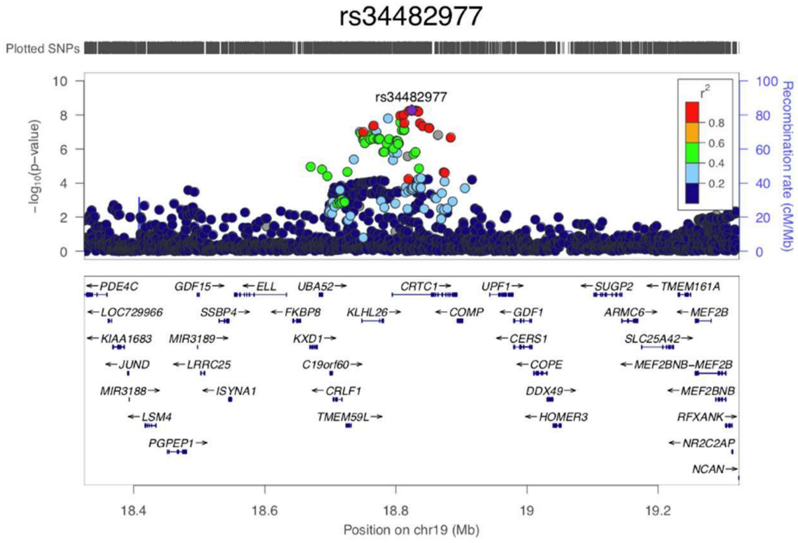
rs34482977
Plotted SNPs
rs34482977
r²
0.8
0.6
0.4
0.2
−log10(p−value)
Recombination rate (cM/Mb)
PDE4C
GDF15
ELL
UBA52
CRTC1
UPF1
SUGP2
TMEM161A
LOC729966
SSBP4
FKBP8
KLHL26
COMP
GDF1
ARMC6
MEF2B
KIAA1683
MIR3189
KXD1
CERS1
SLC25A42
JUND
LRRC25
C19orf60
COPE
MEF2BNB−MEF2B
MIR3188
ISYNA1
CRLF1
DDX49
MEF2BNB
LSM4
TMEM59L
HOMER3
RFXANK
PGPEP1
NR2C2AP
NCAN
18.4
18.6
18.8
19
19.2
Position on chr19 (Mb)
