# Supplementary material for: Shared genetic architecture of hernias: A genome-wide association study with multivariable meta-analysis of multiple hernia phenotypes
Source: PLoS One. 2022 Dec 30;17(12):e0272261. doi: 10.1371/journal.pone.0272261 (PMC9803250; doi:10.1371/journal.pone.0272261)
Supplement: S7 Fig — MAGMA Tissue Expression Analysis of the umbrella hernia GWAS-summary data, implemented in FUMA in A) 30 general and B) 54 specific tissue types. This analysis tests the relationship between highly-expressed genes in a specific tissue and the genetic associations from the GWAS. Gene-property analysis is performed using average expression of genes per tissue type as a gene covariate. Gene expression values are log2-transformed average RPKM (Read Per Kilobase Per Million) per tissue type after winsorization at 50, and are based on GTEx v8 RNA-Seq data across 54 specific tissue types and 30 general tissue types. The dotted line indicates the Bonferroni-corrected α level, and the tissues that meet this significance threshold are highlighted in red. (PDF) [file pone.0272261.s027.pdf]

S2 Fig 7. MAGMA tissue expression analysis of umbrella hernia. MAGMA Tissue Expression Analysis of the umbrella hernia GWAS-summary data, implemented in FUMA in A) 30 general and B) 54 specific tissue types. This analysis tests the relationship between highly-expressed genes in a specific tissue and the genetic associations from the GWAS. Gene-property analysis is performed using average expression of genes per tissue type as a gene covariate. Gene expression values are log2-transformed average RPKM (Read Per Kilobase Per Million) per tissue type after winsorization at 50, and are based on GTEx v8 RNA-Seq data across 54 specific tissue types and 30 general tissue types. The dotted line indicates the Bonferroni-corrected α level, and the tissues that meet this significance threshold are highlighted in red.

A)

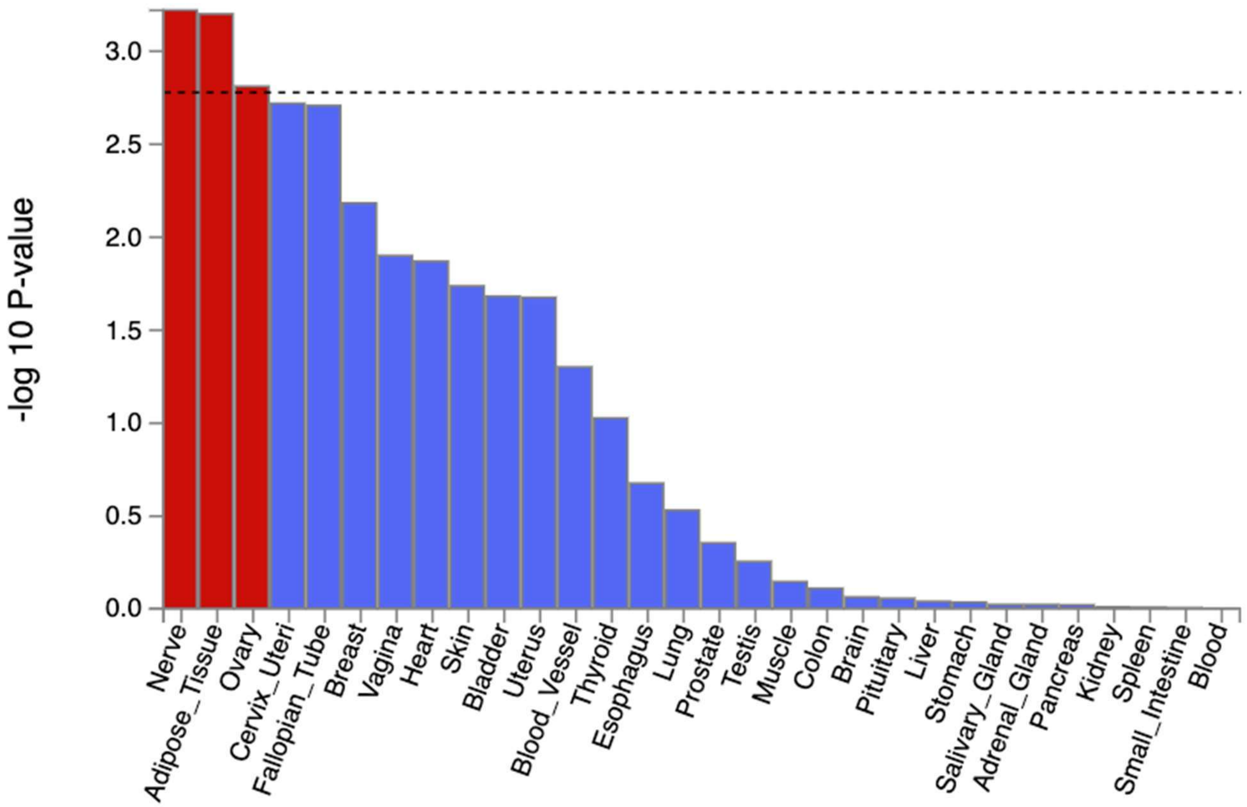

B)

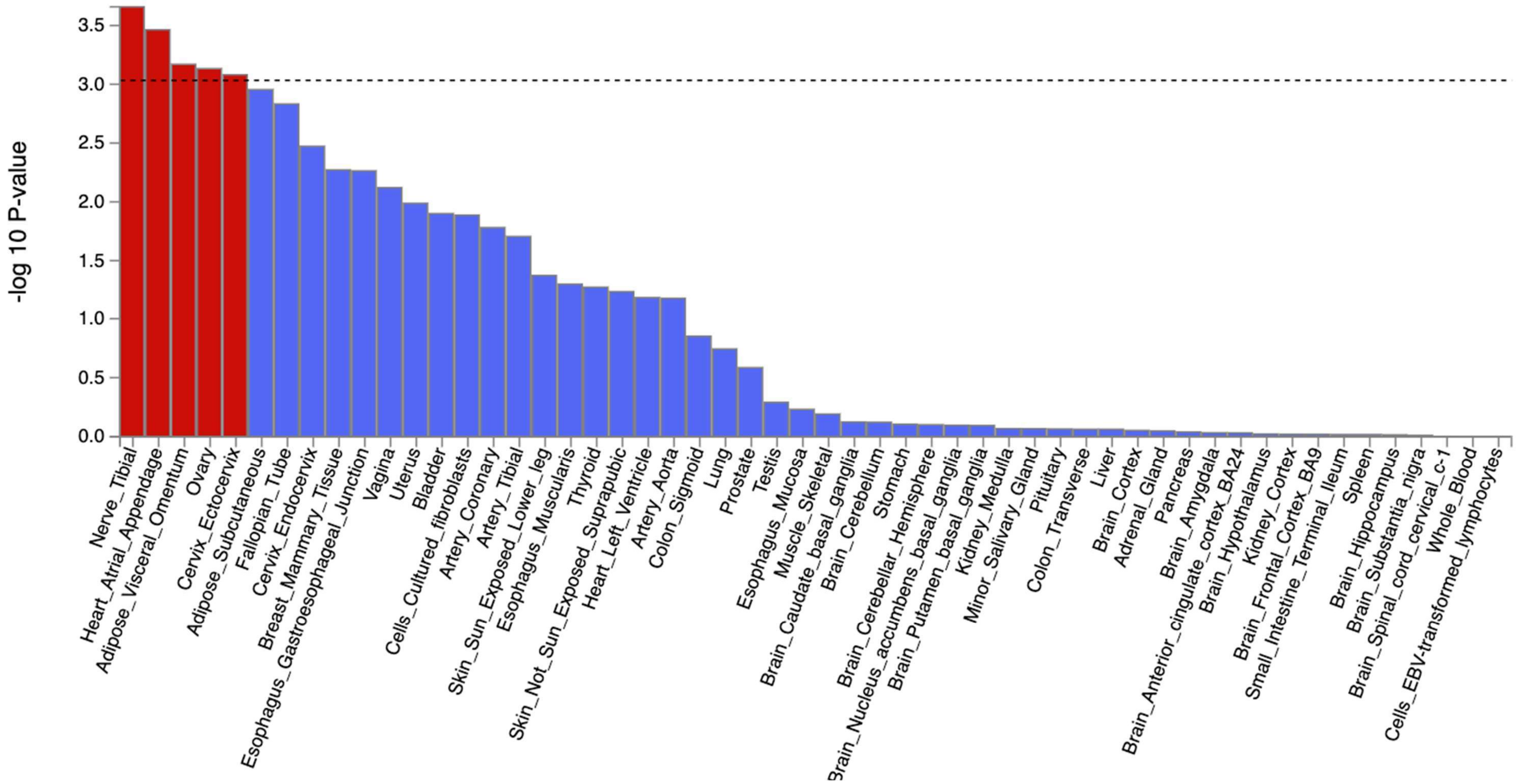
-log 10 P-value
0.0
0.5
1.0
1.5
2.0
2.5
3.0
3.5
Nerve_Tibial
Heart_Atrial_Appendage
Adipose_Visceral_Omentum
Ovary
Cervix_Ectocervix
Adipose_Subcutaneous
Fallopian_Tube
Cervix_Endocervix
Breast_Mammary_Tissue
Esophagus_Gastroesophageal_Junction
Vagina
Uterus
Bladder
Cells_Cultured_fibroblasts
Artery_Coronary
Artery_Tibial
Skin_Sun_Exposed_Lower_leg
Esophagus_Muscularis
Thyroid
Skin_Not_Sun_Exposed_Suprapubic
Heart_Left_Ventricle
Artery_Aorta
Colon_Sigmoid
Lung
Prostate
Testis
Esophagus_Mucosa
Muscle_Skeletal
Brain_Caudate_basal_ganglia
Brain_Cerebellum
Stomach
Brain_Cerebellar_Hemisphere
Brain_Nucleus_accumbens_basal_ganglia
Brain_Putamen_basal_ganglia
Kidney_Medulla
Minor_Salivary_Gland
Pituitary
Colon_Transverse
Liver
Brain_Cortex
Adrenal_Gland
Pancreas
Brain_Amygdala
Brain_Anterior_cingulate_cortex_BA24
Brain_Hypothalamus
Kidney_Cortex
Brain_Frontal_Cortex_BA9
Small_Intestine_Terminal_Ileum
Spleen
Brain_Hippocampus
Brain_Substantia_nigra
Brain_Spinal_cord_cervical_c-1
Whole_Blood
Cells_EBV-transformed_lymphocytes
